# Interleukin 6 (rs1800795) gene polymorphism is associated with cardiovascular diseases: a meta-analysis of 74 studies with 86,229 subjects

**DOI:** 10.17179/excli2019-1248

**Published:** 2019-06-07

**Authors:** Thelma Beatriz González-Castro, Yazmín Hernández-Díaz, Nonanzit Pérez-Hernández, Carlos Alfonso Tovilla-Zárate, Isela Esther Juárez-Rojop, María Lilia López-Narvaez, Ruben Blachman-Braun, Rosalinda Posadas-Sánchez, Gilberto Vargas-Alarcón, Esbeidy García-Flores, Benny Giovanni Cazarín-Santos, Verónica Marusa Borgonio-Cuadra, Pedro A. Reyes-López, José Manuel Rodríguez-Pérez

**Affiliations:** 1 Multidisciplinary Academic Division of Jalpa de Méndez, Universidad Juárez Autónoma de Tabasco, Jalpa de Méndez, Tabasco, Mexico; 2 Department of Molecular Biology, Instituto Nacional de Cardiología Ignacio Chávez, Mexico City, Mexico; 3 Multidisciplinary Academic Division of Comalcalco, Universidad Juárez Autónoma de Tabasco, Comalcalco, Tabasco, Mexico; 4 Academic Division of Health Sciences, Universidad Juárez Autónoma de Tabasco, Villahermosa, Tabasco, Mexico; 5 General Hospital de Yajalón Manuel Velasco Siles, Secretaría de Salud, Yajalón, Chiapas, Mexico; 6 Department of Endocrinology, Instituto Nacional de Cardiología Ignacio Chávez, Mexico City, Mexico; 7 Department of Genetics, Instituto Nacional de Rehabilitación Luis Guillermo Ibarra Ibarra, Mexico City, Mexico; 8 Division of Research, Instituto Nacional de Cardiología Ignacio Chávez, Mexico City, Mexico

**Keywords:** IL-6, inflammation, polymorphism, cardiovascular diseases, meta-analysis, genetic association

## Abstract

Cardiovascular diseases (CVD) are group of complex and multifactorial pathologies, in which interleukin-6 (*IL-6*) gene polymorphisms have been associated with several components of the CVD. Thus, in this study, we thoroughly reviewed and meta-analyzed evidence on the association between the *IL-6* (rs1800795) gene polymorphism and CVD. We systematically searched in the PubMed, Web of Sciences, and Scopus databases. The analyses were performed using five study groups based on (1) a combined pool of the overall populations, (2) the country of birth, (3) the continent of birth, (4) the diagnosis and (5) both location (country or continent) and diagnosis. The analysis included the allelic, homozygote, heterozygote, dominant and recessive models. The meta-analysis showed that -174*G>C* (rs1800795) is a risk factor for CVD (*allelic*: OR=1.06, CI 95%=1.02-1.10. Z p value <0.0001; homozygous: OR=1.11, CI 95%=1.03-1.19, Z p value= 0.002; *heterozygous*: OR=1.08, CI 95%=1.03-1.21, Z p value= 0.003; *dominant*: OR= 1.12, CI 95%= 1.07-1.18, Z p value= 0.001) and that this risk increases in the Chinese population. Additionally, we found that carriers of the *C* allele of 174*G>C* (rs1800795) polymorphism have an increase in the risk of coronary artery disease under the hereditary models assessed in the study. Using robust data, we found that *IL-6* (rs1800795) -174*G>C* gene polymorphism is associated with CVD risk.

## Introduction

Cardiovascular diseases (CVD) is define as the “pathological conditions involving the cardiovascular system including the heart; the blood vessels; or the pericardium” according to the MeSH (Medical Subject Headings) (https://www.ncbi.nlm.nih.gov/mesh). It is well known that inflammation plays a pivotal role in the development and progression of the CVD. Currently, one of the pro-inflammatory cytokines mainly studied is the interleukin-6 (IL-6) (Coker et al., 2011[14]; Banerjee et al., 2008[3]; Balding et al., 2004[2]; Bennet et al., 2003[7]; Akinyemi et al., 2017[1]).

IL-6 is a mediator of the inflammatory and immune responses and affects a variety of metabolic processes. In fact, it was proved in some prospective studies (Humphries et al., 2001[31], Jabir et al., 2017[33], Jenny et al., 2002[34]) that high basal plasmatic levels of IL-6 have a pro-inflammatory and procoagulant effect, which are risk factors for cardiovascular diseases.

Moreover, there is evidence that show a pro-inflammatory genetic profile associated with *IL-6 *polymorphisms suggesting that these genomic variants can be used as genetic marker in several diseases in which the underlying pathophysiology is strongly linked to an inflammatory process (Elsaid et al., 2014[17]; Chiappelli et al., 2005[13]; Flex et al., 2004[20]). Indeed, there are association studies that have addressed the pathophysiological contribution of the *IL-6* gene polymorphisms to CVD (Humphries et al., 2001[31], 2007[30]; Jabir et al., 2017[33]; Jenny et al., 2002[34]; Karahan et al., 2005[37]). The expression of *IL-6* is regulated mainly at the transcriptional level (Li et al., 2015[41]; Liaquat et al., 2014[42]). The promoter of the human *IL-6* gene contains several polymorphisms; one commonly studied variant is the single *G>C* base exchange polymorphism in the promoter region of *IL-6* gene, 174 base pairs (bp) upstream from the start site of transcription (-174*G>C*, rs1800795) (Karahan et al., 2005[37]; Li et al., 2015[41]; Kelberman et al., 2004[38]; Kou et al., 2017[39]; Lalouschek et al., 2006[40]). The -174*G>C* promoter polymorphism has been shown to be functionally important because it influences the transcription rate of the gene and the plasma concentrations of IL-6 (Satti et al., 2013[63]; Sekuri et al. 2007[64]; Sie et al., 2006[65]). Therefore, the selection of this genetic variant associated with IL-6 production is adequate to investigate the association with CVD (Wang et al., 2015[81]; Weger et al., 2005[82]; Yang et al., 2015[83]). 

Therefore, we aimed to perform a systematic review and a series of updated meta-analyses to evaluate the participation of -174*G>C* *IL-6* (rs1800795) gene polymorphism as a probable risk factor in coronary artery disease (CAD), ischemic stroke (IS), MI, and peripheral arterial occlusive disease (PAOD) due to the share underlying pathophysiology related to endothelial dysfunction and atherosclerosis (Theodorou and Boon, 2018[74]; Ismaeel et al., 2018[32]). We focused on all case-control studies of the association between -174*G>C* *IL-6* (rs1800795) and these diseases under allele, homozygote, heterozygote, dominant and recessive models. Based on the positive correlation observed, we explored the association by country and continent according to the models of inheritance.

The different diagnosis include CAD, IS, MI, and PAOD. We grouped results by CAD diagnosis to determine the presence of an association with -174*G>C* *IL-6 *(rs1800795). Finally, we explored the data by diagnosis and location. The specific objective of this analysis was to clarify the role of 174*G>C IL-6* (rs1800795) gene polymorphism in cardiovascular diseases.

## Materials and Methods

The systematic review protocol and data extraction for the meta-analysis was designed in accordance with the Preferred Reporting Items for Systematic reviews and Meta-Analysis (PRISMA). This study has been previously registered in PROSPERO (PROSPERO 2019 CRD42019125559).

### Eligible study search

We carried out an exhaustive electronic search in databases including PubMed, Web of Sciences and Scopus to identify studies that evaluated the role of *IL-6* gene polymorphisms as risk factors of cardiovascular diseases. The search algorithm used to recognize the eligible studies was as follows: (“*IL-6* gene” or “rs1800795” or “-174*G/C*”) and (“CVD” or “CHD” or “CAD” or “MI” or “cardiovascular disease” or “coronary artery disease” or “atherosclerosis” or “ischemic disease” or “myocardial infarction” or “stroke” or “peripheral arterial occlusive disease”). Furthermore, we conducted a manual search to retrieve pertinent articles cited in previous meta-analyses, systematic reviews, cohort and case-control studies, among others. 

### Selection criteria

We included full-length research studies that (1) addressed an independent association between *IL-6* gene polymorphisms and its role in patients with cardiovascular diseases, (2) included a case and comparison group design, (3) presented either clearly stated genotypes or sufficient information for estimation, (4) removed duplicate sample data, (5) were published in peer-reviewed journals, and (6) were written in English.

### Data extraction

The following information was independently extracted in each study by four investigators, while a fifth researcher verified and solved any discrepancies in the following categories: the surname of the first author, publication year, country of origin, ethnicity, diagnosis of cases and source of controls, inclusion/exclusion criteria of cases and controls, number of cases and controls, and case and control genotype frequencies. When the studies included subjects of more than one ethnicity or diagnosis type, the genotype data were extracted separately. 

### Quality assessment

The quality of the studies included in the analysis was assessed separately by two researchers using the Newcastle-Ottawa Scales (NOS); these scales are based on three main aspects: selection, comparability and ascertainment of exposure. Only studies with a score of six stars or more were included in the meta-analysis (http://www.ohri.ca/programs/clinical_epidemiology/oxford.asp). 

### Statistical analysis

Firstly, using a chi-squared test, we tested the Hardy-Weinberg equilibrium (HWE) for genotype frequencies in cases and controls, where *P*<0.05 was considered statistically significant. Statistical analyses were performed considering the following categories: a) carrier with disease, b) carrier without disease, c) non-carrier with disease, d) non-carrier without disease, the term “carrier” refers to the allele *C* of *IL-6* (rs1800795 or -174*G>C*). Then, the relation between *IL-6* (rs1800795 or -174*G/C*) polymorphism and CVD was addressed by the pooled ORs and their corresponding 95% confidence intervals under five genetic models, namely the allelic model (*C* vs *G*), the dominant model (*CC*+*GC* vs *GG*), the recessive model (*CC* vs *CG*+*GG*), the homozygous model (*CC* vs *GG*) and the co-dominant model (*GC* vs *GG*). To assess the significance of the pooled ORs, we used a Z test and considered a *P* <0.05 as statistically significant. For the meta-analysis, a total of 16 groups were created based on five categories: (1) combined from the overall population, (2) based on the country of birth (China, Turkey, India and United Kingdom), (3) based on the continent of birth (Europeans and Africans), (4) dependent on diagnosis (CAD, IS, MI, PAOD, and healthy subjects as controls), and (5) based on both the diagnosis and the country or continent of origin (India + CAD, Europe + CAD, Europe + MI, and Europe + IS) and (6) based on smoking habits.

In addition, the heterogeneity between the studies was analyzed by a Q-statistic test and the inconsistency was evaluated by an I^2^ statistic. The I^2^ results were (a) 0-25 absent, (b) 25-50 low, (c) 50-75 moderate, and (d) 75-100 high.

Alternatively, I^2^ >50 % and Q test *P* value ≤ 0.1 were taken as indicators of substantial heterogeneity, in which case, the effect model we used was random-effects (DerSimonian-Laird method), failing that, the fixed effect model (Mantel-Haenszel method). 

The sensitivity analysis was conducted by sequentially omitting one article to evaluate the influence of an individual study and validate the reliability of the results. Furthermore, the publication bias was diagnosed with Begg's funnel plot and Egger's regression test; *P* <0.05 was considered as a significant publication bias. The comprehensive meta-analysis software version 2 (Biostat, Englewood, NJ/USA) was used for all the analyses, and all p-values were two-tailed. 

## Results

### Characteristics of the eligible studies

**Figure 1:**
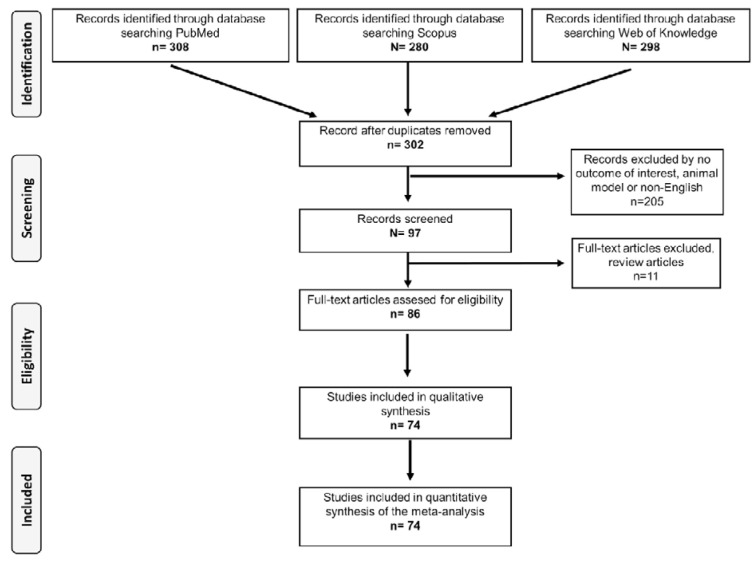
Flow-chart diagram to selected studies in the meta-analysis

After excluding the overlapping articles of the literature search and applying the selection criteria mentioned previously, 74 research articles (Coker et al., 2011[14]; Banerjee et al., 2008[3], 2009[4]; Balding et al., 2004[2]; Bennet et al., 2003[7]; Akinyemi et al., 2017[1]; Humphries et al., 2001[31], 2007[30]; Jabir et al., 2017[33]; Jenny et al., 2002[34]; Elsaid et al., 2014[17]; Chiappelli et al., 2005[13]; Flex et al., 2004[20]; Karahan et al., 2005[37]; Li et al., 2015[41]; Liaquat et al., 2014[42]; Kelberman et al., 2004[38]; Kou et al., 2017[39]; Lalouschek et al., 2006[40]; Satti et al., 2013[63]; Sekuri et al. 2007[64]; Sie et al., 2006[65]; Wang et al., 2015;[81] Weger et al., 2005[82]; Yang et al., 2015[83]; Basso et al., 2002[5]; Bennermo et al., 2011[6]; Berg et al., 2009[8]; Bhanushali et al., 2013[9]; Buraczynska et al., 2016[10]; Chakraborty et al., 2013[11]; Chamorro et al., 2005[12]; Danielsson et al., 2005[15]; Densem et al., 2005[16]; Fan et al., 2011[18]; Flex et al., 2002[21]; Galimudi et al., 2014[22]; George et al., 2004[23]; Georges et al., 2001[24]; Ghazouani et al., 2010[26], 2011[25]; Greisenegger et al., 2003[27]; Hongmei et al., 2016[28]; Jun et al., 2017[36]; Licastro et al., 2004[43]; Lieb et al., 2004[44]; Maitra et al., 2008[48]; Mao et al., 2016[49]; Mastana et al., 2017[50]; Mishra et al., 2013[51]; Mitrokhin et al., 2017[52]; Myśliwska et al., 2006[53]; Nauck et al., 2002[54]; Panoulas et al., 2009[55]; Phulukdaree et al., 2013[56]; Potaczek et al., 2007[57]; Revilla et al., 2002[58]; Rios et al., 2010[59]; Rosner et al., 2005[60]; Salama and Hammad, 2015[61]; Sarecka et al., 2008[62]; Silander et al., 2008[66]; Smallwood et al., 2008[67]; Smith et al., 2008[68]; Spoto et al., 2015[69]; Stephens et al., 2004[70]; Sun et al., 2014[72]; Tong et al., 2010[75], 2013[76]; Tretjakovs et al., 2007[77]; Tuttolomondo et al., 2012[78]; Tütün et al., 2006[79]; Vakili et al. 2011[80]) were selected for the analysis. The flow diagram in Figure 1(Fig. 1) shows the steps of the study selection process.

Moreover, these 74 papers that included a total of 33,525 cases and 52,704 controls. In Table 1(Tab. 1) (References in Table 1: Akinyemi, 2017[1]; Balding, 2004[2]; Banerjee, 2008[3]; Basso, 2002[5]; Bennermo, 2011[6]; Bennet, 2003[7]; Berg, 2009[8]; Bhanushali, 2013[9]; Buraczynska, 2016[10]; Chakraborty, 2013[11]; Chamorro, 2005[12]; Chiapelli, 2005[13]; Coker, 2011[14]; Danielsson, 2005[15]; Densem, 2005[16]; Elsaid, 2014[17]; Fan, 2011[18]; Flex, 2002[21]; Flex, 2004[20]; Flex, 2007[19]; Galimudi, 2014[22]; George, 2004[23]; Georges, 2001[24]; Ghazouani, 2010[26]; Ghazouani, 2011[25]; Greisenegger, 2003[27]; Hongmei, 2016[28]; Humphries, 2001[31]; Humphries, 2007[30]; Jabir, 2017[33]; Jenny, 2002[34]; Jun, 2017[36]; Karahan, 2005[37]; Kelberman, 2004[38]; Kou, 2017[39]; Lalouschek, 2006[40]; Li, 2015[41]; Liaquat, 2014[42]; Licastro, 2004[43]; Lieb, 2004[44]; Maitra, 2008[48]; Mao, 2016[49]; Mastana, 2017[50]; Mishra[51], 2013; Mitrokhin, 2017[52]; Mysliwska, 2006[53]; Nauck, 2002[54]; Panoulas, 2009[55]; Phulukdaree, 2013[56]; Potacsek, 2007[57]; Revilla, 2002[58]; Rios, 2010[59]; Rosner, 2005[60]; Salama, 2015[61]; Sarecka, 2008[62]; Satti, 2013[63]; Sekuri, 2007[64]; Sie, 2006[65]; Silander, 2008[66]; Smallwood, 2008[67]; Smith, 2008[68]; Spoto, 2015[69]; Stephens, 2004[70]; Sun, 2014[72]; Tong, 2010[75]; Tong, 2013[76]; Tretjakovs, 2007[77]; Tuttolomondo, 2012[78]; Tütün 2006[79]; Vakili, 2011[80]; Wang, 2015[81]; Weger, 2005[82]; Yang, 2015[83]) are shown the genotypic frequencies in cases and controls of both the HWE analysis and all included studies. These articles addressed the relation of the aforementioned diseases to the rs1800795 polymorphism; however, some articles displayed the genotype frequencies for the sample origin (France, Ireland, among others) (Georges et al., 2001[24]; Rios et al., 2010[59]) or the detailed diagnoses (MI, IS, CAD, PAOD) (Banerjee et al., 2008[3]; Jenny et al., 2002[34]; Sie et al., 2006[65]; Nauck et al., 2002[54]; Silander et al., 2008[66]), for this reason, the frequencies were described separately. As a result, the meta-analysis distribution of the 74 articles was based on the country (China= 11, Turkey= 4, India= 8 and, United Kingdom= 9), continent (Europeans= 37 and Africans= 3) and sample diagnosis (CAD= 27, IS= 10, MI= 13, PAOD= 4 and healthy controls= 53). Moreover, other sub-groups were integrated by the combination of two filters: (a) sample born in India and cases diagnosed with CAD (India + CAD: 6), (b) sample born in Europe and cases diagnosed with CAD (Europe + CAD=7), (c) sample born in Europe and cases diagnosed with MI (Europe + MI=9), and (d) sample born in Europe and cases diagnosed with IS (Europe + IS=5). The quality of the studies was evaluated based on the NOS assessment (Supplementary Table 1).

### Role of rs1800795 in CVD in the overall population

We evaluated the participation of -174*G>C* (rs1800795) as a probable risk factor for CVD. The findings reveal a statistical association of this polymorphic variant in four of the five models proposed previously (*allelic*: OR=1.06, CI 95%=1.02-1.10, Z p value <0.0001; *homozygous*: OR=1.11, CI 95%=1.03-1.19, Z p value= 0.002; *heterozygous*: OR=1.08, CI 95%=1.03-1.21, Z p value= 0.003; *dominant*: OR= 1.12, CI 95%= 1.07-1.18, Z p value= 0.001; (Supplementary Table 2).

Regarding the *recessive* model, a statistical association was only observed in the presence of heterogeneity (OR= 1.18, CI 95%= 1.04-1.34, Z p value= 0.008, I^2^=77.64). There was no evidence of publication bias in the five genetics models proposed, all models are shown in the Supplementary Figures.

### Role of rs1800795 in CVD by country of birth

**Figure 2:**
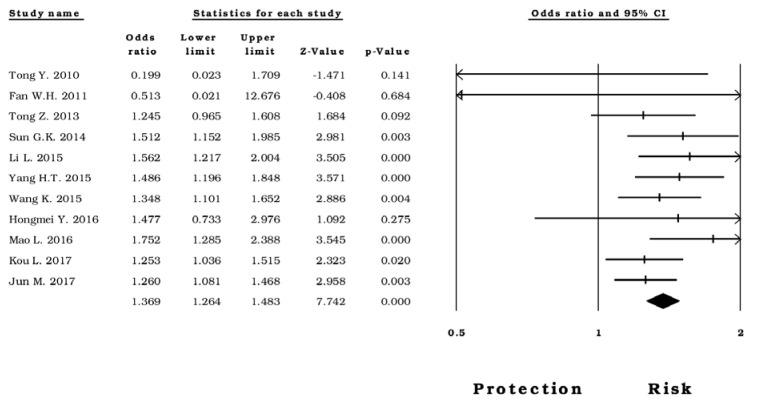
Forest plot of the* allelic* model in subjects born in China

In this analysis, we performed a meta-analysis in four different countries: China, India Turkey, and the United Kingdom. Firstly, in the Chinese population, there was no evidence of heterogeneity in the genetic models and rs1800795 was a risk factor due to its association with CVD (*allelic* OR=1.36, CI 95%=1.26-1.48, Z p value= <0.0001 (Figure 2(Fig. 2); References in Figure 2: Fan, 2011[18]; Hongmei, 2016[28]; Jun, 2017[36]; Kou, 2017[39]; Li, 2015[41]; Mao, 2016[49]; Sun, 2014[72]; Tong, 2010[75]; Tong, 2013[76]; Wang, 2015[81]; Yang, 2015[83]); *homozygous *OR*=* 1.91, CI 95%=1.61-2.27, Z p value= <0.0001; *heterozygous* OR=1.21, CI 95%=1.09-1.34, Z p value= <0.0001; *dominant* OR= 1.16, CI 95%=1.05-1.27, Z p value= 0.002; *recessive* OR= 1.78, CI 95%= 1.51-2.10, Z p value <0.0001).

**Figure 3:**
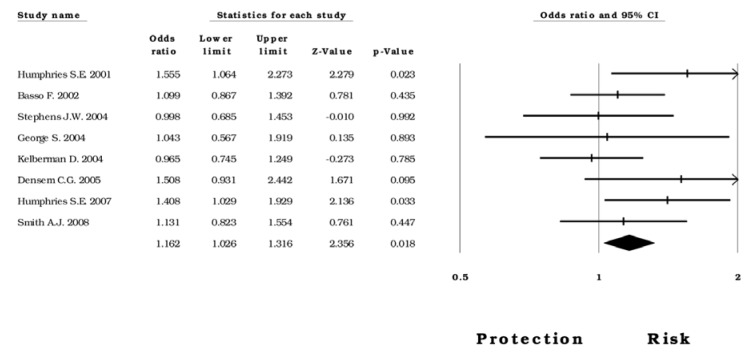
Forest plot of the heterozygous model in subjects born in the United Kingdom

Secondly, we evaluated the polymorphism involvement in a sample population from the United Kingdom; the result shows a significant association with risk under the *heterozygous* model (OR=1.16; CI 95%= 1.02-1.31; Z p value 0.018); (Figure 3(Fig. 3); References in Figure 3: Basso, 2002[5]; Densem, 2005[16]; George, 2004[23]; Humphries, 2001[31]; Humphries, 2007[30]; Kelberman, 2004[38]; Smith, 2008[68]; Stephens, 2004[70]). Furthermore, the *dominant* model revealed an association (OR= 1.15, CI 95%= 1.00-.131, Z p value 0.039, I^2^=29.28) in the presence of moderate heterogeneity, this was not maintained in its absence of heterogeneity. As for the articles with Turkish and Indian samples, the same five genetic models were carried out; however, the data did not show an association of -174*G>C* variant as a probable risk factor in those populations.

The Egger test was not statistically significant in the five genetic models performed, thus suggesting the absence of publication bias.

### Role of rs1800795 in CVD by continent of birth

**Figure 4:**
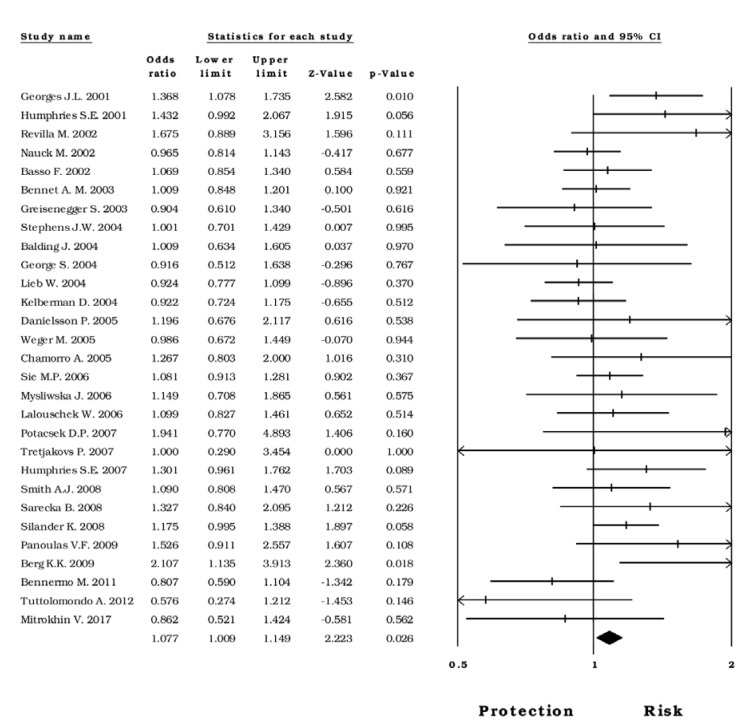
Forest plot of the *dominant* model in subjects born in Europe

For this analysis, we divided the sample into two groups the first of European and the second of African participants. As for the first group, the findings indicated that -174*G>C* variant is a significant risk factor for CVD under the *dominant* model (OR=1.07; CI 95%= 1.00-1.14; Z p value = 0.026); (Figure 4(Fig. 4); References in Figure 4: Balding, 2004[2]; Basso, 2002[5]; Bennermo, 2011[6]; Bennet, 2003[7]; Berg, 2009[8]; Chamorro, 2005[12]; Danielsson, 2005[15]; George, 2004[23]; Georges, 2001[24]; Greisenegger, 2003[27]; Humphries, 2001[31]; Humphries, 2007[30]; Kelberman, 2004[38]; Lalouschek, 2006[40]; Lieb, 2004[44]; Mitrokhin, 2017[52]; Mysliwska, 2006[53]; Nauck, 2002[54]; Panoulas, 2009[55]; Potacsek, 2007[57]; Revilla, 2002[58]; Sarecka, 2008[62]; Sie, 2006[65]; Silander, 2008[66]; Smith, 2008[68]; Stephens, 2004[70]; Tretjakovs, 2007[77]; Tuttolomondo, 2012[78]; Weger, 2005[82]).

Moreover, under the *heterozygous* model (OR=1.18; CI 95%= 1.02-1.36; Z p value = 0.022; I^2^=83.47), also reveals an association of this variant with CVD in the presence of high heterogeneity. Nevertheless, after excluding the studies that predispose to heterogeneity, this association was not observed. Regarding the African population, no statistical association was observed under any genetic model.

Begg's funnel or Egger's test did no present asymmetry or statistical significance, thus suggesting the absence of publication bias in the genetic models analyzed (Supplementary Figures).

### Role of rs1800795 in CVD by clinical diagnosis

**Figure 5:**
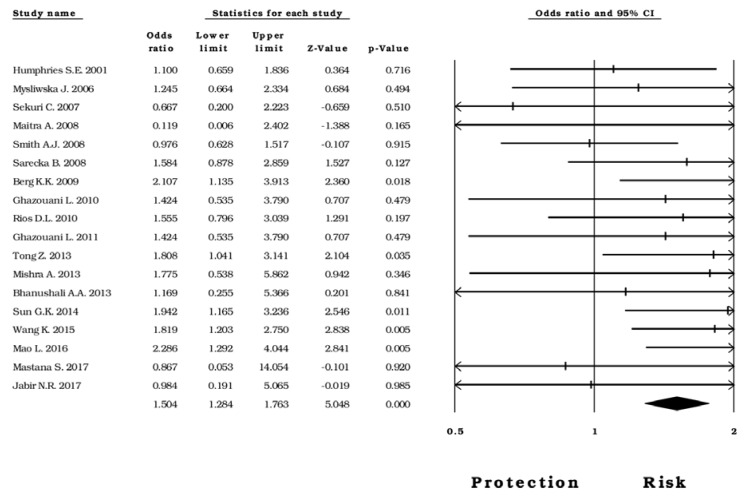
Forest plot of the *homozygous* model in subjects diagnosed with CAD

**Figure 6:**
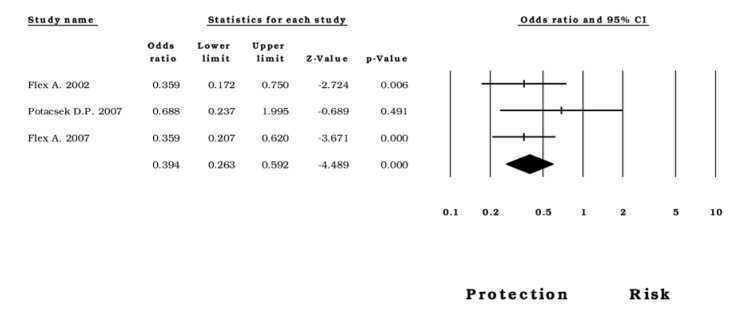
Forest plot of the *recessive* model in subjects diagnosed with PAOD

The available data allowed the creation of five analysis groups with subjects diagnosed with CAD, IS, MI, PAOD, as well as with healthy subjects as controls. First, we evaluated the risk of -174*G>C* for CAD; under the five models used, a significant association was observed (*allelic*: OR=1.14, CI 95%=1.04-1.23, Z p value 0.002; *homozygous*: OR=1.50, CI 95%=1.28-1.76, Z p value <0.0001; Figure 5(Fig. 5); References in Figure 5: Berg, 2009[8]; Bhanushali, 2013[9]; Ghazouani, 2010[26]; Ghazouani, 2011[25]; Humphries, 2001[31]; Jabir, 2017[33]; Maitra, 2008[48]; Mao, 2016[49]; Mastana, 2017[50]; Mishra, 2013[51]; Mysliwska, 2006[53]; Rios, 2010[59]; Sarecka, 2008[62]; Sekuri, 2007[64]; Smith, 2008[68]; Sun, 2014[72]; Tong, 2013[76]; Wang, 2015[81]; *heterozygous*: OR=1.10, CI 95%=1.02-1.19, Z p value= 0.013; *dominant*: OR= 1.23, CI 95%= 1.11-1.35, Z p value <0.0001; *recessive* OR= 1.31, CI 95%= 1.10-1.56, Z p value =0.002). Then, we evaluated this association in subjects with PAOD and found a protective effect of the -174*G>C* polymorphism under the *recessive* model (OR=0.39, CI 95%=0.26-0.59, Z p value <0.0001; Figure 6(Fig. 6); References in Figure 6: Flex, 2002[21]; Flex, 2007[19]; Potacsek, 2007[57]). Regarding the cases diagnosed with IS and MI, the data did not show any statistical relation with the rs1800795 genomic variant (Supplementary Table 3). 

**Figure 7:**
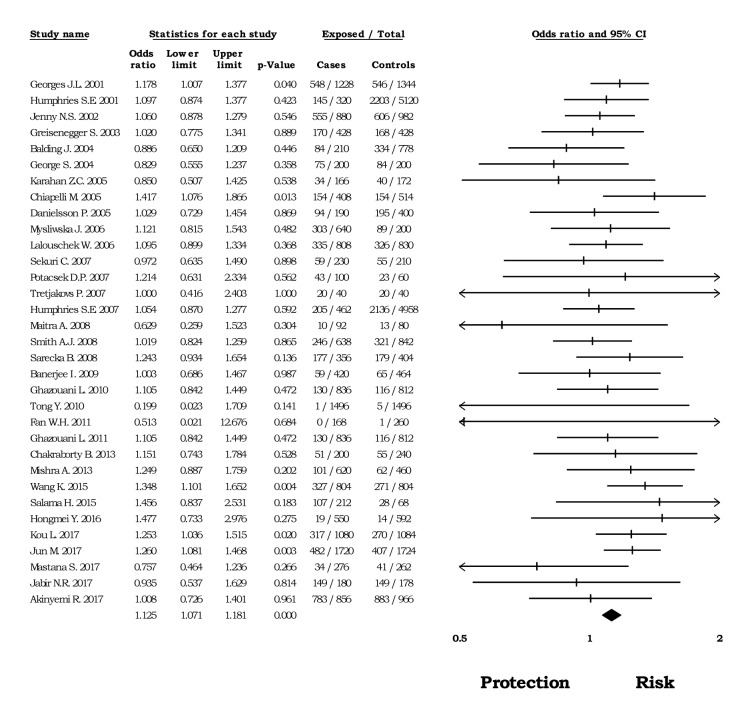
Funnel plot of the *allelic* model in healthy subjects as controls

In some articles, a hospital population was included in the control group; herein, only healthy subjects were used as controls. The results of this analysis revealed a role of -174*G>C* polymorphism as a risk factor for CVD under the *allelic* (OR= 1.12, CI 95%= 1.07-1.18, Z p value <0.0001; Figure 7(Fig. 7); References in Figure 7: Akinyemi, 2017[1]; Balding, 2004[2]; Banerjee, 2009[4]; Chakraborty, 2013[11]; Chiapelli, 2005[13]; Danielsson, 2005[15]; Fan, 2011[18]; George, 2004[23]; Georges, 2001[24]; Ghazouani, 2010[26]; Ghazouani, 2011[25]; Greisenegger, 2003[27]; Hongmei, 2016[28]; Humphries, 2001[31]; Humphries, 2007[30]; Jabir, 2017[33]; Jenny, 2002[34]; Jun, 2017[36]; Karahan, 2005[37]; Kou, 2017[39]; Lalouschek, 2006[40]; Maitra, 2008[48]; Mastana, 2017[50]; Mishra, 2013[51]; Mysliwska, 2006[53]; Potacsek, 2007[57]; Salama, 2015[61]; Sarecka, 2008[62]; Sekuri, 2007[64]; Smith, 2008[68]; Tong, 2010[75]; Tretjakovs, 2007[77]; Wang, 2015[81]), *homozygous* (OR= 1.23, CI 95%= 1.11 1.37, Z p value <0.0001), *heterozygous* (OR= 1.17, CI 95%= 1.10-1.24, Z p value <0.0001), and dominant models (OR= 1.24, CI 95%= 1.16-1.31, Z p value <0.0001). Nonetheless, in the presence of heterogeneity, an association was found under the recessive model (OR= 1.31, CI 95%= 1.11-1.54, Z p value <0.0001; I^2^=76.56), but not when the articles favoring heterogeneity were excluded. We did not find publication bias using Egger's test in the genetics models previously mentioned (Supplementary Figures).

### Role of rs1800795 in CVD by diagnosis and geographical location

Finally, we conducted an analysis with two filter criteria: diagnosis (CAD, MI, or IS) and geographical location (India or Europe), which allowed for the formation of four groups: India + CAD, Europe + CAD, Europe + MI and Europe + IS (Table 2(Tab. 2)). Using five genetic models with or without heterogeneity, we did not observe a statistically significant participation of -174*G>C* as a possible marker. We did not find publication bias using Egger's test in the five genetic models conducted (Supplementary Figures).

### Role of rs1800795 in CVD by smoking habits

Finally, due to the importance that could have some risk factors in CVD we performed an analysis by the smoking habits. The articles with available data showing the genotype distribution by tobacco used were only six articles (Humphries et al., 2001[31]; Greisenegger et al., 2003[27]; Balding et al., 2004[2]; Sie et al., 2006[65]; Mysliwska et al., 2006[53]; Mishra et al., 2013[51]). However, even we discarded the studies that were favoring the heterogeneity no evidence of association was found (allelic: OR=1.34, CI 95%=0.94-1.92, Z p value 0.103; homozygous: OR=1.01, CI 95%=0.88-1.16, Z p value 0.853; heterozygous: OR=1.08, CI 95%=0.70-1.67, Z p value= 0.724; dominant: OR= 1.28, CI 95%= 0.85-1.91, Z p value 0.226; recessive OR= 1.18, CI 95%= 0.73-1.91, Z p value =0.480) (Supplementary Table 4). Egger's test did not reveal publication bias.

## Discussion

It is well known that inflammatory mediators, especially IL-6, are central to the development of cardiovascular diseases. A considerable number of the polymorphisms were identified in the *IL-6* gene, especially inside the non-coding promoter sequence. It has been reported that these polymorphisms exert a powerful influence on the expression of this gene. Hence, we evaluated the participation of the -174*G>C* (rs1800795) *IL-6* gene polymorphism as a probable risk factor for cardiovascular diseases. First, we explored the participation of -174*G>C* (rs1800795) polymorphism as a possible risk factor for CVD in the overall population of the included studies.

After evaluating heterogeneity, we found that this polymorphism increased the risk for CVD under the allelic (*C*), homozygous (*CC*), heterozygous (*CG*) and dominant (*CC* + *CG*) models. In accordance with the present results, previous studies have demonstrated that higher levels of IL-6 are associated with the -174*CC* genotype or *C* allele in patients (Panoulas et al., 2009[55]; Liu et al., 2006[46]; Stoica et al., 2010[71]). Taken together, these results suggest that when the -174*C* is present, patients exhibit higher levels of IL-6.

This shows the influence of the polymorphism in increasing *IL-6* gene transcription and predisposing to greater myocardial or vascular injury. Several studies and the large sample size included in this meta-analysis provided more reliable information related to the association of *IL-6* (rs1800795) gene polymorphism and CVD. 

Additionally, previous studies have suggested that there could be differences in gene frequencies between populations (Humphries et al., 2007[30]; Ghazouani et al., 2010[26]; Greisenegger et al., 2003[27]; Hongmei et al., 2016[28]). Thus, our objective was to explore the involvement of -174*G>C* in CVD by performing a sub-analysis on different nationalities and geographic locations. The results showed that in the studies within the Chinese population, there was a strong association of the *IL-6* (rs1800795) polymorphism with CVD.

Indeed, depending on the model, *C* allele carriers developed an increased risk (1.16 to 1.91 fold) of having CVD. Our results suggest that the risk in the Chinese population is higher than in other populations analyzed in this study. We analyzed the same association with subjects born in the United Kingdom, Turkey or India. Under a heterozygous model, we only found a significant association for the British population after discarding the heterogeneity.

**Figure 8:**
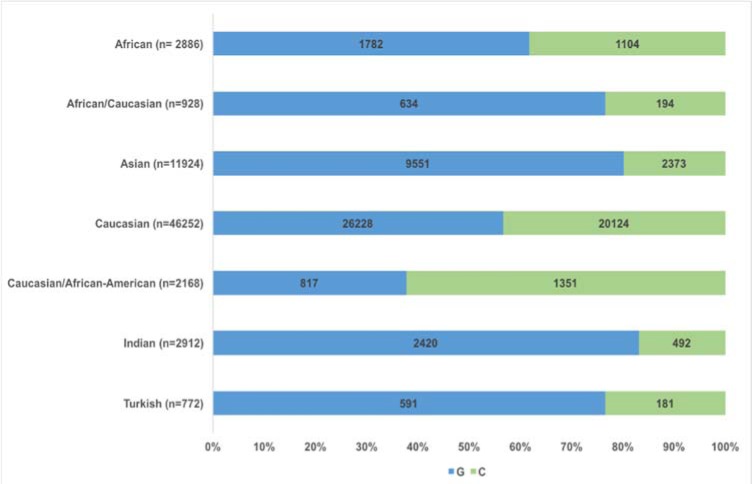
Allele frequencies of cases by population

Additionally, we made a diagram of the allelic frequencies of the cases by populations, in which the distribution of the risk allele is observed (Figure 8(Fig. 8)). 

A possible explanation for this might be that, in different populations, the underlying genetic mechanisms that predispose to the same pathology may be achieved by different genotypes affecting distinct mediating mechanisms. Therefore, the influence of the population genomes needs to be taken into account when considering the effect of -174*G>C*, especially in complex and multifactorial diseases such as CVD. Afterward, we decided to explore this issue more precisely with the following analysis. Depending on the sample nationality, the available data allowed for the formation of two groups (Europeans and Africans), for which the same methodological procedure was used. Under a dominant model (OR = 1.07, CI 95% = 1.00-1.14, Z p value = 0.026), only one statistically significant association was observed after measuring heterogeneity in the European population.

By taking the previous analysis into consideration, we can suggest that this association might be influenced by the studies of participants born in the United Kingdom. These findings confirmed the assumption that ethnicity increases the level of complexity of genetic functional studies, considering the differences in gene frequencies between populations (Satti et al., 2013[63]; Sekuri et al., 2007[64]; Sie et al., 2006[65]).

Consequently, the consideration of ancestral components of disease may become more relevant to understand inherited cardiovascular risk (Wang et al., 2015[81]; Tütün et al., 2006[79]; Vakili et al. 2011[80]).

An initial objective of the project was to better understand the influence of both the genes and the specific DNA sequence variants responsible for the etiology of cardiovascular diseases. For this reason, we evaluated the role of -174*G>C* through the specific diagnosis of the patient groups with CAD, PAOD, MI, and IS; only healthy subjects were chosen as healthy controls (HC).

Importantly, we found that this polymorphism is associated with CAD. In fact, under the genetic models used, carriers of -174*G>C* have an increased risk for CAD between 1.10 times and 1.50 times. It is well known that high basal IL-6 plasma levels, which exert pro-inflammatory and pro-coagulant effects, have proven to be predictive of CVDs (Wang et al., 2015[81]; Tuttolomondo et al., 2012[78]; Tütün et al., 2006[79]; Vakili et al. 2011[80]). Our results confirm the risk effect of *C* carriers of -174*G>C* on CAD. In fact, this finding broadly supports the work of Phulukdaree et al., who observed that the presence of the IL-6 -174*G>C* the *C* allele influences the levels of IL-6 and increases the risk of CAD in South African Indians (Phulukdaree et al., 2013[56]). Taken together, these results further support the use of *IL-6* gene polymorphism 174*G>C*, and IL-6 levels as CAD genetic marker.

On the other hand, we were aware that there could be other variables affecting the results. For that reason, a more selective analysis was performed which only included healthy subjects as a comparison group. We found a 1.02 to 1.25 fold increased risk of cardiovascular diseases, supports our previous association of -174*C* carriers with CVD.

In addition, in the PAOD analysis, the recessive model *C* allele of -174*G>C* is associated with protection [OR = 0.39, CI 95%= (0.26-0.59), Z p value <0.0001]. In fact, Flex et al. reported that *GG* homozygous subjects have a 4.6-fold risk of developing PAOD compared with *CC* homozygous patients (Flex et al., 2002[21]); this result thus reinforced the idea that the *C* allele could confer a protective effect. However, the analysis performed in MI and IS patients did not reveal any association. This discrepancy could be explained in part by the *in vitro* observations of Terry et al., who reported that IL-6 expression is regulated differently in various cells (Terry et al., 2000[73]). Consequently, the levels of this interleukin may be dependent on the gene expression of a particular cell type and its associated phenotype. 

Additionally, we know that the effect of -174*G>C* on circulating IL-6 is more complex and may be dependent on multiple variables. Hence, our final approach involved examining the role of -174*G>C* in not only Europeans with CAD, MI, and IS, but also in India participants with CAD. The aforementioned subjects were organized into four groups: Europe + CAD, Europe + MI, Europe + IS and India + CAD. Of interest, even after the heterogeneity was discarded in the analyses, no association with -174*G>C* polymorphism was found. 

Furthermore, it is well known that there are several risk factors involved in the CVDs. One of the most common studied is the smoking habits, which it has been hypothesized that could play a role as risk factor. Nevertheless, in our findings no relationship was revealed. However, this could be an effect of a small sample size, due to only six studies the data was available to perfom the analysis. Another reason could be that almost all of the studies included are conducted in Caucasians (Humphries et al., 2001[31]; Greisenegger et al., 2003[27]; Balding et al., 2004[2]; Sie et al., 2006[65]; Mysliwska et al., 2006[53]) and it is possible that other risk factors could be interfered in this type population.

Also, previous studies had already performed some of the analysis made in our article (Ma et al., 2011[47]; Zheng et al., 2012[87]; Yin et al., 2012[85], 2013[86]; Yang et al., 2013[84]; Jin et al., 2014[35]; Hou et al., 2015[29]; Liu et al., 2015[45]) however, these previous reports failed to take into consideration the following aspects. First, while previous works only analyzed one or two sub-groups, the number of analyses performed here (total groups: China, United Kingdom, Turkey, India, Europeans, Africans, CAD, PAOD, MI, IS; HC, India +CAD, Europe + CAD, Europe + MI and Europe + IS) include 16 sub-groups that evaluated the influence of the ethnicity, diagnosis, geographical localization, or a combination of them. Second, our sample size is larger; while the sample in previous studies contained 6 to 48 articles, we included 74 articles in this meta-analysis and 85 in the systematic review. Lastly, while some of the previous studies included data from master's or doctoral theses, our meta-analysis sample consisted of only articles published in peer-reviewed journals. 

The interpretation of the meta-analysis results is subject to certain limitations. First, we need to consider the sample size. Although the total number of study subjects was 33,525 cases and 52,704 controls, in some sub-analysis groups, such as PAOD or India, the article sample size could be considered small. This could have an effect in the outcomes. Nevertheless, there were 16 sub-analyses performed in this work; the sheer quantity should be considered a strength because it allows for a general panorama of the effect of -174*G>C* in CVD. Second, we performed an analysis to evaluate the publication bias but found that it could not be discarded, because most of the articles were from either Europe or Asia. Further research is, therefore, an essential next step to provide more definitive evidence. Third, the effect of the -174*G>C* polymorphism is complex and depends on the presence of age, BMI, and other clinical characteristics, which were not evaluated in this meta-analysis. 

However, a detailed systematic review was indeed performed to explore these characteristics in the included articles. Fourth, removing non-English literature and articles without related data from the analysis might affect results. Nevertheless, our inclusion and exclusion criteria allowed for the inclusion of quality studies. Finally, this meta-analysis did not take into consideration the possibility of linkage disequilibrium between polymorphisms, as well as gene-gene or gene-environment interactions.

In this study several characteristics were considered that can influence the role of -174*G/C* as a risk factor for CVD, such as the clinical situation of the control group. 

In conclusion our results indicate that *C* allele of *IL-6* gene polymorphism (rs1800795) is associated with increased risk for CVD.

This association is mainly observed in Chinese and British populations and patients with CAD.

However, the -174*G>C* polymorphism was also found to be a protective factor for PAOD. Further research is needed to fully understand the participation of the -174*G>C* variant in CVD.

## Notes

Thelma Beatriz González-Castro and Yazmín Hernández-Díaz contributed equally as first authors.

## Acknowledgements

The authors are grateful to all study participants and to the Department of Molecular Biology, Instituto Nacional de Cardiología Ignacio Chávez, Mexico City, Mexico and Universidad Juárez Autónoma de Tabasco, Tabasco, Mexico.

## Supplementary Material

Supplementary data

## Figures and Tables

**Table 1:**
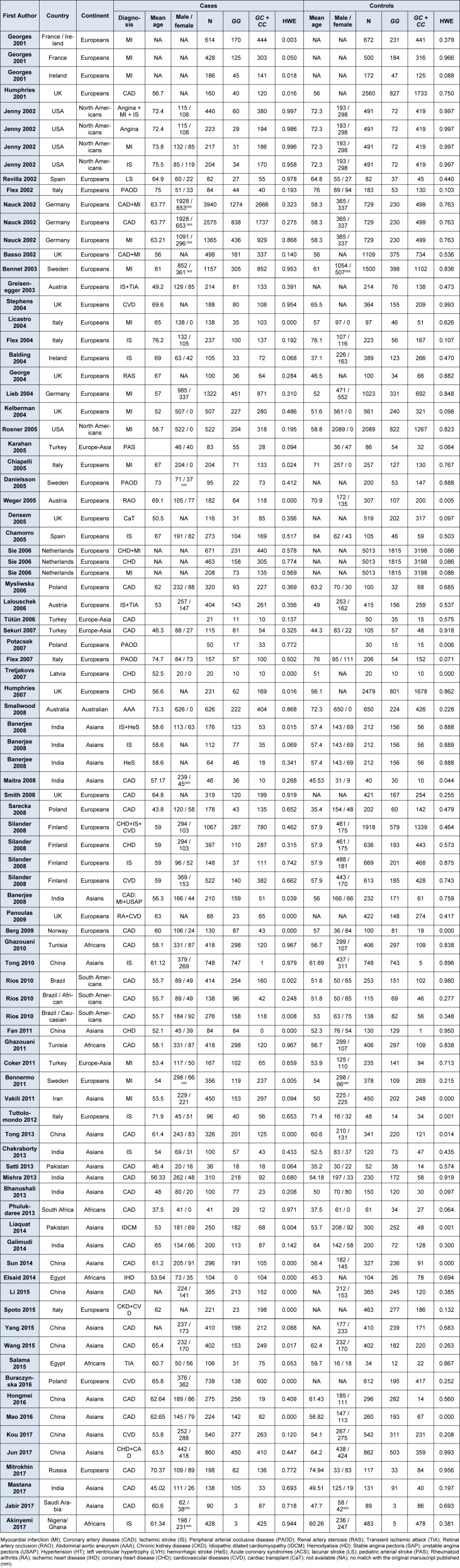
Genotypic distribution of the studies included in the systematic review and meta-analysis

**Table 2:**
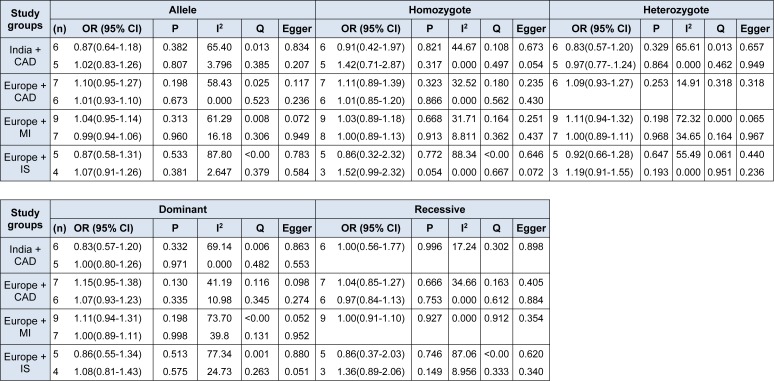
Meta-analysis of the association between rs1800795 polymorphism with clinical diagnosis and ethnicities
