# Supplementary material for: Interleukin 6 (rs1800795) gene polymorphism is associated with cardiovascular diseases: a meta-analysis of 74 studies with 86,229 subjects
Source: EXCLI J. 2019 Jun 7;18:331–55. doi: 10.17179/excli2019-1248 (PMC6635721; doi:10.17179/excli2019-1248)
Supplement: Supplementary data [file EXCLI-18-331-s-001.pdf]

EXCLI Journal 2019;18:331-355 – ISSN 1611-2156
Received: March 06, 2019, accepted: April 09, 2019, published: June 07, 2019

Supplementary data to:

INTERLEUKIN 6 (RS1800795) GENE POLYMORPHISM IS ASSOCIATED WITH CARDIOVASCULAR DISEASES: A META-ANALYSIS OF 74 STUDIES WITH 86,229 SUBJECTS

Thelma Beatriz González-Castro[1#], Yazmín Hernández-Díaz[1#], Nonanzit Pérez-Hernández[2], Carlos Alfonso Tovilla-Zárate[3], Isela Esther Juárez-Rojop[4], María Lilia López-Narvaez[5], Ruben Blachman-Braun[2], Rosalinda Posadas-Sánchez[6], Gilberto Vargas-Alarcón[2], Esbeidy García-Flores[2], Benny Giovanni Cazarín-Santos[2], Verónica Marusa Borgonio-Cuadra[7], Pedro A. Reyes-López[8], José Manuel Rodríguez-Pérez[2*]

1 Multidisciplinary Academic Division of Jalpa de Méndez, Universidad Juárez Autónoma de Tabasco, Jalpa de Méndez, Tabasco, Mexico
2 Department of Molecular Biology, Instituto Nacional de Cardiología Ignacio Chávez, Mexico City, Mexico
3 Multidisciplinary Academic Division of Comalcalco, Universidad Juárez Autónoma de Tabasco, Comalcalco, Tabasco, Mexico
4 Academic Division of Health Sciences, Universidad Juárez Autónoma de Tabasco, Villahermosa, Tabasco, Mexico
5 General Hospital de Yajalón Manuel Velasco Siles, Secretaría de Salud, Yajalón, Chiapas, Mexico
6 Department of Endocrinology, Instituto Nacional de Cardiología Ignacio Chávez, Mexico City, Mexico
7 Department of Genetics, Instituto Nacional de Rehabilitación Luis Guillermo Ibarra Ibarra, Mexico City, Mexico
8 Division of Research, Instituto Nacional de Cardiología Ignacio Chávez, Mexico City, Mexico

# These authors contributed equally to this work.

* Corresponding author: José Manuel Rodríguez Pérez, Ph.D. Department of Molecular Biology, Instituto Nacional de Cardiología Ignacio Chávez. Juan Badiano No. 1, Col Sección XVI, Tlalpan 14080, Mexico City, Mexico. Tel: (+52-55) 55732911, Ext: 26301, E-mail: josemanuel_rodriguezperez@yahoo.com.mx

http://dx.doi.org/10.17179/excli2019-1248

This is an Open Access article distributed under the terms of the Creative Commons Attribution License (http://creativecommons.org/licenses/by/4.0/).

EXCLI Journal 2019;18:331-355 – ISSN 1611-2156
Received: March 06, 2019, accepted: April 09, 2019, published: June 07, 2019

Supplementary Table 1: Descriptive characteristics of the studies included in the systematic review and meta-analysis

First Author	BMI (Kg/m^2) cases	Criteria or characteristics of patients group	Control group criteria or features	NOS
Georges 2001	NA	The degree of CAD was assessed by the number of major arteries with greater than 50 % stenosis.	NA	7
Humphries 2001	26.9	NA	Free of a history of unstable angina, MI or evidence of a silent infarction, coronary surgery, aspirin or anticoagulant therapy, CVA, malignancy and others.	7
Jenny 2002	27	CVD was defined as confirmed angina, MI, stroke, or TIA	Composed of randomly selected original cohort members who had no prevalent or incident CVD and no MRI-detectable infarcts and who did not satisfy the criterion of being free of subclinical CVD.	7
Revilla 2002	NA	Lacunar stroke was diagnosed if the patient had pure motor hemiparesis, sensory-motor syndrome, ataxic hemiparesis, pure sensory syndrome or dysarthria-clumsy hand, respectively, and a brain CT scan (100 %) or MRI (90 %) disclosed acute normal findings or a deep focal infarction.	Without history or current symptoms of ischemic or hemorrhagic stroke was selected from the same geographic area random digit dialing.	7
Flex 2002	NA	Diagnosis of PAOD was performed in accordance with the criteria established by the Ad Hoc Committee on Reporting Standards of the Society for Vascular Surgery and the International Society for Cardiovascular Surgery.	Exclusion criteria from the study were tumors, chronic inflammatory diseases, and autoimmune diseases.	7
Nauck 2002	27.52	Inclusion criteria for were German ancestry, clinical stability except for acute coronary syndromes, and availability of a CA. Exclusion criteria were any acute illness other than acute coronary syndromes, any non-cardiac chronic disease, and a history of malignancy within the past 5 years.	NA	7
Basso 2002	26	Individuals who, during 4.8 years of follow-up, experienced a definite fatal or nonfatal MI or sudden coronary death or who required CABG or angioplasty were defined as cases.	NA	6
Bennet 2003	26	Subjects that survived 28 days after their first MI, and had no further events before blood sampling.	NA	8
Greisenegger 2003	NA	Patients underwent cranial CT or MRI, laboratory investigations for vascular risk factors, duplex sonography of the carotid and vertebral arteries, and a thorough cardiac investigation.	Controls were free of clinically manifest vascular disease and were matched individually to the patients for age.	8

First Author	BMI (Kg/m^2) cases	Criteria or characteristics of patients group	Control group criteria or features	NOS
Stephens 2004	29.08	CVD was recorded if a patient had one or more of CHD, PVD or CbVD. CHD was recorded if any patient had positive CA or angioplasty, coronary artery bypass, a positive cardiac thallium scan or exercise tolerance test, documented evidence of myocardial infarction or symptomatic/treated angina.	Subjects who were asymptomatic for CHD/ CbVD/PVD or had negative investigations were categorized as having no CVD.	7
Licastro 2004	NA	Patients had no history of neoplastic, autoimmune diseases, coagulation disorders or chronic renal failure.	Free of neoplastic, autoimmune inflammatory diseases and in apparent good health.	8
Flex 2004	NA	The cerebral ischemic event had been documented by CT scan or magnetic MRI of the brain.	Controls had no relationship with cases and no family history of stroke.	6
Balding 2004	NA	Stroke was defined as acute onset of neurological deficit lasting more than 24 hours or leading to death with no apparent cause other than cerebrovascular disease.	NA	7
George 2004	NA	NA	NA	6
Lieb 2004	27.8	The diagnosis of MI was established according to the MONICA diagnostic criteria.	NA	8
Kelberman 2004	27.1	MI excluding patients with familial hypercholesterolemia and type 1 diabetes mellitus.	NA	8
Rosner 2005	25.5	MI was confirmed if it met the WHO criteria, which include symptoms in the presence of either elevation of cardiac enzymes or diagnostic changes on the electrocardiogram. For fatal events, the diagnosis of MI was also accepted based on autopsy findings.	Consisted of those who provided a blood sample at baseline and remained free of CVD at the time the index event occurred in the case subject.	7
Karahan 2005	NA	Stroke was made clinically and the infarction was verified by MRI of the brain.	Unrelated adults living in the same geographic areas in Turkey without a personal or family history of stroke.	8
Chiapelli 2005	5.5	Patients had no history of neoplastic, autoimmune diseases, coagulation disorders or chronic renal failure	Free of neoplastic, autoimmune and inflammatory diseases and in apparent good health.	8
Danielsson 2005	NA	Recruitment was from patients referred to the Department of Vascular Diseases because of vascular symptoms.	Healthy blood donors.	7
Weger 2005	NA	Exclusion criteria for patients with RAO comprised giant cell arteritis and other types of vasculitis.	Subjects with any ophthalmological evidence or history of retinal vascular occlusion, anterior ischemic optic neuropathy, or vasculitis were not eligible as controls.	7
Densem 2005	NA	CVD was defined as a stenosis of greater than 50 % in one or more major epicardial coronary arteries. Two angiographers, blinded to clinical and genetic information, independently reviewed all studies.	NA	6

EXCLI Journal 2019;18:331-355 – ISSN 1611-2156
Received: March 06, 2019, accepted: April 09, 2019, published: June 07, 2019

First Author	BMI (Kg/m^2) cases	Criteria or characteristics of patients group	Control group criteria or features	NOS
Chamorro 2005	NA	Patients had a brain CT scan or a brain MRI to differentiate between ischemic and hemorrhagic stroke. Patients also had the necessary diagnostic workup aimed at establishing the stroke subtype, including extracranial and transcranial Doppler sonography, ECG, MRI, examination of prothrombotic disorders and cerebral angiography.	Subjects without a history of cerebrovascular symptoms were selected by random-digit dialing of the same geographic area of residence.	6
Sie 2006	26	CHD was defined as the occurrence of an MI, revascularization procedure (percutaneous transluminal coronary angioplasty or coronary artery bypass graft), IHD, sudden cardiac death, ventricular fibrillation or tachycardia, congestive heart failure, or sudden death undefined during follow-up.	NA	7
Mysliwska 2006	28	Qualification for surgery required that at least one of the major coronary vessels was characterized with ≥ 75 % stenosis. The following coronary arteries were affected: LAD or/and RCA or/and CX.	Never presented clinical symptoms of CHD and had a normal resting and exercise-related electrocardiogram. They did not have any history of arterial hypertension or diabetes mellitus.	7
Lalouschek 2006	NA	The diagnosis was established clinically and all patients underwent CT or MRI.	All were free of clinically manifest arterial vascular disease and reported no vascular diseases in first-degree relatives. Medical history, including a history of febrile conditions within the last 4 weeks, vascular risk factors and results of laboratory investigation.	8
Tütün 2006	--	Patients below 35 years of age who underwent CABG between September 2002 and September 2004 constituted the study group.	Age and sex-matched healthy controls.	6
Sekuri 2007	26.5	Stenosis of at least 50 % in a major coronary artery, or one of their branches, as determined by CA. The extension of disease was defined as the number of arteries with stenosis at least 50 % as single or multiple vessels. The CA was performed by Judkin’s method at the catheterization laboratories.	NA	9
Potacsek 2007	--	--	--	8
Flex 2007	NA	Presence of PAOD at Fontaine’s stage II, III, or IV. Diagnosis of PAOD was performed in accordance with the criteria established by the Ad Hoc Committee on Reporting Standards of the Society for Vascular Surgery and the International Society for Cardiovascular Surgery.	Subjects presented an ankle/arm pressure index ‡1 and normal findings at bilateral high-resolution B-mode ultrasonography evaluation. Exclusion criteria from the study were cancer, chronic inflammatory and infectious diseases, and autoimmune diseases.	7

EXCLI Journal 2019;18:331-355 – ISSN 1611-2156
Received: March 06, 2019, accepted: April 09, 2019, published: June 07, 2019

First Author	BMI (Kg/m^2) cases	Criteria or characteristics of patients group	Control group criteria or features	NOS
Tretjakovs 2007	32.9	Coronary artery stenosis >50 %.	Healthy subjects	6
Humphries 2007	NA	CHD endpoints were acute CHD events, sudden coronary death, fatal acute myocardial infarction and non-fatal acute myocardial infarction.	Exclude any CVD.	7
Smallwood 2008	27.2	Men with small AAAs (aortic diameter 30-50 mm) were invited to attend a follow-up study (1997-2004) involving repeat ultrasound scans at intervals of 6-12 months. At the first follow-up visit, a sample of venous blood was obtained from all consenting men. Men without AAAs were also reviewed as part of a separate study in 2001-4 which also included venous blood sampling.	Consisted of randomly selected men with aortic diameters in a previously defined reference range of 19-22 mm.	7
Banerjee 2008	NA	Subjects were diagnosed at the onset using either CT or MRI.	Subjects had vascular risk factors such as hypertension, diabetes mellitus, smoking habit, and hypercholesterolemia but without any history or occurrence of cerebrovascular or CVD.	7
Maitra 2008	25.76	The families were recruited through the probands who had a history of premature CAD, which included stable and unstable angina and MI diagnosed by ECG and treated based on the catheter lab availability with standard medication or CA followed by PTCA or CABG.	Individuals who did not have any personal or family history of CAD, diabetes mellitus or hypertension.	8
Smith 2008	28.2	NA	NA	7
Sarecka 2008	26.9	CAD with more than 50 % diameter stenosis of at least one of the major coronary vessels.	The exclusion criterion was CAD or stroke revealed in the course of family history. CAD in this case was defined by its occurrence in at least one of the parents.	8
Silander 2008	29*	Baseline information on all randomly sampled individuals includes anthropometric measurements, serum lipids, blood pressure and questionnaire data on CVD risk factors.	Sub-cohort was sex- and geographic-region stratified random sample, drawn from each of the original cohorts with unequal sampling probabilities so that the age distribution was similar to the cases. The selection procedure for the cases and the sub-cohort, and the exact diagnostic criteria used for CHD and ischemic stroke have been described in detail previously.	7
Banerjee 2009	NA	CA showing 50 % stenosis of at least one segment of a major coronary artery was defined as CAD.	Subjects had vascular risk factors such as hypertension, diabetes mellitus, and smoking habit but had no history of cardiac diseases, no symptoms of other atherosclerotic vascular diseases and had normal ECG.	8

EXCLI Journal 2019;18:331-355 – ISSN 1611-2156
Received: March 06, 2019, accepted: April 09, 2019, published: June 07, 2019

First Author	BMI (Kg/m^2) cases	Criteria or characteristics of patients group	Control group criteria or features	NOS
Panoulas 2009	27	CVD was defined as the presence of cardiovascular death, CHD, CVA or PVD. CHD was defined as having any of the following: MI, angioplasty, CABG or angina diagnosed by a physician or elicited by the use of the Rose questionnaire.	NA	7
Berg 2009	NA	Significant (>50 %) coronary artery stenosis in at least one main coronary artery branch.	The group had no significant coronary artery stenosis.	7
Ghazouani 2010	27	All patients included had evidence of CAD documented by prior cardiac bypass surgery, or documented ACS. CAD was defined as 50 % stenosis in the left main coronary artery, or multiple significant (≥70 % stenosis) in more than one coronary artery.	They were undergoing a routine check-up as part of pre-employment requirements, which included an ECG, chest X-ray, and serum analysis. They were classified as healthy as their physical examination was unremarkable, coupled with the absence of personal or family history and reasons to suspect CAD.	8
Tong 2010	NA	IS was established using the WHO, International Classification and stroke subtypes were defined using the Oxford shire classification.	Subjects with a history of stroke, Alzheimer's disease, brain aneurysm, dementia, dystonia, Parkinson's disease or inflammatory disorders were excluded.	8
Rios 2010	NA	These individuals were classified as CAD if they presented at least one obstructive lesion P50 %.	They were used as controls if they did not show any obstructive lesion in the angiography. Moreover, none presented MI, cerebral vascular infarction or TIA.	7
Fan 2011	NA	CHD patients had a medical examination. In general, patients were required to have coronary catheterization demonstrating significant CHD (at least a 50 % stenosis in one major epicardial coronary vessel).	Subjects without CHD and CP were recruited from a community health checkup to match the age and gender distribution of CHD and CP patients as much as possible.	8
Ghazouani 2011	27	Diagnosis of CAD was based on CA (obstructive coronary lesions with >50 % narrowing of any subepicardial coronary artery). Determination of CAD severity was based on the number of major epicardial coronary arteries affected.	They were required to have a normal physical examination, a normal resting ECG and no personal or family history of CAD. All participants were asked to fill a standard questionnaire detailing demographic details, together with data on traditional CAD risk factors.	8
Coker 2011	28.4	MI was defined using the standard Cardiovascular Health Study criteria: history of chest pain, cardiac enzyme levels and characteristic changes on serial electrocardiograms.	Patients who came to the hospital due to the chest pain and general health ensure checkup. The exclusion criteria were pregnancy, vascular heart disease, atrial fibration, acute or chronic infectious, immunological conditions, history of malignancies, neoplastic, coagulation disorders or chronic renal failure.	9

EXCLI Journal 2019;18:331-355 – ISSN 1611-2156
Received: March 06, 2019, accepted: April 09, 2019, published: June 07, 2019

First Author	BMI (Kg/m^2) cases	Criteria or characteristics of patients group	Control group criteria or features	NOS
Bennermo 2011	26.8	They were found to have their first MI were enrolled in a clinical research program targeting mechanisms underlying premature CHD.	NA	8
Vakili 2011	NA	MI was established by WHO criteria and was confirmed by CA and left ventriculography. However, previous MI was verified by an episode of persistent ST elevation or depression in ECG and raised serum cardiac markers.	Without evidence of MI, hypertension, diabetes, or cardiovascular diseases.	8
Tuttolomondo 2012	NA	Stroke was defined by focal neurological signs or symptoms thought to be of vascular origin that persisted for >24 h, confirmed by brain CT and/or MRI in baseline conditions and brain CT with contrast medium after 48–72 h.	Patients admitted for any cause other than acute cardiovascular and CVA and for routine checkup examinations.	7
Tong 2013	23.2	CA evidence of 70 % stenosis of one major coronary artery, or 50 % stenosis of the left main coronary artery. Exclusion criteria were: current heparin treatment; autoimmune disease; congenital heart disease; severe kidney or liver disease; malignancy.	Subjects with known CAD or any other heart disease were excluded from the stud.	8
Chakraborty 2013	NA	Patients with a history of TIA, fever, rheumatologic disease, autoimmune disease, any acute or chronic infection, CT or MRI proved hemorrhagic stroke, and a history of regular immunosuppressive or analgesic therapies were excluded.	Routine laboratory investigations were normal and no controls had a history of stroke. The control group was expected, a history of hypertension, diabetes and dyslipidemia.	8
Satti 2013	25.9	CAD in at least two successive generations. The patients were confirmed on the basis of CA criteria established by Francois and electrocardiographic features.	Healthy controls representing the same geographical location were included on the basis of normal ECG, normal CA, and no history and symptoms of CVD.	7
Mishra 2013	24.39	CA identified stenosis >70 % in the major coronary vessels at the time of the study were used to classify patients as having single-vessel, double-vessel, or triple-vessel disease. CAD patients having any other cardiac disorder were excluded from the study.	No clinical evidence of CAD or LV dysfunction and also without a positive family history of CAD or MI.	8
Bhanushali 2013	NA	CAD confirmed by CA: >50 % stenosis in one or more arteries and stable or unstable angina.	Examined clinically and investigated by electrocardiography to exclude CAD.	8
Phulukdaree 2013	NA	CAD patients were Indian ancestry and unrelated, adults below the age of 45 years, and stable CAD confirmed at CA.	Exclusion criteria for controls were an ACS/revascularization procedure in the preceding 3 months, chronic renal or liver disease, malignancy, and known active inflammatory or infectious disease.	7

EXCLI Journal 2019;18:331-355 – ISSN 1611-2156
Received: March 06, 2019, accepted: April 09, 2019, published: June 07, 2019

First Author	BMI (Kg/m^2) cases	Criteria or characteristics of patients group	Control group criteria or features	NOS
Liaquat 2014	22.7	IDC diagnosed on echocardiographic evidence of LV enlargement, LV systolic dysfunction (ejection fraction ≤ 40 %), and end-diastolic diameter > 34 mm/m^2.	Normal electrocardiograms and ECG with no symptoms of any concomitant disease, and had no personal or familial history of cardiomyopathy or other heart-related diseases.	8
Galimudi 2014	NA	Excluding any known cause of myocardial disease.	No known history of any disease.	7
Sun 2014	NA	Angiographically documented CAD.	CAD or any other heart disease were excluded from participation.	8
Elsaid 2014	no available NA e	CA evidence of ≥ 70 % stenosis of one major coronary artery or ≥ 50 % stenosis of the left main coronary artery. Exclusion criteria included current heparin treatment, autoimmune disease, congenital heart disease, severe kidney or liver disease, or malignancy.	Subjects had any signs or symptoms suggesting CAD.	7
Li 2015	NA	Obese, subjects having virus C or B were excluded.	Subjects who had a history of CAD or cardiovascular disease, autoimmune disease, congenital heart disease, severe kidney or liver disease, or tumors were excluded from the study.	8
Spoto 2015	28.2	Using the results of electrocardiography, those who had obstructive lesions of more than 50 % in one or more coronary arteries were considered to have CAD.	NA	7
Yang 2015	NA	CKD and history of CVD. MI documented by electrocardiography and biomarkers of myocardial injury; heart failure, defined as dyspnea in addition to two of the following conditions: raised jugular pressure, bibasilar crackles, pulmonary venous hypertension, or interstitial edema on chest radiography requiring hospitalization; electrocardiography-documented arrhythmia; stroke; PVD; and major arterial or venous thrombotic episodes.	The control subjects were diagnosed as having no history of atherosclerotic lesions or CAD.	8
Wang 2015	22.8	CAD was diagnosed by CA, and CAD was defined as the presence of at least one significant coronary artery stenosis of ≥ 50 % luminal diameter, as identified by CA. The patient exclusion criteria were myocardial spasms or a myocardial bridge, congenital heart disease, childhood hypertension, severe kidney or liver disease, or malignant tumors.	Collected from subjects who had taken a health examination as part of our study. Control subjects who suffered from CAD or any other heart disease were excluded from the study.	8
Salama 2015	NA	Diagnosed by CA evidence of ≥ 70 % stenosis of one major coronary artery, or ≥ 50 % stenosis of the left main coronary artery.	Third control group includes 34 volunteers with no previous history of TIA.	7

EXCLI Journal 2019;18:331-355 – ISSN 1611-2156
Received: March 06, 2019, accepted: April 09, 2019, published: June 07, 2019

First Author	BMI (Kg/m^2) cases	Criteria or characteristics of patients group	Control group criteria or features	NOS
		Patients who had an autoimmune disease, congenital heart disease, severe kidney or liver disease, or malignancy were excluded from our study.		
Buraczynska 2016	28.1	Multiple TIAs history with at least one recent attack (high-risk TIA) and with one TIA over the last year (low-risk TIA).	Without a history of diabetes or cardiovascular events and no signs of dysfunction of the cardiovascular system upon examination.	8
Hongmei 2016	26.41	T2DM with CVD. CVD was defined as congestive heart failure, ischemic cerebral stroke, or peripheral arteriopathy.	Confirmed to be free of coronary artery diseases and other CVD, end-stage renal and liver disease, serious infections, malignant tumor, thyroid disease and immune system diseases.	9
Mao 2016	24.61	CAD was defined as follows: luminal stenosis above 50 % in one of the main coronary arteries or their branch retinal arteries, and exhibiting stable angina, unstable angina pectoris and MI.	Subjects were randomly selected from the physical examination center or the outpatient clinics at the Henan Provincial People's Hospital. These subjects were confirmed to have no history of arteriosclerotic lesions or CVD.	8
Kou 2017	25.8	CAD was defined as a diameter stenosis of above 70 % in any main coronary arteries. Patients who had malignancies, myocardial spasms, myocardial bridges, as well as those suffering from autoimmune diseases, congenital heart diseases, or end-stage kidney or liver diseases were excluded from the study.	Diabetes, hypertension, hyperlipidemia, and missing data and those with BMI < 18.5 kg/m^2.	8
Jun 2017	24.7	ITC (cardiac catheterization, percutaneous coronary intervention, or coronary artery bypass grafting), stable angina pectoris, unstable angina pectoris, the first occurrence of acute MI, congestive heart failure caused by myocardial ischemia after baseline investigation, ischemic stroke, hemorrhagic stroke, and peripheral vascular disease, or cardiovascular death.	The controls were randomly selected from those, who received a physical examination in our hospital and 1:1 matched to cases on the basis of age and sex, all selected controls received electrocardiographic testing and CA, and those with abnormal electrocardiographic and CA profile were excluded.	8
Mitrokhin 2017	30.71	CHD patients were diagnosed by at least two experienced cardiologists and were confirmed by CA (> 50 % diameter stenosis in at least one of the major coronary arteries) according to the WHO criteria for the CHD confirmation.	The control subjects were confirmed to be free of CAD and other CVD, end-stage renal and liver diseases, thyroid disease, malignant tumor, immune system diseases, and serious infections.	8
Mastana 2017	NA	The basic criteria for inclusion in the group with CAD was ≥ 50 % stenosis in at least one coronary artery (left main coronary stenosis, right coronary artery, anterior descending branch, and CX) and exhibiting stable or unstable angina pectoris.	Controls had no known history of IHD, hypertension, diabetes, endocrine or metabolic disorders and were selected after administration of a treadmill exercise test	9

EXCLI Journal 2019;18:331-355 – ISSN 1611-2156
Received: March 06, 2019, accepted: April 09, 2019, published: June 07, 2019

First Author	BMI (Kg/m^2) cases	Criteria or characteristics of patients group	Control group criteria or features	NOS
			to exclude the possibility of the patients having an underlying CAD.	
Jabir 2017	28.69	Patients were classified on the basis of at least 50 % or more stenosis in one or more coronary arteries verified through CA.	Healthy subject	7
Akinyemi 2017	NA	CAD was diagnosed by CA and other routinely used biological parameters.	NA	7

Peripheral vascular disease (PVD); cerebrovascular disease (CbVD); magnetic resonance imaging (MRI); left anterior descending (LAD); right coronary artery (RCA), circumflex artery (CX); computed tomography (CT); coronary angiography (CA); echocardiogram (ECG); transluminal coronary angioplasty (PTCA); coronary artery bypass graft (CABG); cerebrovascular accident (CVA), peripheral vascular disease (PVD); acute coronary syndrome (ACS); idiopathic dilated cardiomyopathy (IDCM); left ventricular (LV); interventional therapy of coronary (ITC); ischemic heart disease (IHD); transient ischemic attack (TIA); Abdominal aortic aneurysm (AAA); not available (NA).

EXCLI Journal 2019;18:331-355 – ISSN 1611-2156
Received: March 06, 2019, accepted: April 09, 2019, published: June 07, 2019

Supplementary Table 2: Meta-analysis of the association between rs1800795 polymorphism and cardiovascular disease risk in accordance with the geographical location in which the studies were performed.

Study groups	Allele						Homozygote						Heterozygote					
		OR (95% CI)	P	I^2	Q	Egger		OR (95% CI)	P	I^2	Q	Egger		OR (95% CI)	P	I^2	Q	Egger
Overall	74	1.43(1.06-1.22)	**<0.001**	82.32	<0.00	0.398	70	1.24(1.08-1.42)	**0.002**	75.40	<0.00	0.360	71	1.14(1.04-1.24)	**0.003**	72.20	<0.00	0.399
	52	1.06(1.02-1.10)	**0.001**	15.56	0.172	0.943	49	1.11(1.03-1.19)	**0.006**	11.11	0.256	0.184	62	1.08(1.03-1.12)	**<0.00**	0.000	0.608	0.675
China	11	1.36(1.26-1.48)	**<0.001**	5.466	0.391	0.663	8	1.91(1.61-2.27)	**<0.001**	0.000	0.734	0.184	11	1.21(1.09-1.34)	**<0.00**	0.000	0.843	0.179
United Kingdom	9	1.19(0.95-1.49)	0.111	88.28	<0.00	0.128	9	1.26(0.84-1.89)	0.261	84.65	<0.00	0.442	9	1.16(0.96-1.39)	0.114	52.95	0.030	0.292
	7	0.99(0.92-1.08)	0.987	0.000	0.815	0.591	7	0.94(0.79-1.11)	0.488	0.000	0.862	0.469	8	1.16(1.02-1.31)	**0.018**	10.38	0.350	0.358
Turkey	4	1.07(0.75-1.53)	0.676	52.02	0.100	0.226	4	1.02(0.43-2.41)	0.957	44.43	0.145	0.157	4	1.00(0.75-1.34)	0.967	0.000	0.929	0.499
	3	0.94(0.74-1.19)	0.621	0.000	0.908	0.408	3	0.81(0.44-1.48)	0.495	0.000	0.877	0.117						
India	8	0.93(0.73-1.17)	0.550	58.26	0.019	0.737	8	0.89(0.47-1.67)	0.730	37.42	0.131	0.993	8	0.91(0.67-1.24)	0.584	62.78	0.009	0.640
	7	1.05(0.89-1.24)	0.536	0.000	0.626	0.106	7	1.26(0.71-2.23)	0.415	0.000	0.464	0.200	7	1.05(0.86-1.28)	0.610	0.000	0.563	0.968
Europeans	37	1.10(0.99-1.21)	0.059	86.63	<0.00	0.581	36	1.08(0.91-1.28)	0.349	79.72	<0.00	0.911	35	1.18(1.02-1.36)	**0.022**	83.47	<0.00	0.536
	27	1.03(0.99-1.07)	0.130	12.04	0.286	0.900	27	1.04(0.96-1.13)	0.305	4.894	0.392	0.084	26	1.05(0.98-1.12)	0.123	11.36	0.298	0.174
Africans	3	0.91(0.64-1.30)	0.637	59.15	0.086	0.296	3	0.94(0.28-2.86)	0.864	47.92	0.142	0.296	3	1.03(0.77-1.38)	0.801	0.000	0.553	0.877

Study groups	Dominant						Recessive					
		OR (95% CI)	P	I^2	Q	Egger		OR (95% CI)	P	I^2	Q	Egger
Overall	74	1.16(1.06-1.27)	**0.001**	76.18	<0.00	0.404	71	1.18(1.04-1.34)	**0.008**	77.66	<0.00	0.450
	60	1.12(1.07-1.18)	**<0.001**	13.26	0.197	0.504	53	1.05(0.98-1.12)	0.124	10.41	0.262	0.634
China	10	1.16(1.05-1.27)	**0.002**	0.000	0.998	0.596	8	1.78(1.51-2.10)	**<0.001**	0.000	0.717	0.720
United Kingdom	9	1.15(1.00-1.31)	**0.039**	29.28	0.185	0.166	9	1.25(0.73-2.13)	0.399	92.70	<0.00	0.387
	8	1.10(0.98-1.23)	0.095	6.137	0.383	0.350	7	0.86(0.74-1.00)	0.055	0.000	0.921	0.235
Turkey	4	1.01(0.77-1.34)	0.911	0.000	0.519	0.243	4	1.01(0.43-2.35)	0.971	44.01	0.147	0.170
							3	0.81(0.45-1.47)	0.496	0.000	0.843	0.108
India	8	0.91(0.67-1.22)	0.540	64.32	0.006	0.811	8	0.97(0.59-1.58)	0.912	12.77	0.330	0.675
	7	1.05(0.87-1.27)	0.564	0.000	0.660	0.458						
Europeans	37	1.17(1.02-1.35)	**0.018**	83.97	<0.00	0.337	37	1.02(0.87-1.20)	0.764	82.57	<0.00	0.689
	29	1.07(1.00-1.14)	**0.026**	19.70	0.174	0.191	28	1.01(0.95-1.09)	0.592	0.000	0.515	0.599
Africans	3	0.94(0.57-1.54)	0.814	31.68	0.231	0.758	3	0.93(0.49-1.79)	0.843	37.73	0.201	0.654

EXCLI Journal 2019;18:331-355 – ISSN 1611-2156
Received: March 06, 2019, accepted: April 09, 2019, published: June 07, 2019

Supplementary Table 3: Meta-analysis of the association between rs1800795 polymorphism and clinical diagnosis

Pathologies	Allele					Homozygote				
	**OR (95% CI)**	**P**	**I^2**	**Q**	**Egger**	**OR (95% CI)**	**P**	**I^2**	**Q**	**Egger**
CAD	27 1.16(1.03-1.30)	**0.011**	73.17	<0.00	0.386	26 1.39(1.11-1.74)	**0.004**	0.58	<0.00	0.514
	19 1.14(1.04-1.23)	**0.002**	21.14	0.197	0.692	18 1.50(1.28-1.76)	**<0.001**	0.000	0.522	0.148
PAOD	4 0.72(0.46-1.11)	0.142	79.38	0.002	0.618	4 0.52(0.24-1.11)	0.094	73.21	0.011	0.588
MI	13 1.08(0.99-1.18)	0.083	69.06	<0.00	0.115	13 1.16(0.96-1.40)	0.112	68.21	<0.00	0.093
	10 1.00(0.92-1.05)	0.832	0.000	0.561	0.887	12 1.03(0.93-1.15)	0.498	12.02	0.327	0.164
IS	10 0.92(0.73-1.16)	0.498	76.21	<0.00	0.991	9 0.86(0.47-1.59)	0.649	78.37	<0.00	0.645
	9 1.02(0.91-1.14)	0.671	0.000	0.497	0.392	6 1.07(0.73-1.58)	0.703	3.559	0.394	0.531
HC	53 1.23(1.12-1.34)	**<0.001**	81.19	<0.00	0.450	50 1.42(1.20-1.67)	**<0.001**	69.96	<0.00	0.300
	33 1.12(1.07-1.18)	**<0.001**	0.000	0.642	0.100	35 1.23(1.11-1.37)	**<0.001**	5.150	0.382	0.879
	**Dominant**					**Recessive**				
	**OR (95% CI)**	**P**	**I^2**	**Q**	**Egger**	**OR (95% CI)**	**P**	**I^2**	**Q**	**Egger**
CAD	27 1.15(1.02-1.30)	**0.019**	56.72	<0.00	0.646	26 1.32(1.08-1.61)	**0.005**	57.39	<0.00	0.912
	25 1.23(1.11-1.35)	**<0.001**	20.82	0.175	0.836	18 1.31(1.10-1.56)	**0.002**	15.54	0.268	0.232
PAOD	4 0.80(0.42-1.50)	0.487	78.20	0.003	0.347	4 0.52(0.31-0.86)	**0.012**	52.95	0.095	0.894
						3 0.39(0.26-0.59)	**<0.001**	0.000	0.543	0.305
MI	13 1.11(0.97-1.27)	**0.099**	69.58	<0.00	0.084	13 1.09(0.95-1.25)	0.202	54.87	0.009	0.184
	9 0.96(0.89-1.04)	0.394	0.000	0.960	0.742	12 1.02(0.94-1.11)	0.594	0.000	0.944	0.476
IS	10 0.93(0.70-1.23)	0.614	60.31	0.007	0.765	9 0.84(0.54-1.30)	0.442	76.71	<0.00	0.930
	9 1.07(0.89-1.29)	0.427	0.856	0.427	0.180	8 0.99(0.79-1.25)	0.977	18.68	0.282	0.641
HC	53 1.28(1.14-1.44)	**<0.001**	77.17	<0.00	0.451	50 1.31(1.11-1.54)	**<0.001**	76.56	<0.00	0.309
	43 1.24(1.16-1.31)	**<0.001**	4.082	0.392	0.216	37 1.02(0.95-1.10)	0.519	0.000	0.544	0.671

Supplementary Table 3 (cont.): Meta-analysis of the association between rs1800795 polymorphism and clinical diagnosis

Pathologies	Heterozygote					
		OR (95% CI)	P	I^2	Q	Egger
CAD	26	1.07(0.97-1.19)	0.152	30.93	0.069	0.759
	25	1.10(1.02-1.19)	**0.013**	0.000	0.804	0.439
PAOD	4	0.97(0.52-1.79)	0.928	72.94	0.011	0.362
MI	13	1.09(0.96-1.24)	0.155	63.45	<0.00	0.086
	10	0.98(0.91-1.06)	0.741	0.000	0.595	0.868
IS	10	0.98(0.79-1.23)	0.918	33.57	0.139	0.431
	9	1.07(0.88-1.32)	0.469	10.01	0.352	0.164
HC	52	1.23(1.09-1.40)	**<0.001**	76.61	<0.00	0.754
	42	1.17(1.10-1.24)	**<0.001**	0.000	0.901	0.366

Supplementary Table 4: Meta-analysis of the association between rs1800795 polymorphism and smoking habits

Study groups	Smokers vs non-smokers					
	(n)	OR (95% CI)	P	I^2	Q	Egger
Allelic	6	1.13(0.97-1.31)	0.116	62.54	0.020	0.707
	3	1.34(0.94-1.92)	0.103	29.03	0.244	0.296
Homozygous	6	1.10(0.83-1.46)	0.499	55.23	0.048	0.132
	5	1.01(0.88-1.16)	0.853	10.80	0.344	0.807
Heterozygous	6	1.18(0.54-2.54)	0.672	97.17	0.000	0.170
	3	1.08(0.70-1.67)	0.724	0.000	0.824	0.624
Dominant	6	1.18(0.74-1.87)	0.472	92.72	0.000	0.169
	3	1.28(0.85-1.91)	0.226	0.000	0.534	0.632
Recessive	6	1.11(0.67-1.83)	0.685	89.64	0.000	0.130
	3	1.18(0.73-1.91)	0.480	0.000	0.420	0.792

(n)= studies included

EXCLI Journal 2019;18:331-355 – ISSN 1611-2156
Received: March 06, 2019, accepted: April 09, 2019, published: June 07, 2019

Supplementary Figures

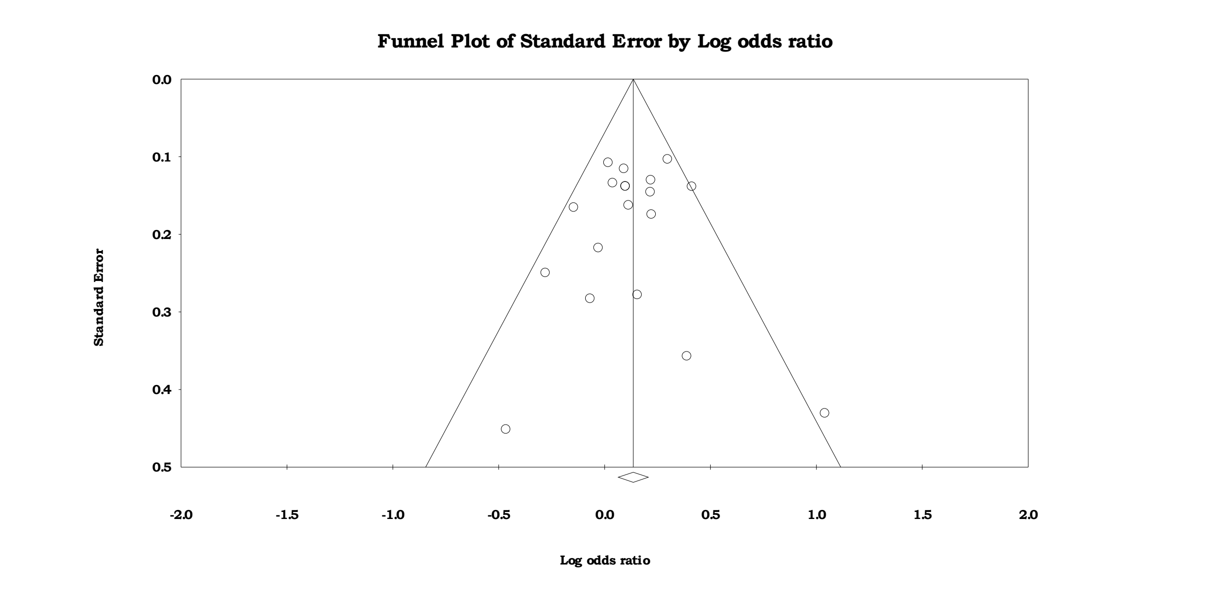

Funnel plot of the allelic model in the CAD population group in absence of heterogeneity

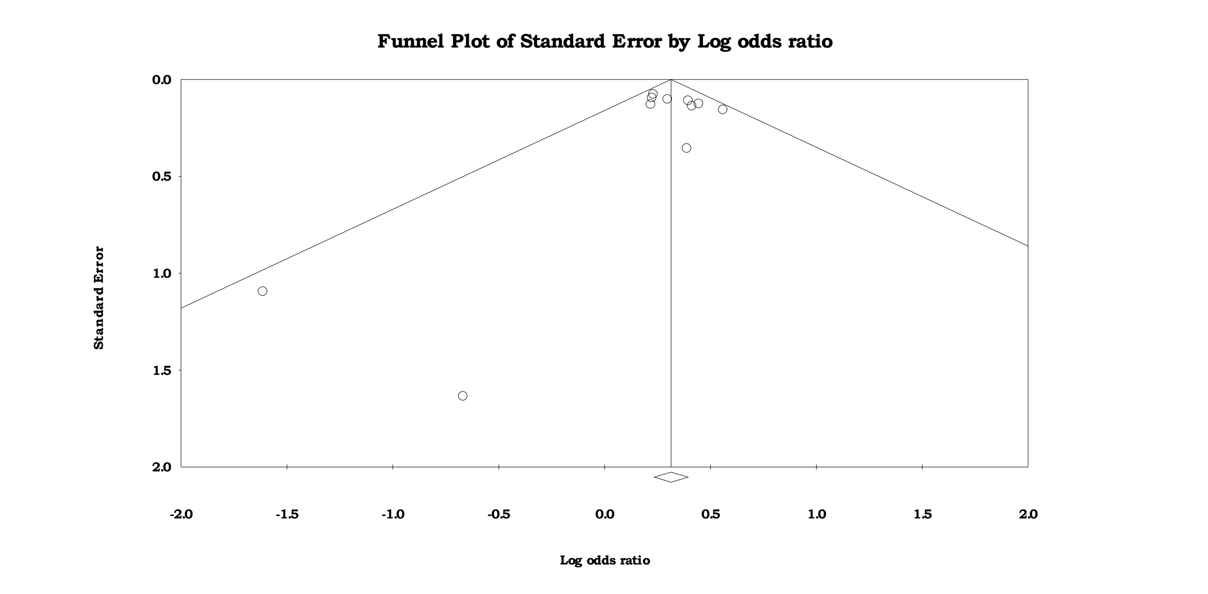

Funnel plot of the allelic model in the China population group in absence of heterogeneity

EXCLI Journal 2019;18:331-355 – ISSN 1611-2156
Received: March 06, 2019, accepted: April 09, 2019, published: June 07, 2019

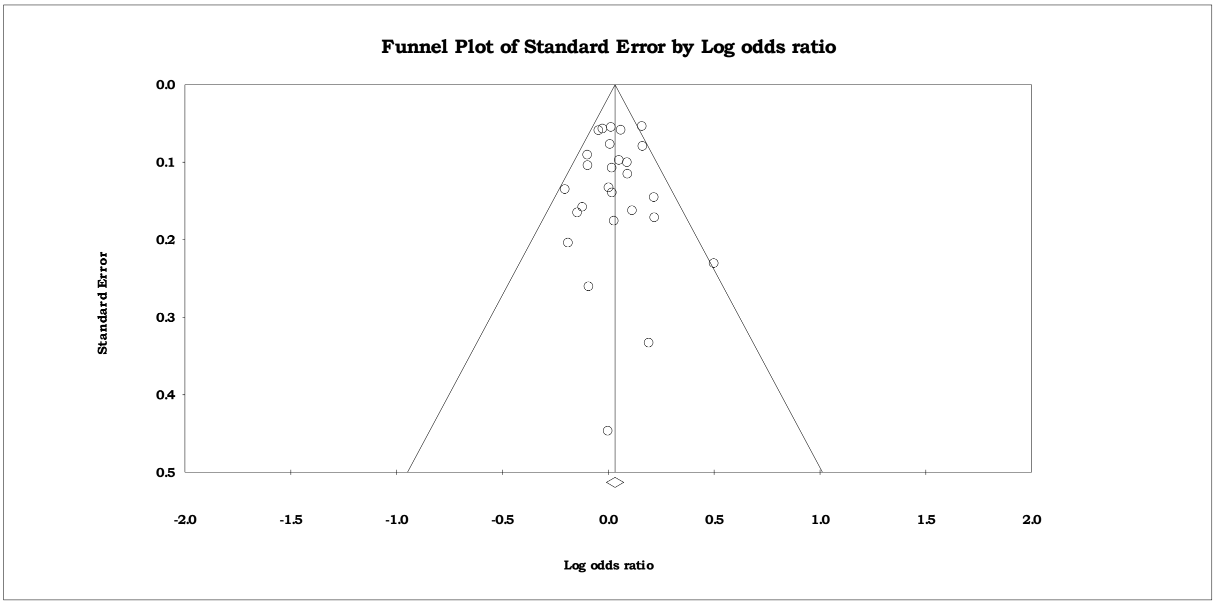

Funnel plot of the allelic model in the European population group in absence of heterogeneity

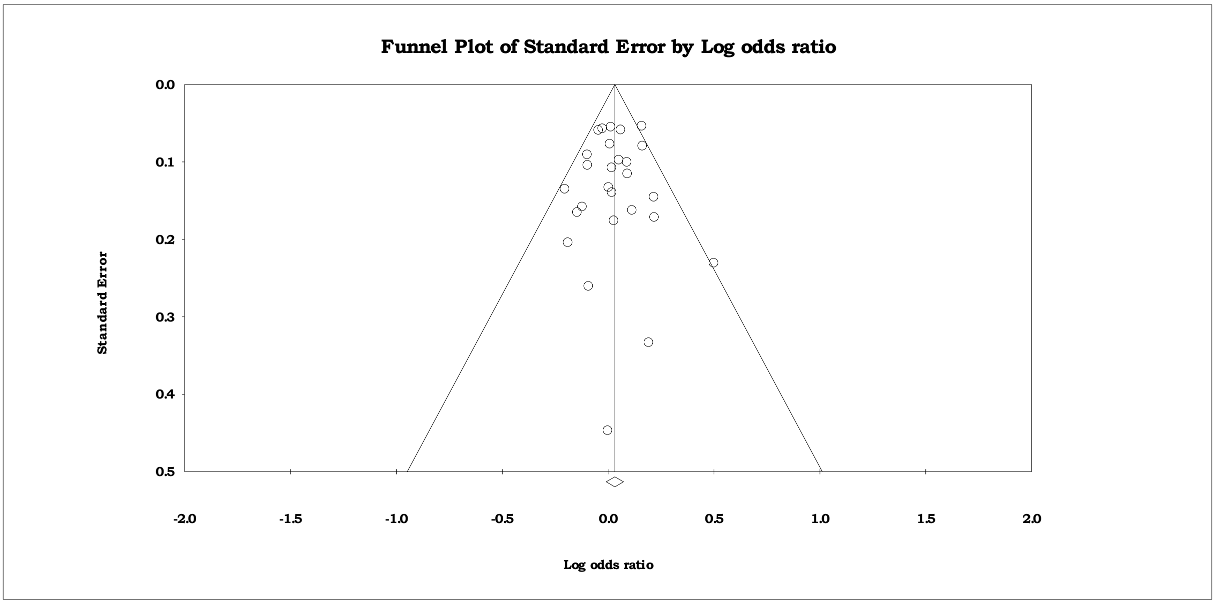

Funnel plot of the allelic model in the European population group in absence of heterogeneity

EXCLI Journal 2019;18:331-355 – ISSN 1611-2156
Received: March 06, 2019, accepted: April 09, 2019, published: June 07, 2019

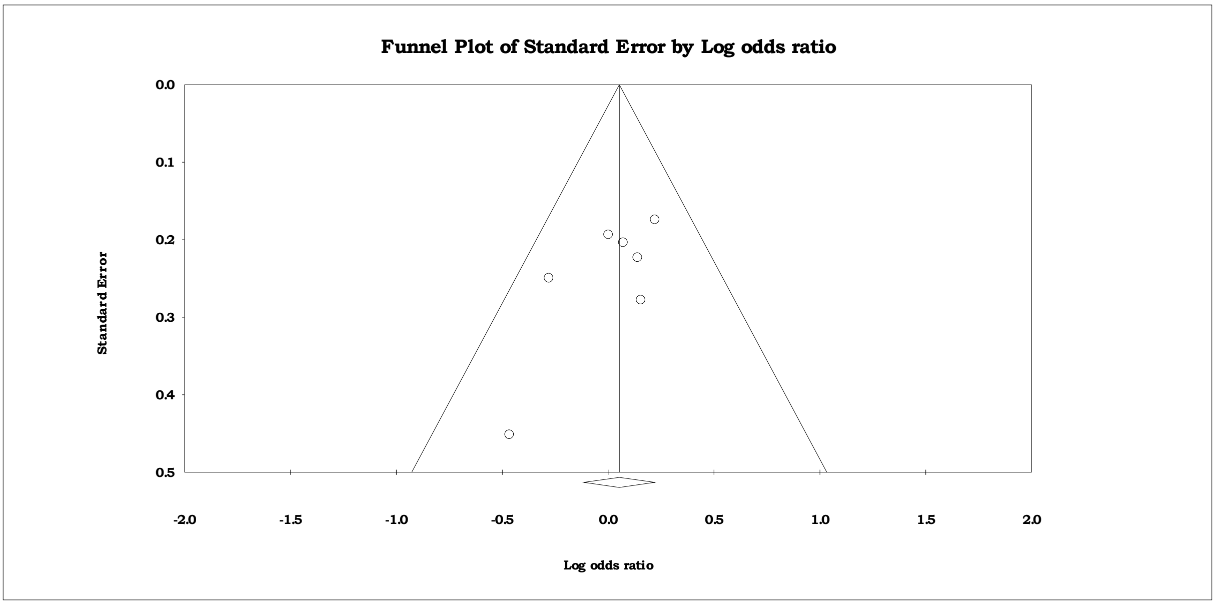

Funnel plot of the allelic model in the Indian population group in absence of heterogeneity

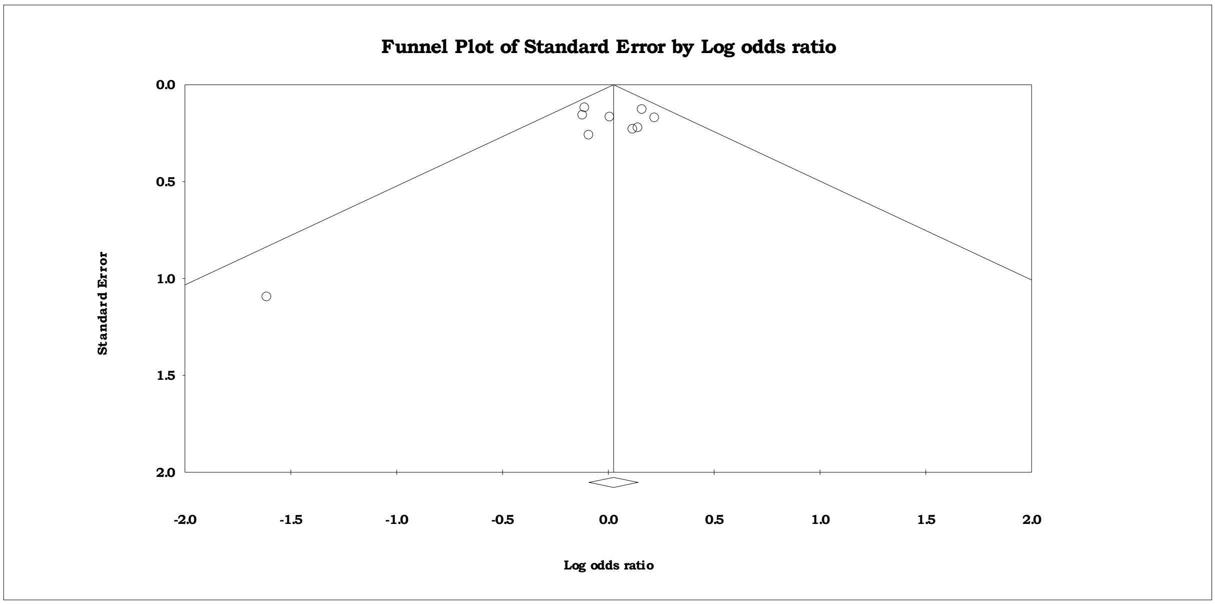

Funnel plot of the allelic model in the IS population group in absence of heterogeneity

EXCLI Journal 2019;18:331-355 – ISSN 1611-2156
Received: March 06, 2019, accepted: April 09, 2019, published: June 07, 2019

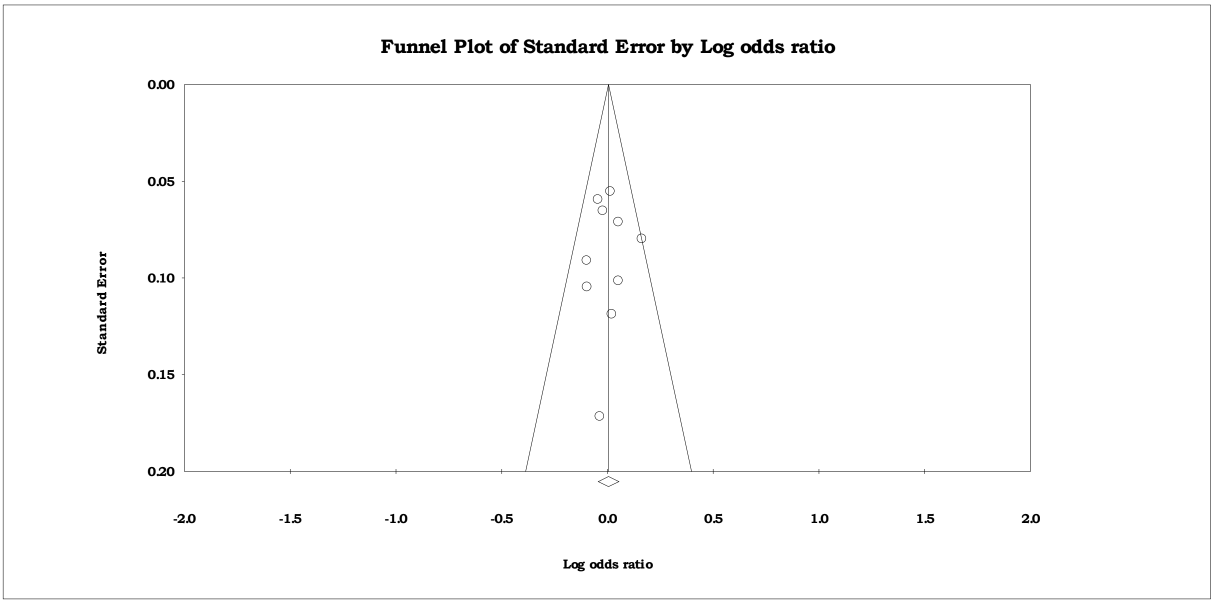

Funnel plot of the allelic model in the MI population group in absence of heterogeneity

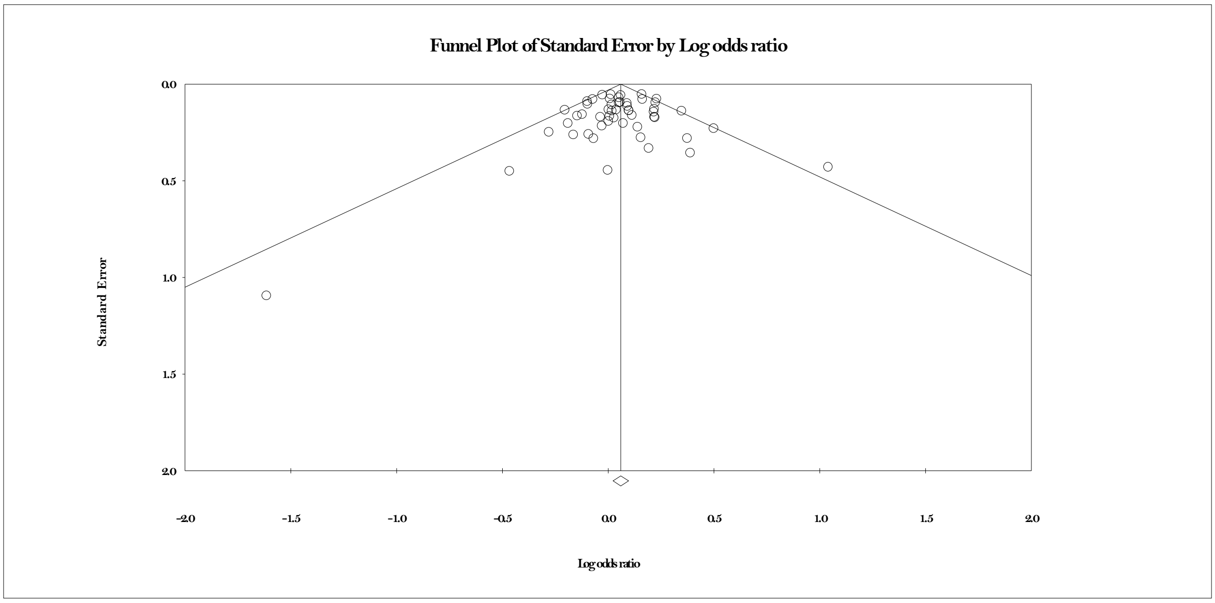

Funnel plot of the allelic model in the Overall population group in absence of heterogeneity

EXCLI Journal 2019;18:331-355 – ISSN 1611-2156
Received: March 06, 2019, accepted: April 09, 2019, published: June 07, 2019

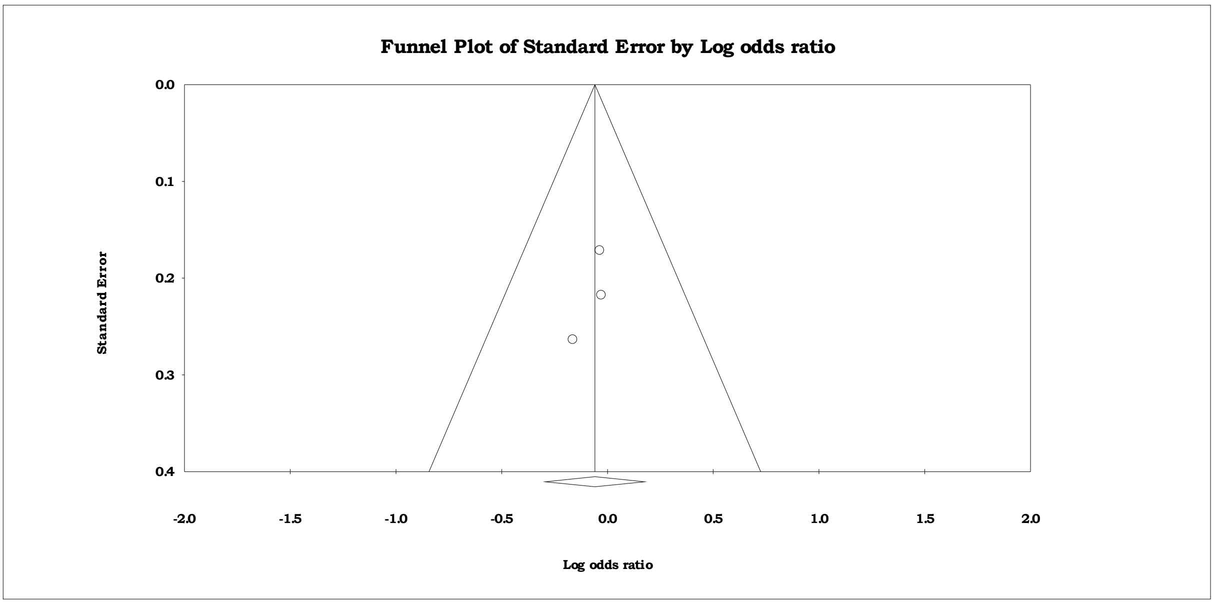

Funnel plot of the allelic model in the Turkish population group in absence of heterogeneity

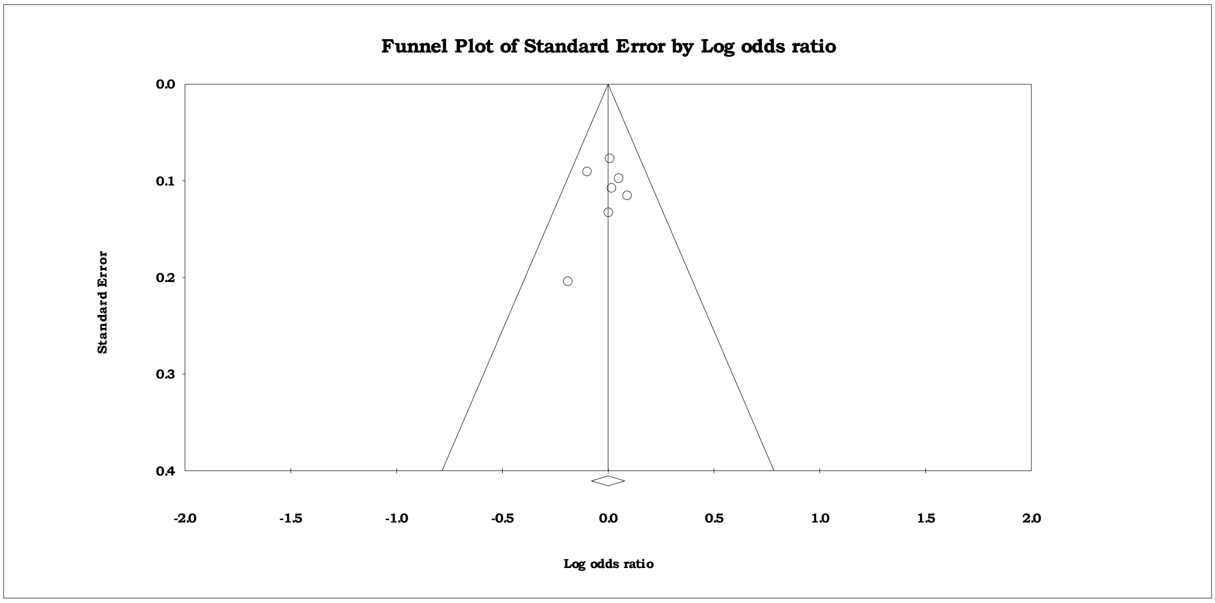

Funnel plot of the allelic model in the UK population group in absence of heterogeneity

EXCLI Journal 2019;18:331-355 – ISSN 1611-2156
Received: March 06, 2019, accepted: April 09, 2019, published: June 07, 2019

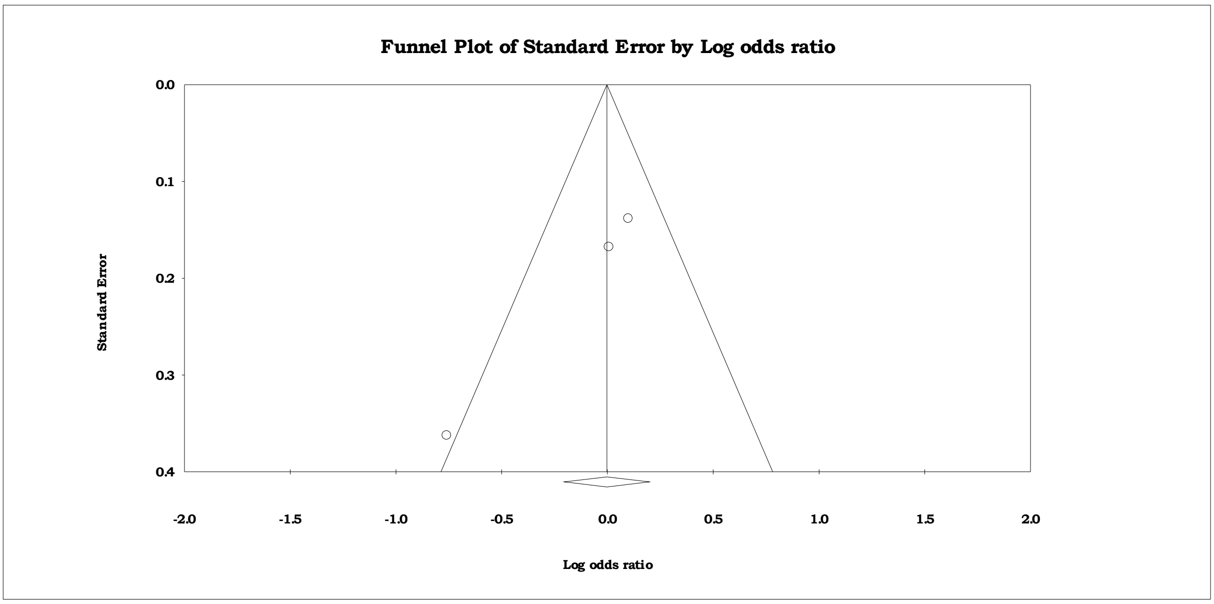

Funnel plot of the allelic model in the African population group with moderate heterogeneity

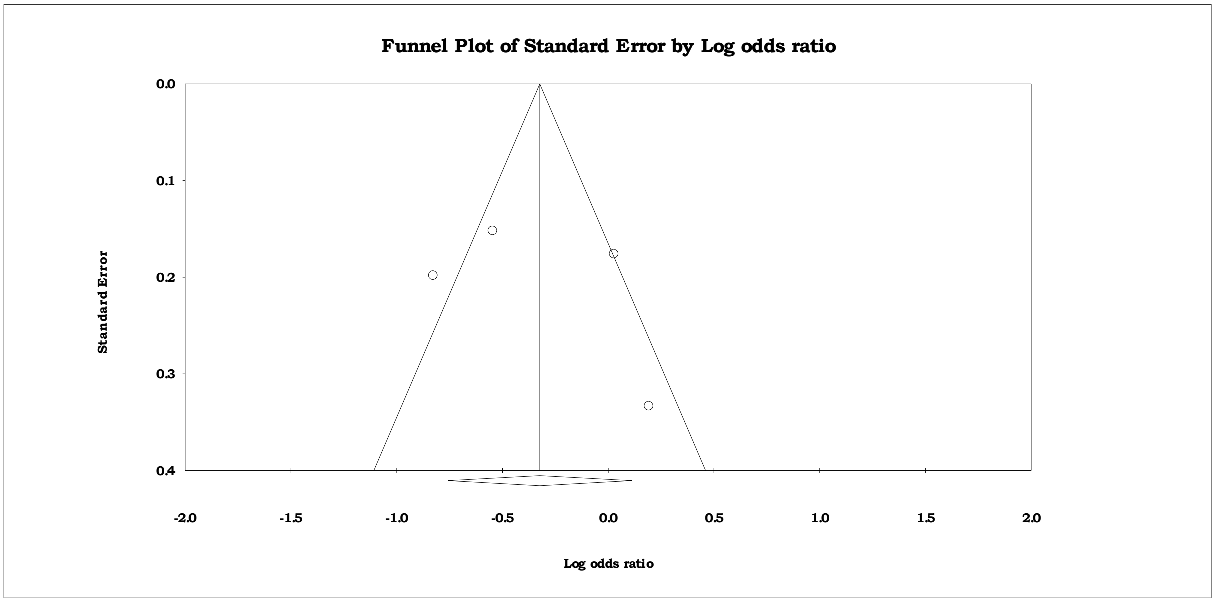

Funnel plot of the allelic model in the PAOD population group with high heterogeneity

EXCLI Journal 2019;18:331-355 – ISSN 1611-2156
Received: March 06, 2019, accepted: April 09, 2019, published: June 07, 2019

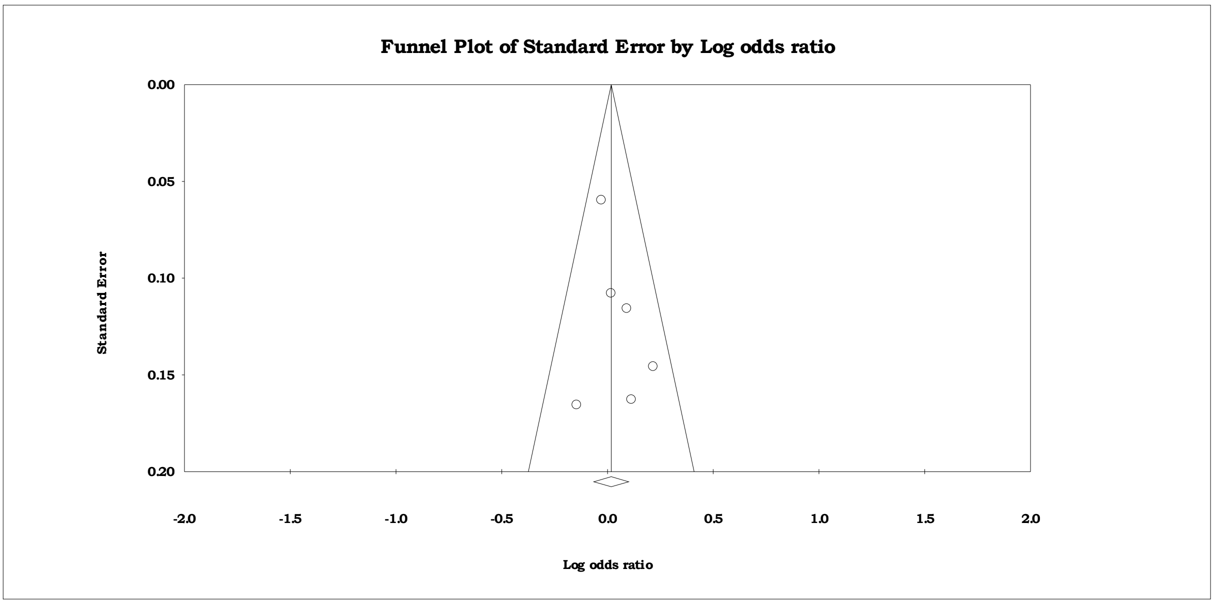

Funnel plot of the allelic model in the European population with CAD in absence of heterogeneity

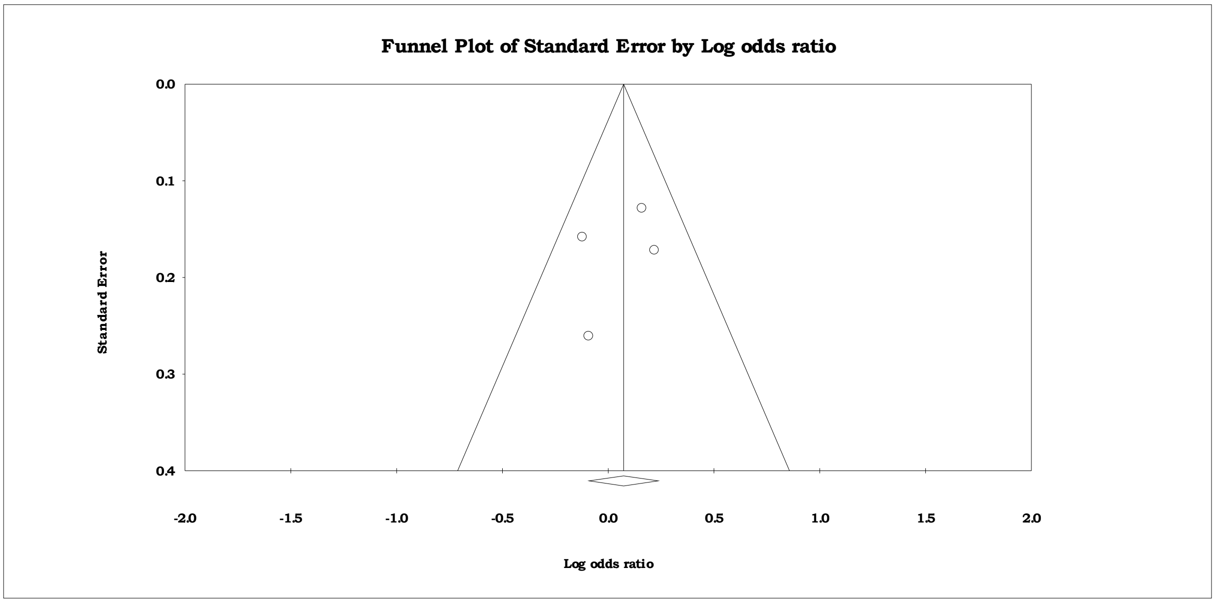

Funnel plot of the allelic model in the European population with IS in absence of heterogeneity

EXCLI Journal 2019;18:331-355 – ISSN 1611-2156
Received: March 06, 2019, accepted: April 09, 2019, published: June 07, 2019

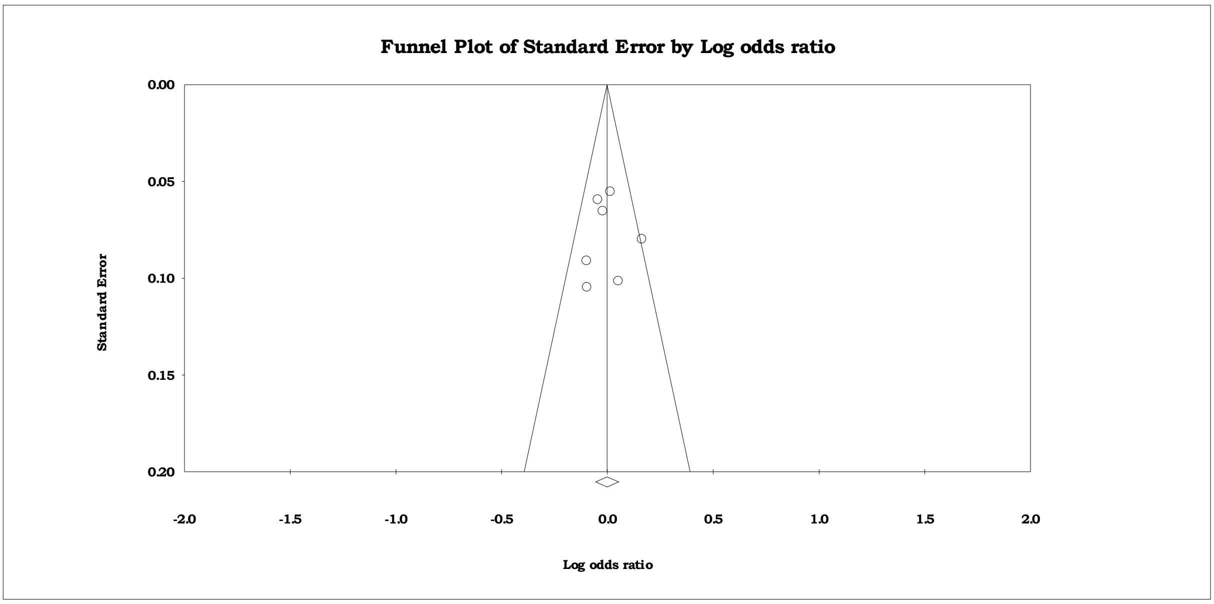

Funnel plot of the allelic model in the European population with MI in absence of heterogeneity

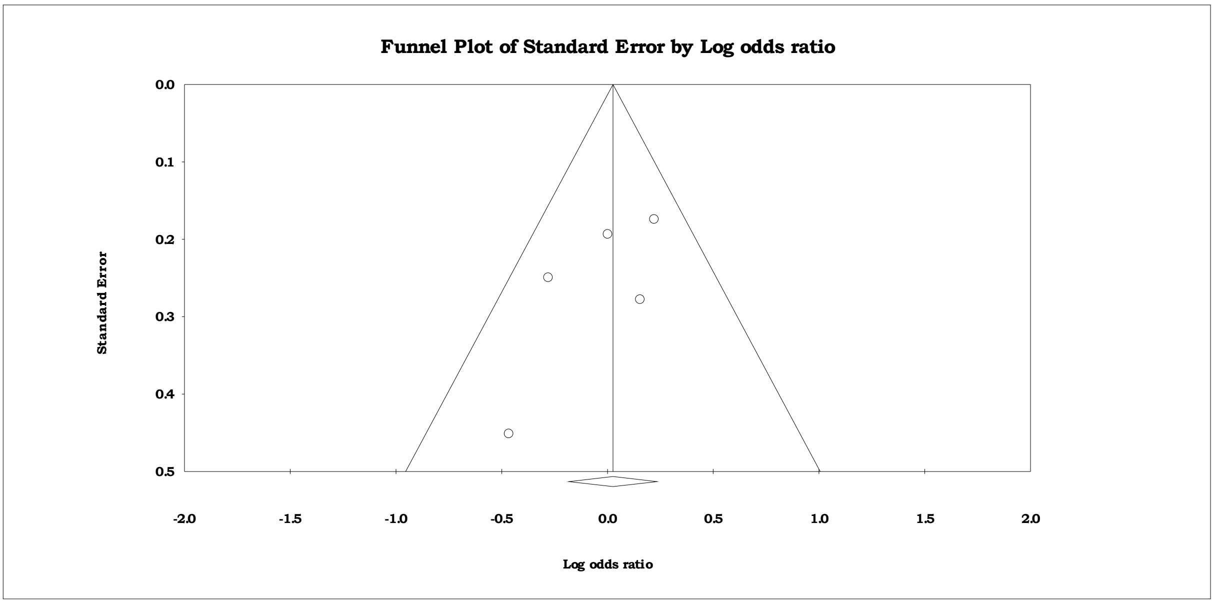

Funnel plot of the allelic model in the Indian population with CAD in absence of heterogeneity

EXCLI Journal 2019;18:331-355 – ISSN 1611-2156
Received: March 06, 2019, accepted: April 09, 2019, published: June 07, 2019

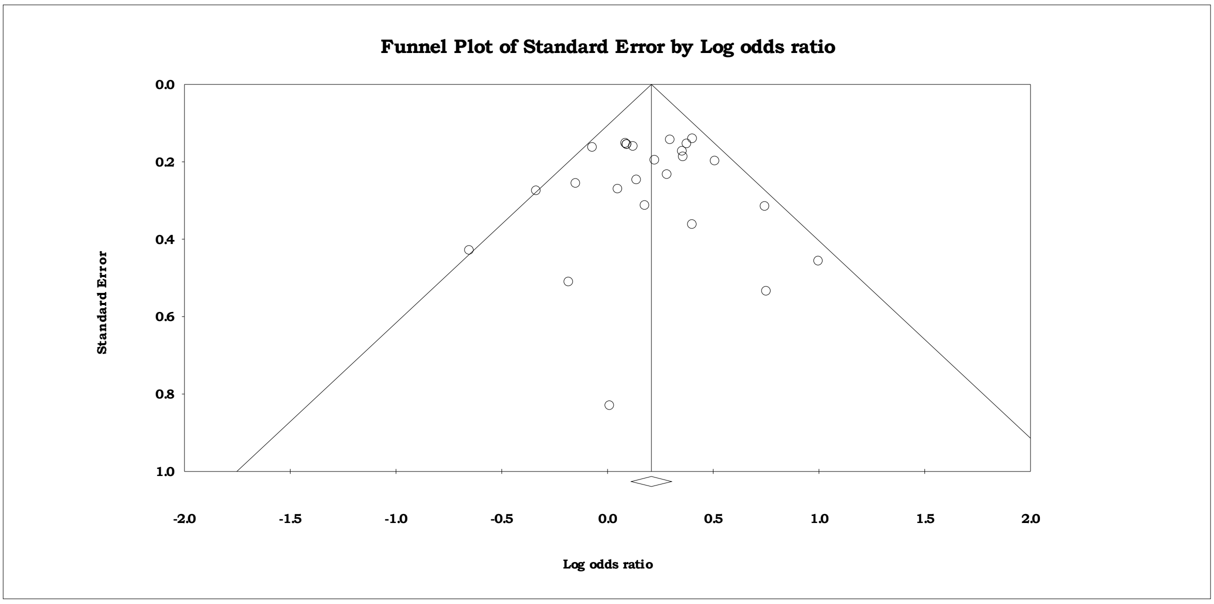

Funnel plot of the dominant model in the CAD population group in absence of heterogeneity

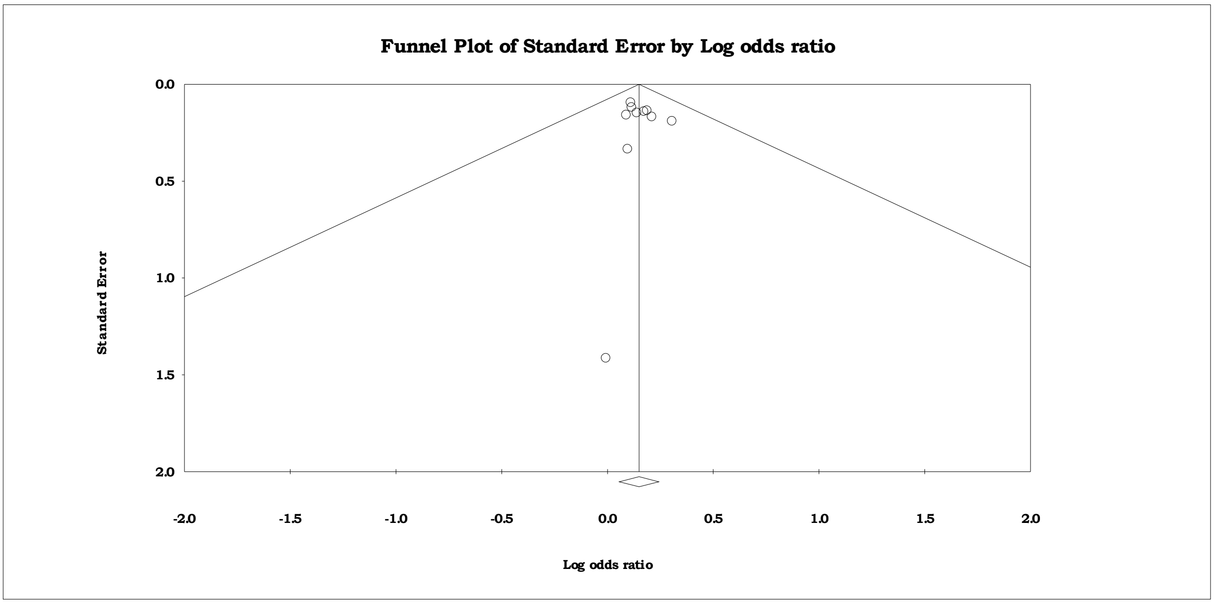

Funnel plot of the dominant model in the Chinese population group in absence of heterogeneity

EXCLI Journal 2019;18:331-355 – ISSN 1611-2156
Received: March 06, 2019, accepted: April 09, 2019, published: June 07, 2019

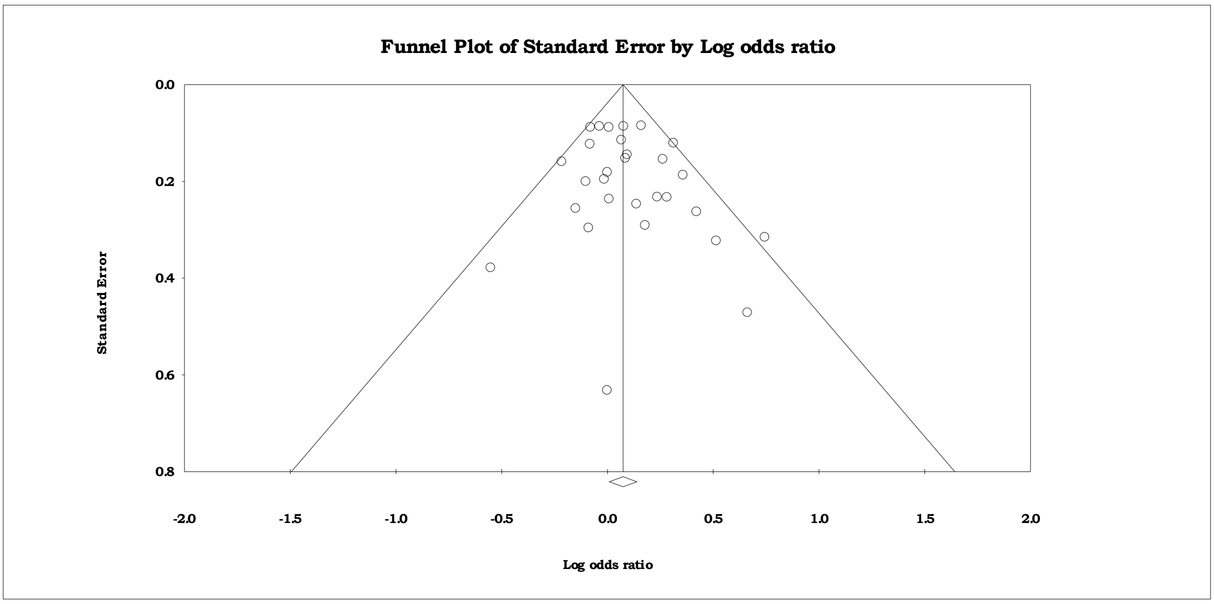

Funnel plot of the dominant model in the European population group in absence of heterogeneity

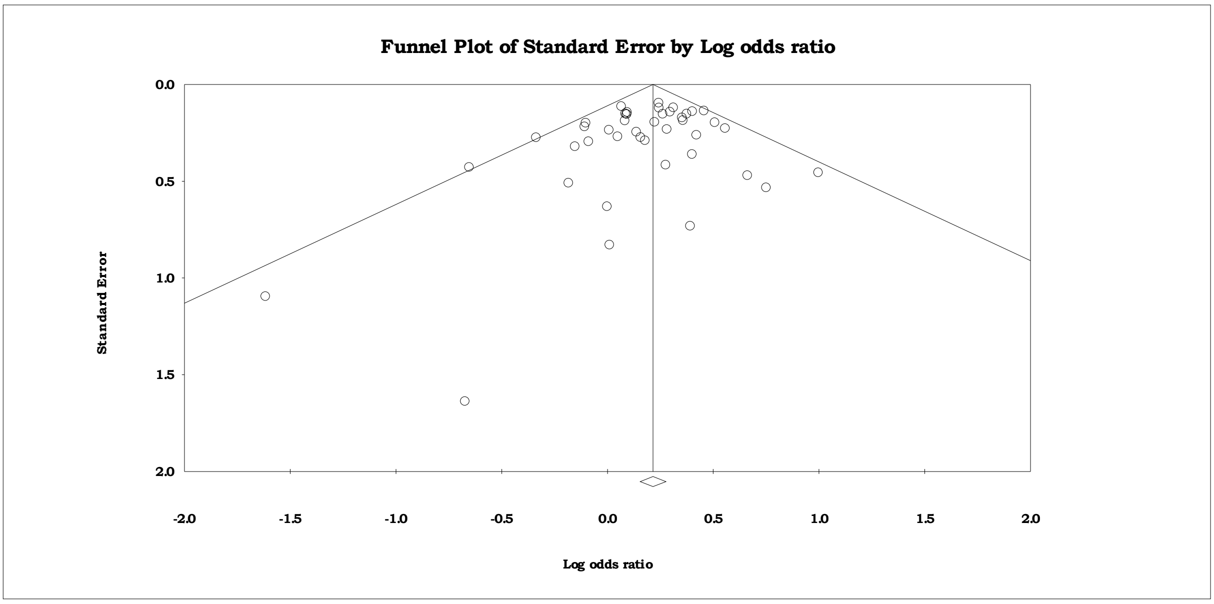

Funnel plot of the dominant model with healthy subjects as control group in absence of heterogeneity

EXCLI Journal 2019;18:331-355 – ISSN 1611-2156
Received: March 06, 2019, accepted: April 09, 2019, published: June 07, 2019

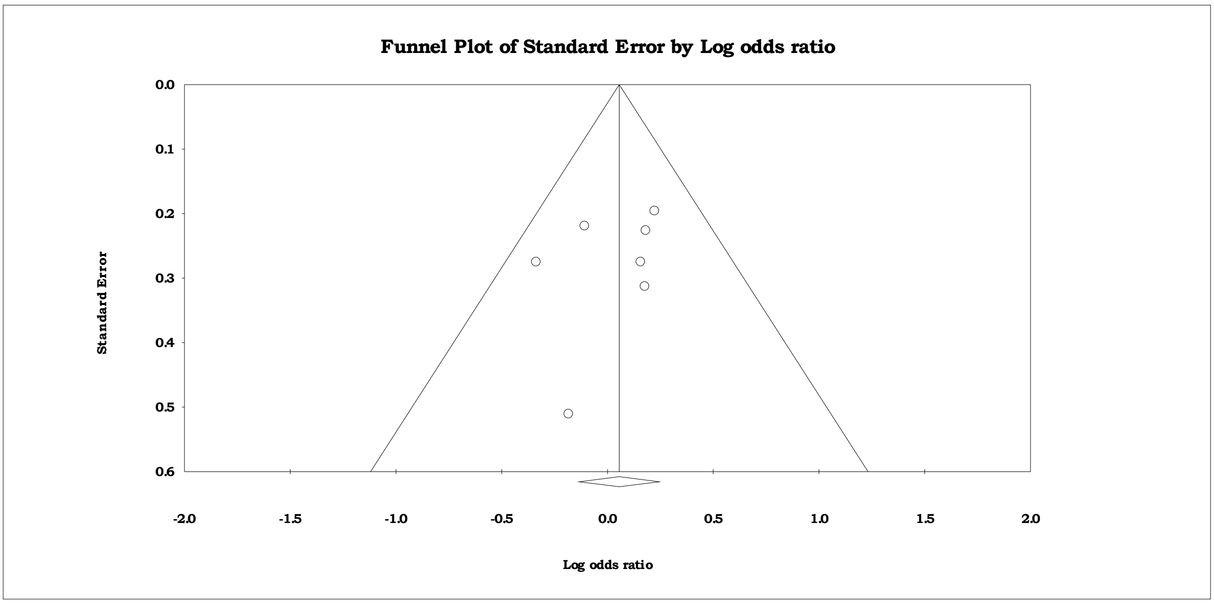

Funnel plot of the dominant model in the Indian population group in absence of heterogeneity

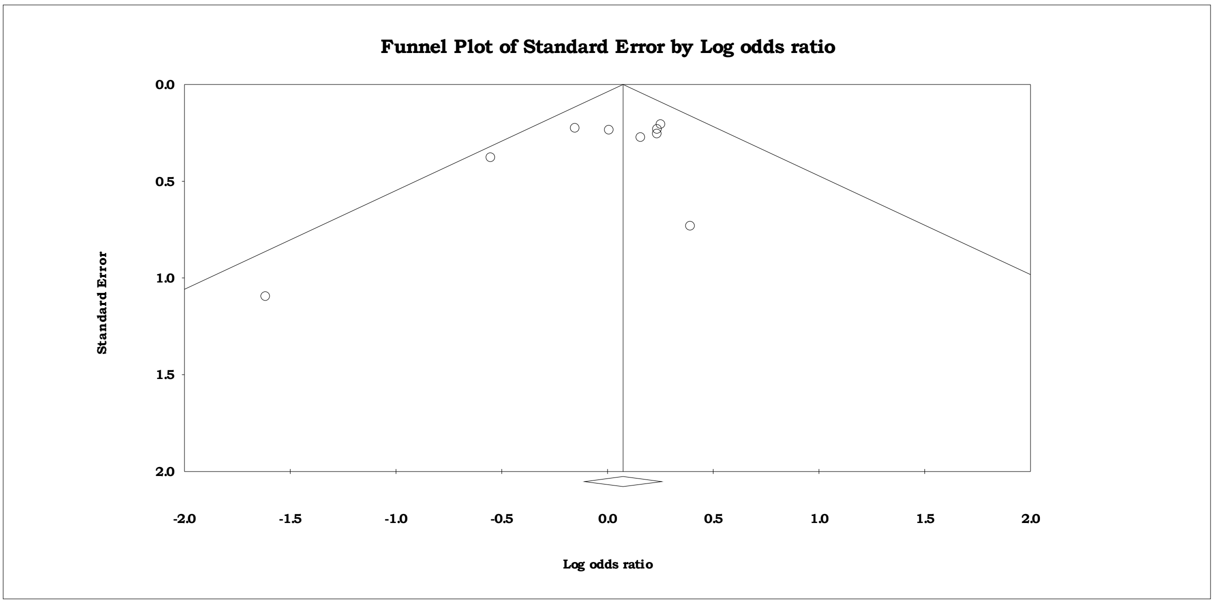

Funnel plot of the dominant model in the IS population group in absence of heterogeneity

EXCLI Journal 2019;18:331-355 – ISSN 1611-2156
Received: March 06, 2019, accepted: April 09, 2019, published: June 07, 2019

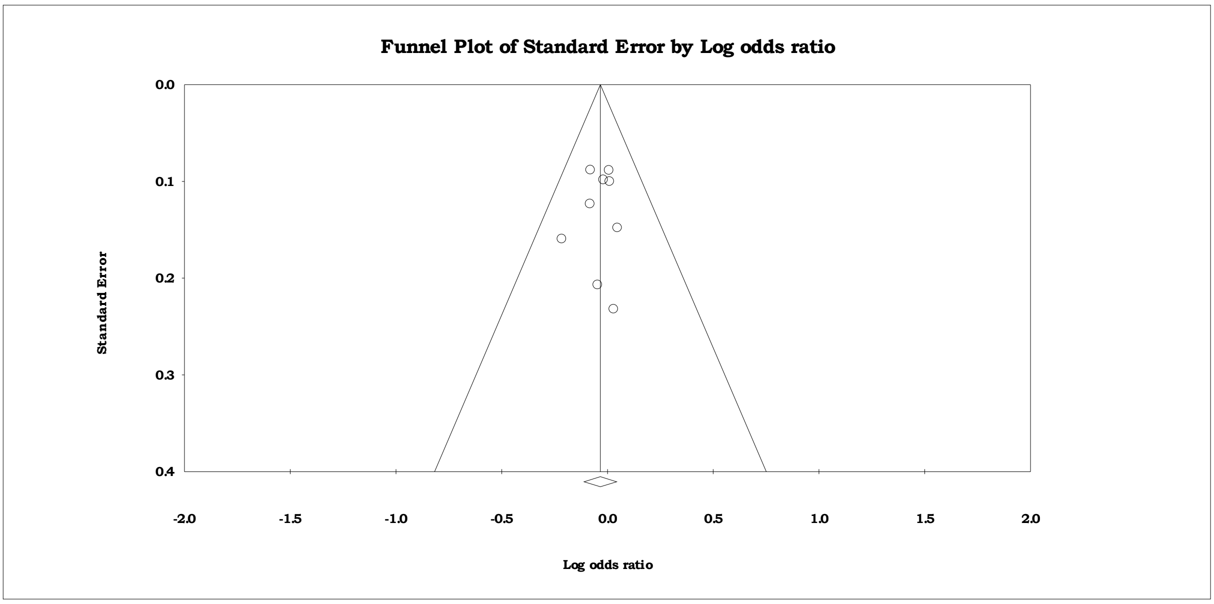

Funnel plot of the dominant model in the MI population group in absence of heterogeneity

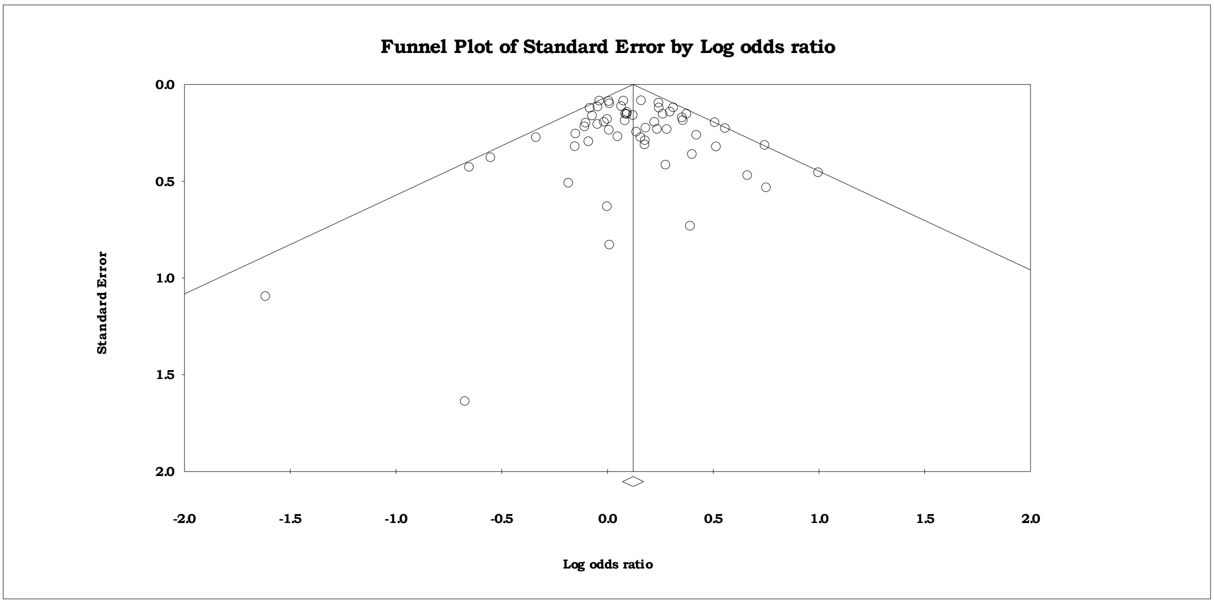

Funnel plot of the dominant model in the Overall population group

EXCLI Journal 2019;18:331-355 – ISSN 1611-2156
Received: March 06, 2019, accepted: April 09, 2019, published: June 07, 2019

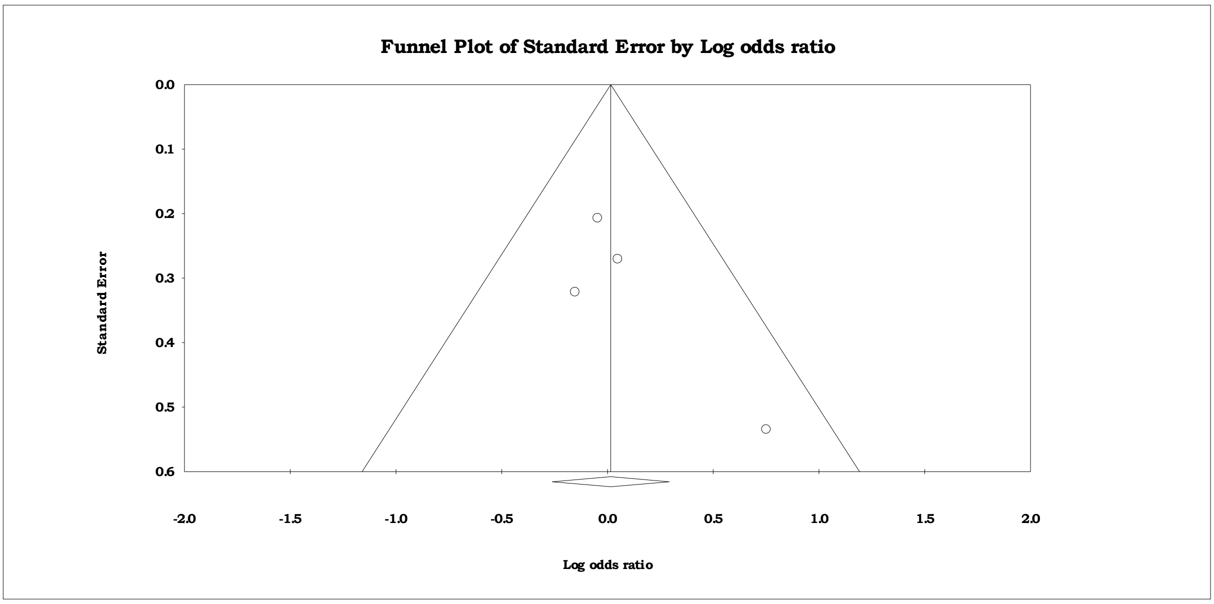

Funnel plot of the dominant model in the Turkish population group in absence of heterogeneity

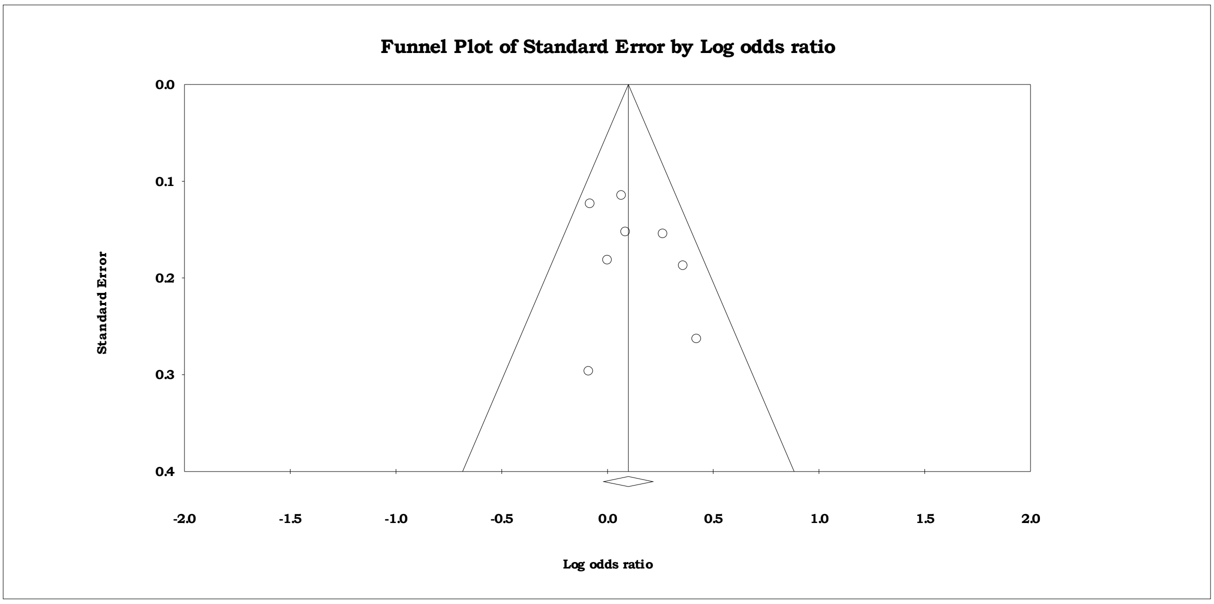

Funnel plot of the dominant model in the UK population group in absence of heterogeneity

EXCLI Journal 2019;18:331-355 – ISSN 1611-2156
Received: March 06, 2019, accepted: April 09, 2019, published: June 07, 2019

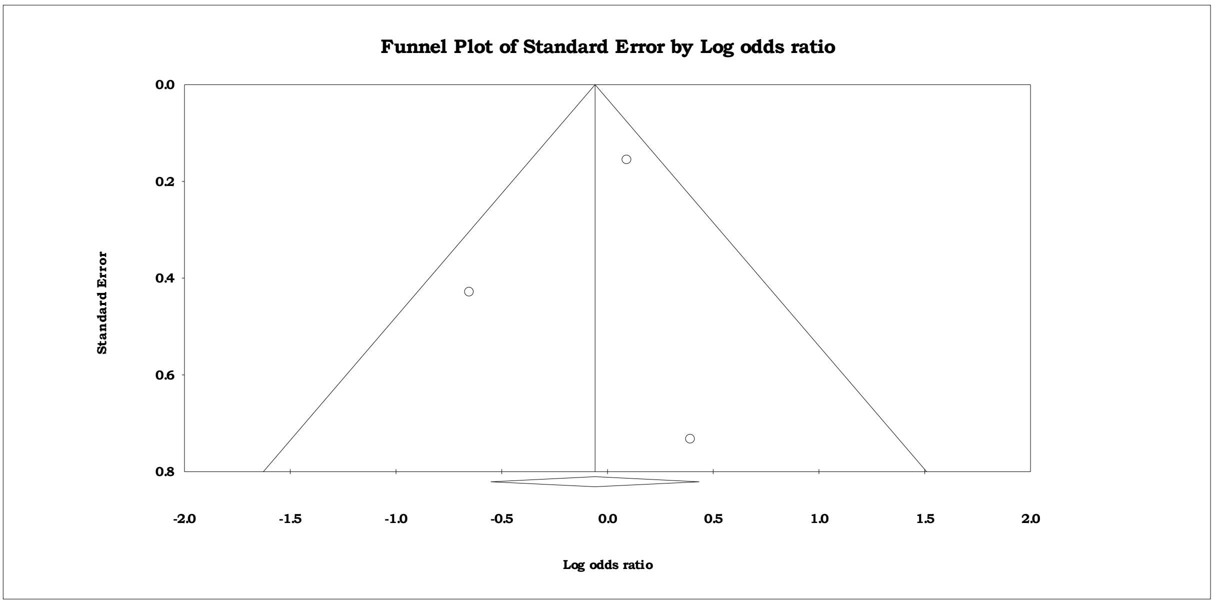

Funnel plot of the dominant model in the African population group with low heterogeneity

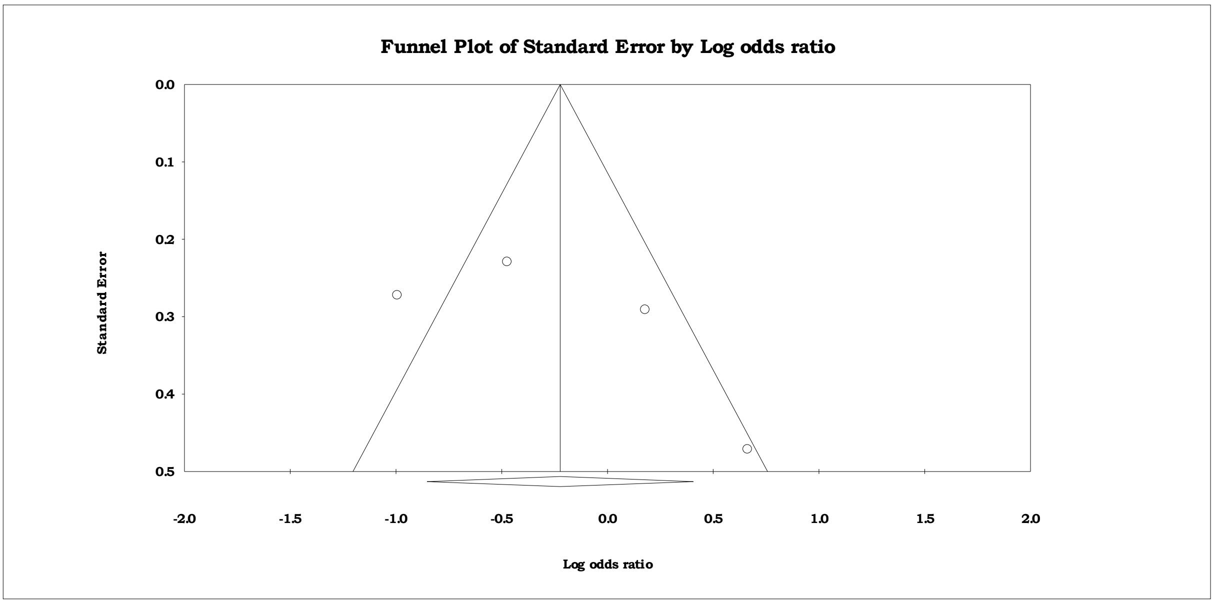

Funnel plot of the dominant model in the PAOD population group with high heterogeneity

EXCLI Journal 2019;18:331-355 – ISSN 1611-2156
Received: March 06, 2019, accepted: April 09, 2019, published: June 07, 2019

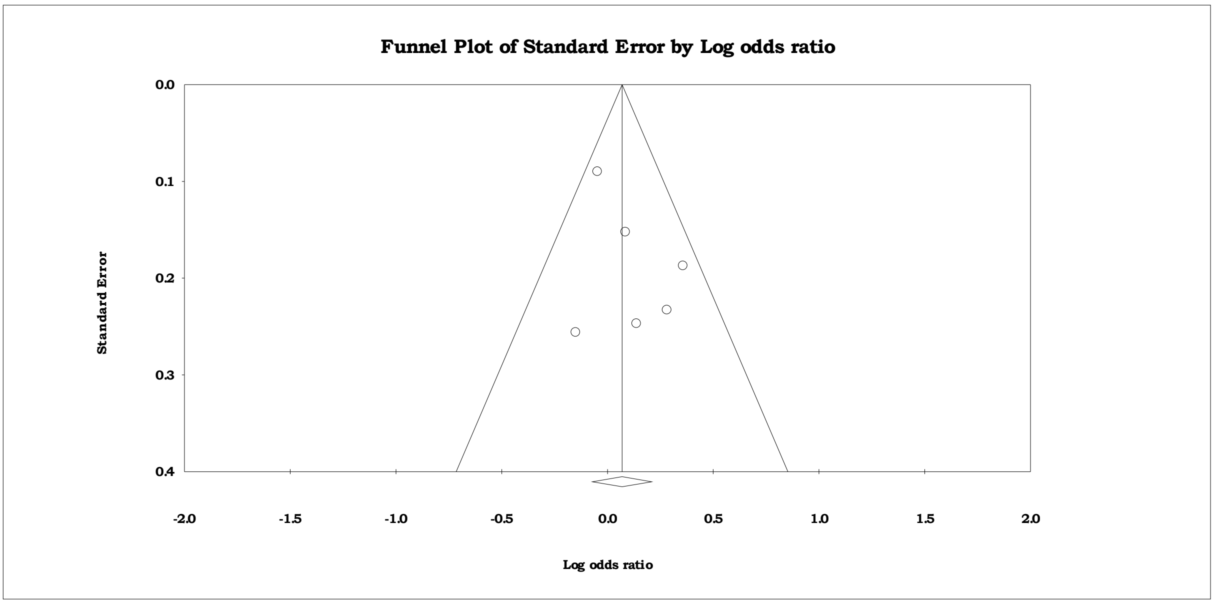

Funnel plot of the dominant model in the European population with CAD in absence of heterogeneity

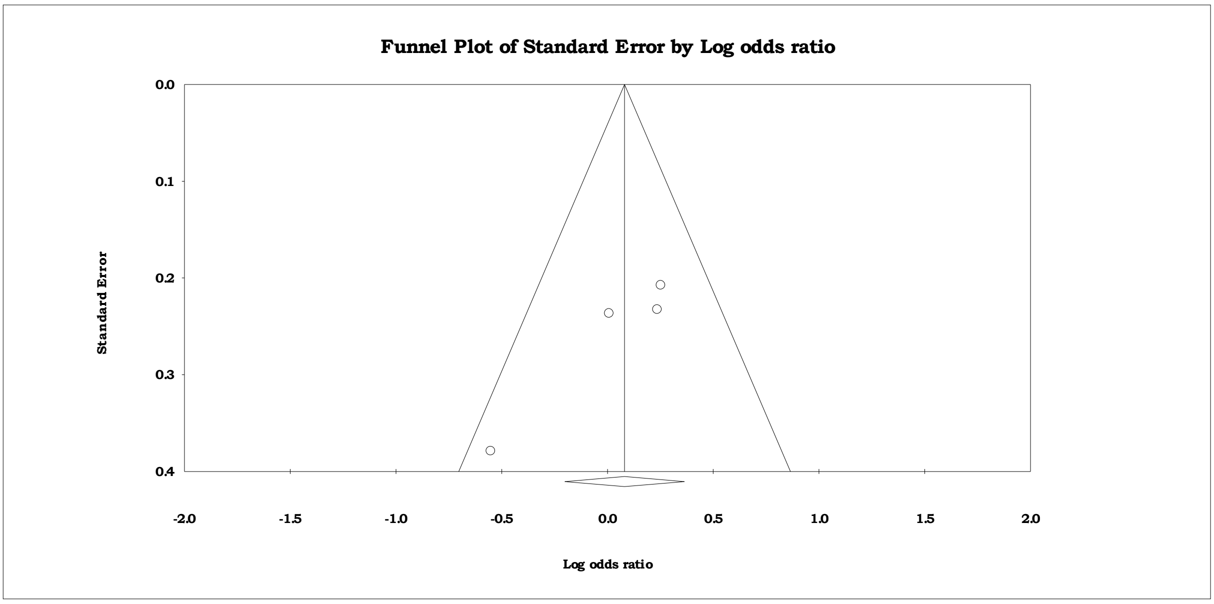

Funnel plot of the dominant model in the European population with IS in absence of heterogeneity

EXCLI Journal 2019;18:331-355 – ISSN 1611-2156
Received: March 06, 2019, accepted: April 09, 2019, published: June 07, 2019

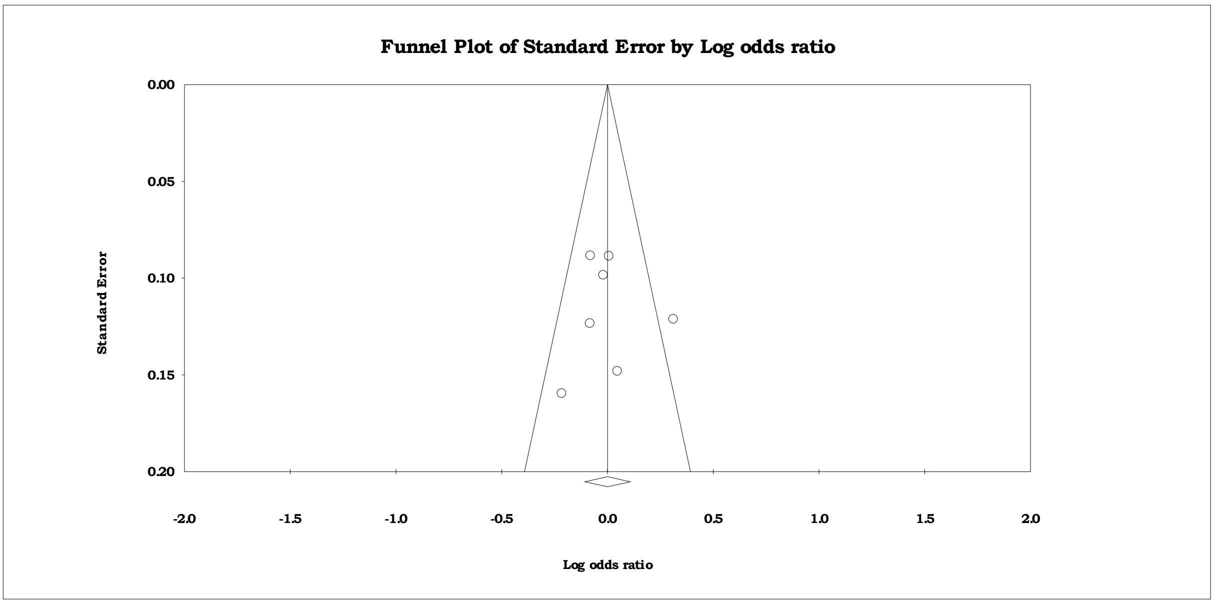

Funnel plot of the dominant model in the European population with MI in absence of heterogeneity

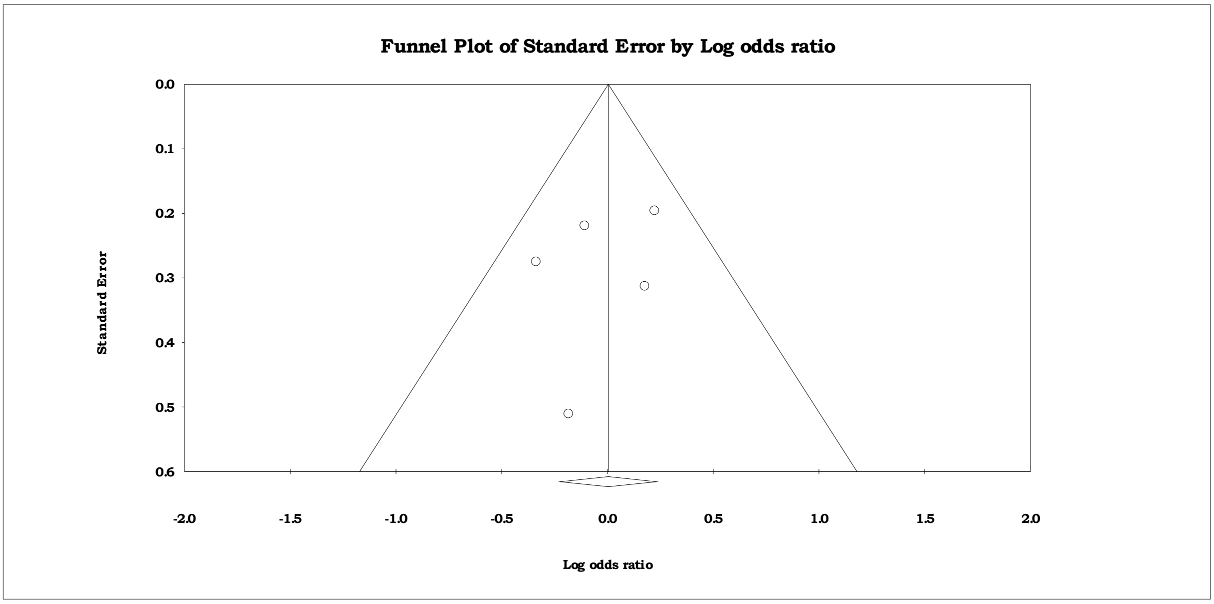

Funnel plot of the dominant model in the Indian population with CAD in absence of heterogeneity

EXCLI Journal 2019;18:331-355 – ISSN 1611-2156
Received: March 06, 2019, accepted: April 09, 2019, published: June 07, 2019

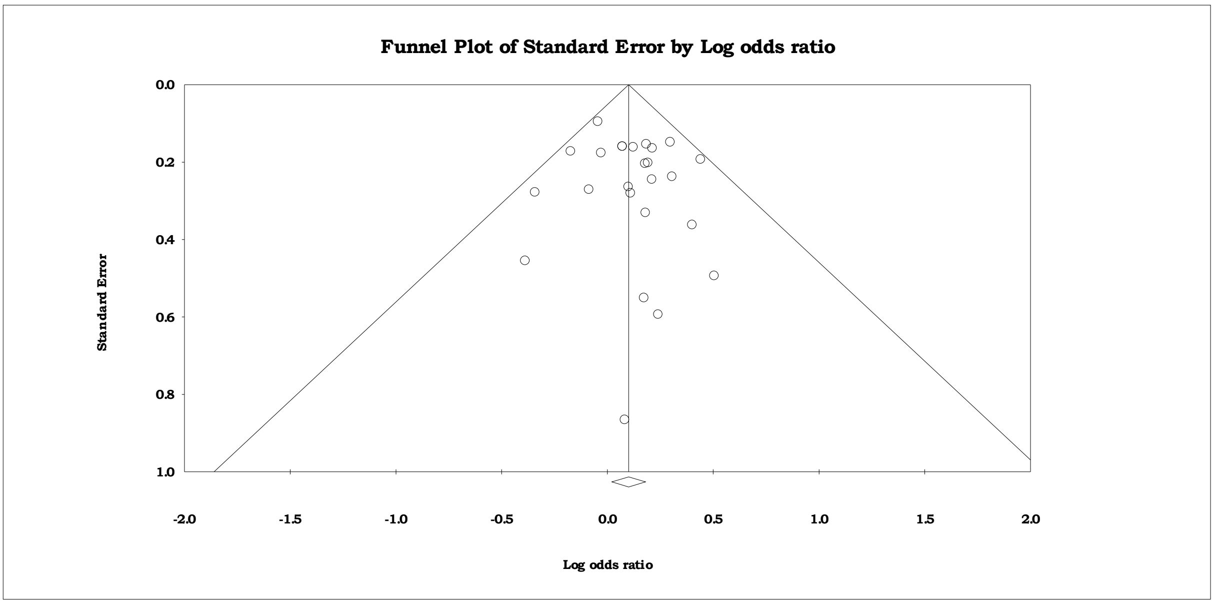

Funnel plot of the heterozygous model in the CAD population group in absence of heterogeneity

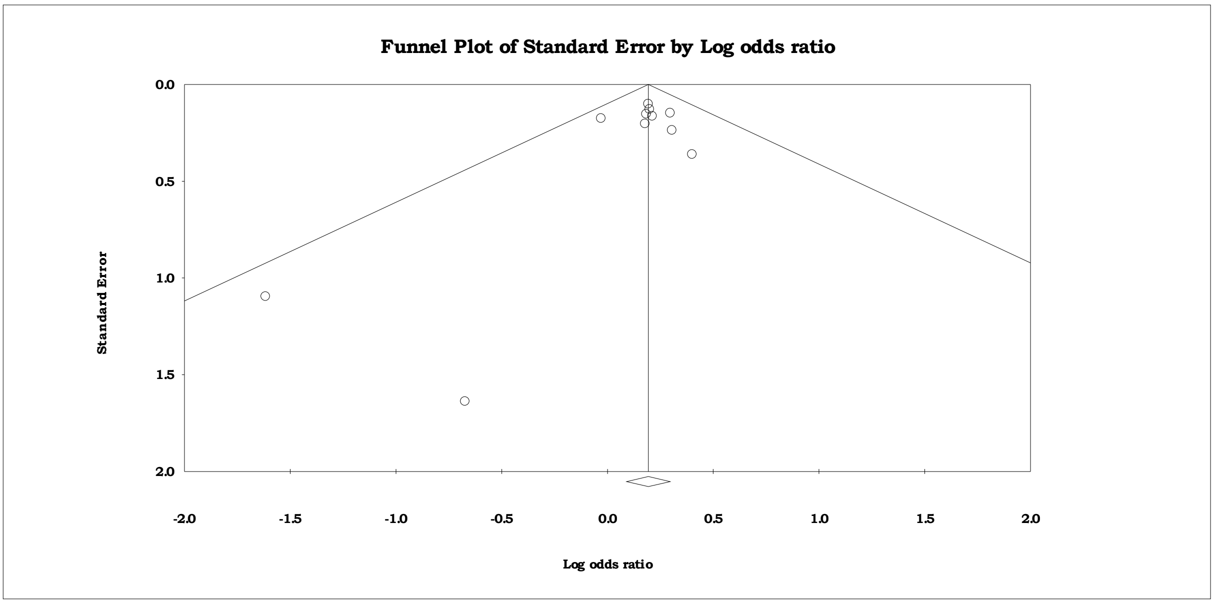

Funnel plot of the heterozygous model in the Chinese population group in absence of heterogeneity

EXCLI Journal 2019;18:331-355 – ISSN 1611-2156
Received: March 06, 2019, accepted: April 09, 2019, published: June 07, 2019

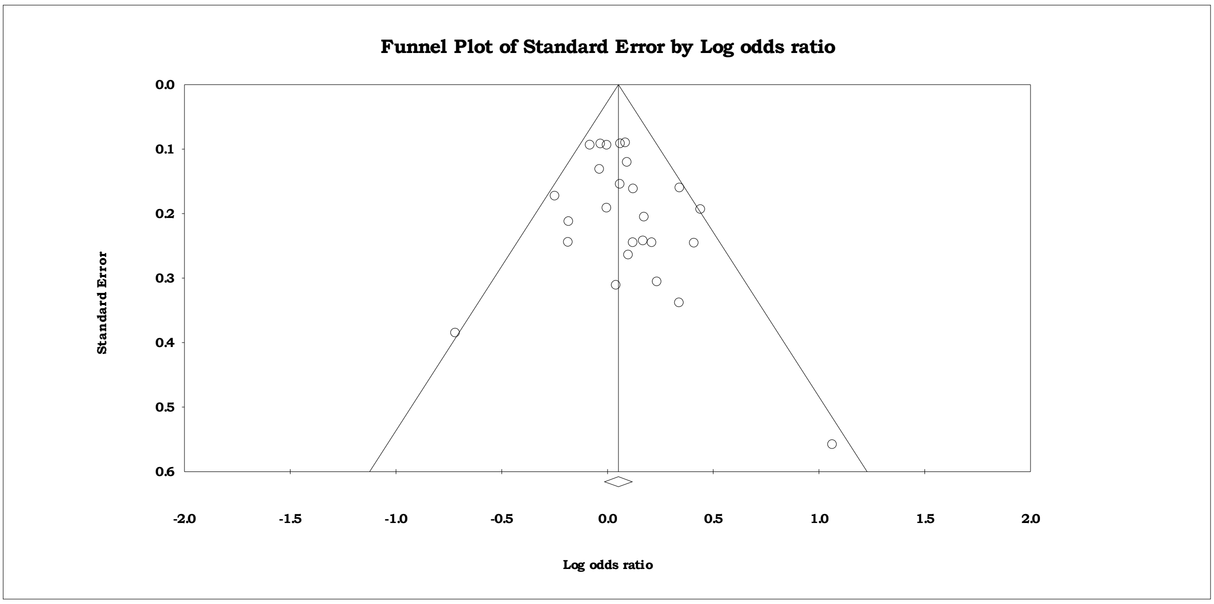

Funnel plot of the heterozygous model in the European population group in absence of heterogeneity

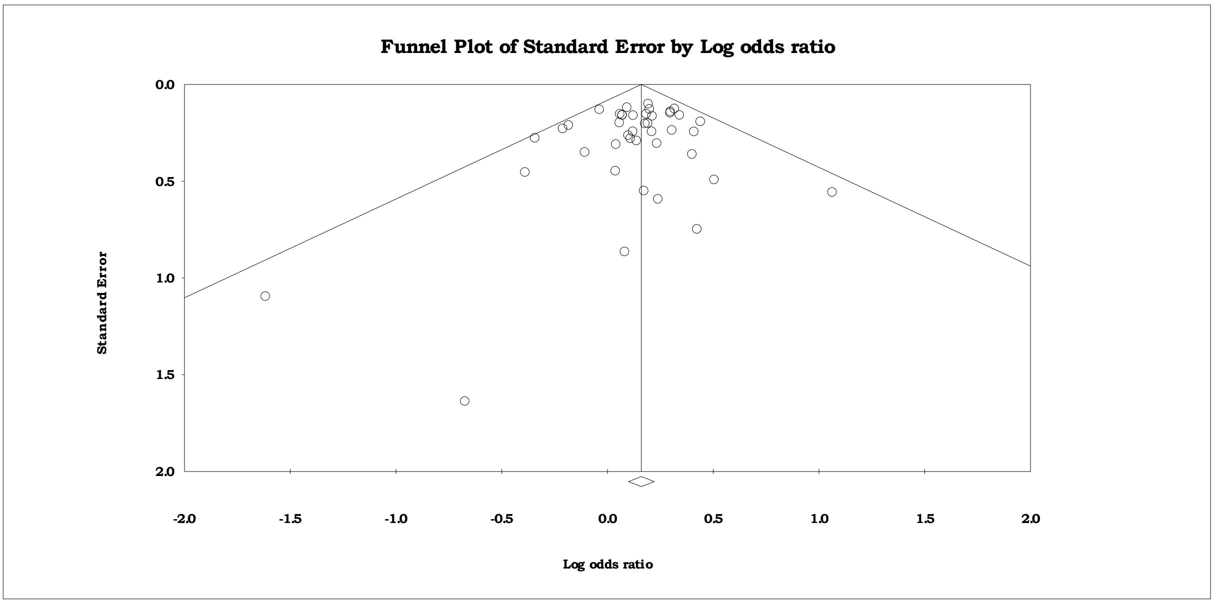

Funnel plot of the heterozygous model with healthy subjects as control group in absence of heterogeneity

EXCLI Journal 2019;18:331-355 – ISSN 1611-2156
Received: March 06, 2019, accepted: April 09, 2019, published: June 07, 2019

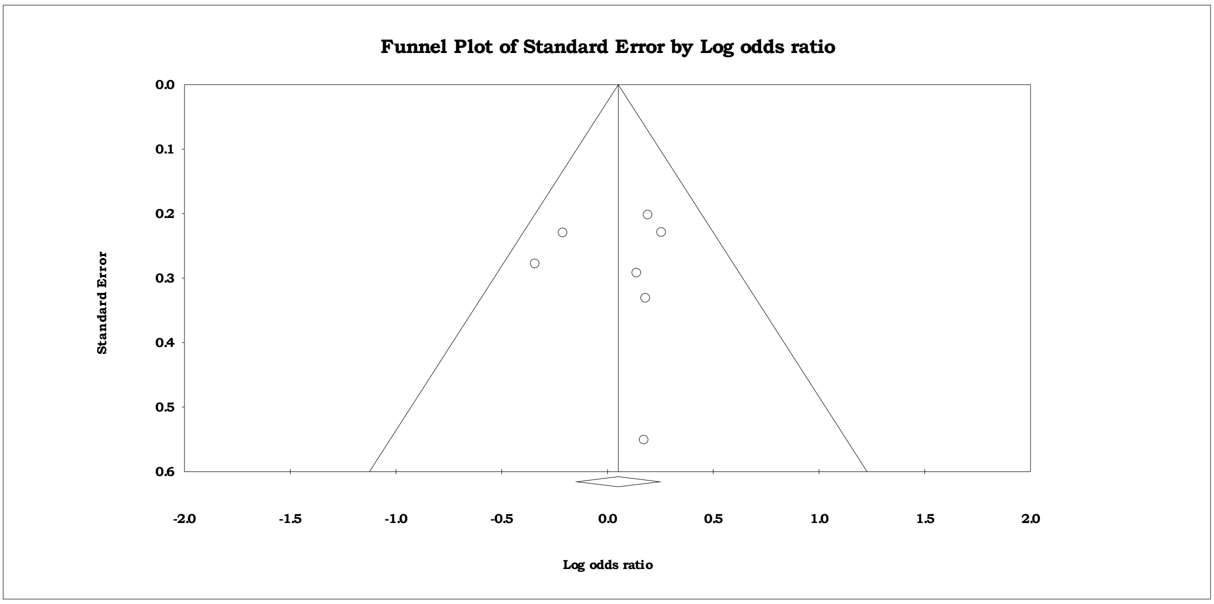

Funnel plot of the heterozygous model in the Indian population group in absence of heterogeneity

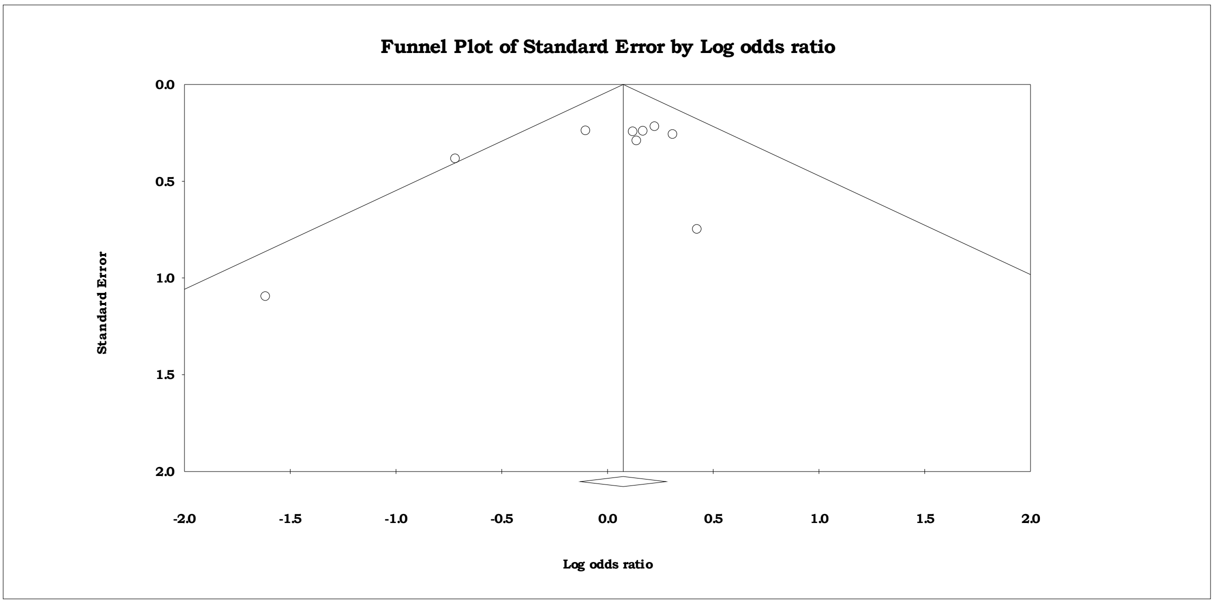

Funnel plot of the heterozygous model in the IS population group in absence of heterogeneity

EXCLI Journal 2019;18:331-355 – ISSN 1611-2156
Received: March 06, 2019, accepted: April 09, 2019, published: June 07, 2019

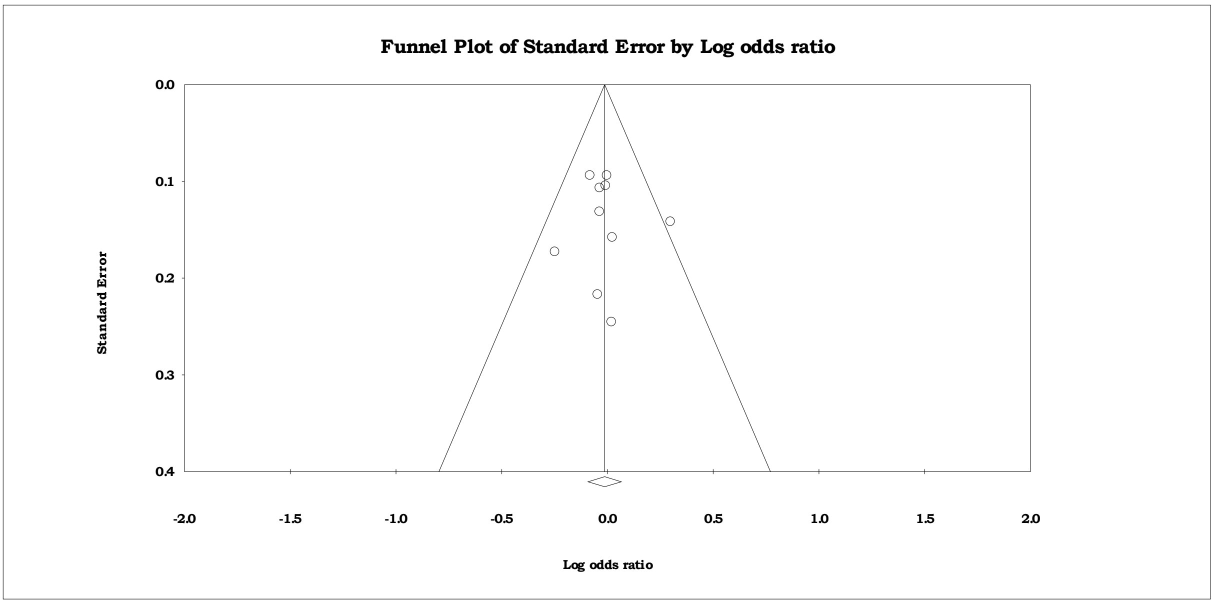

Funnel plot of the heterozygous model in the MI population group in absence of heterogeneity

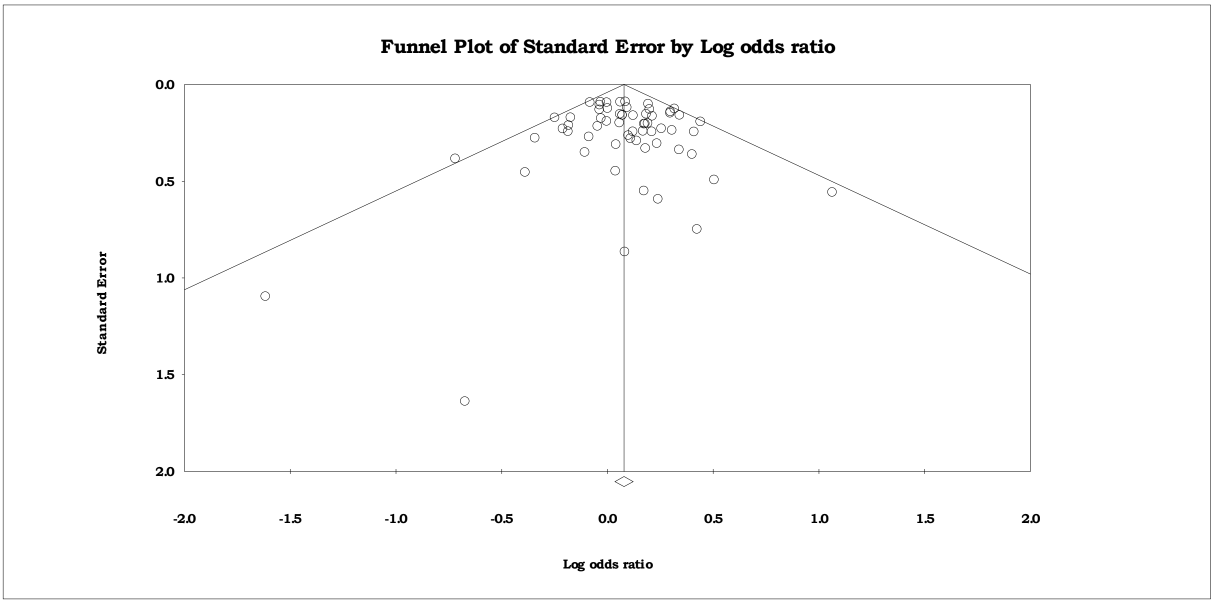

Funnel plot of the heterozygous model in the Overall population group in absence of heterogeneity

EXCLI Journal 2019;18:331-355 – ISSN 1611-2156
Received: March 06, 2019, accepted: April 09, 2019, published: June 07, 2019

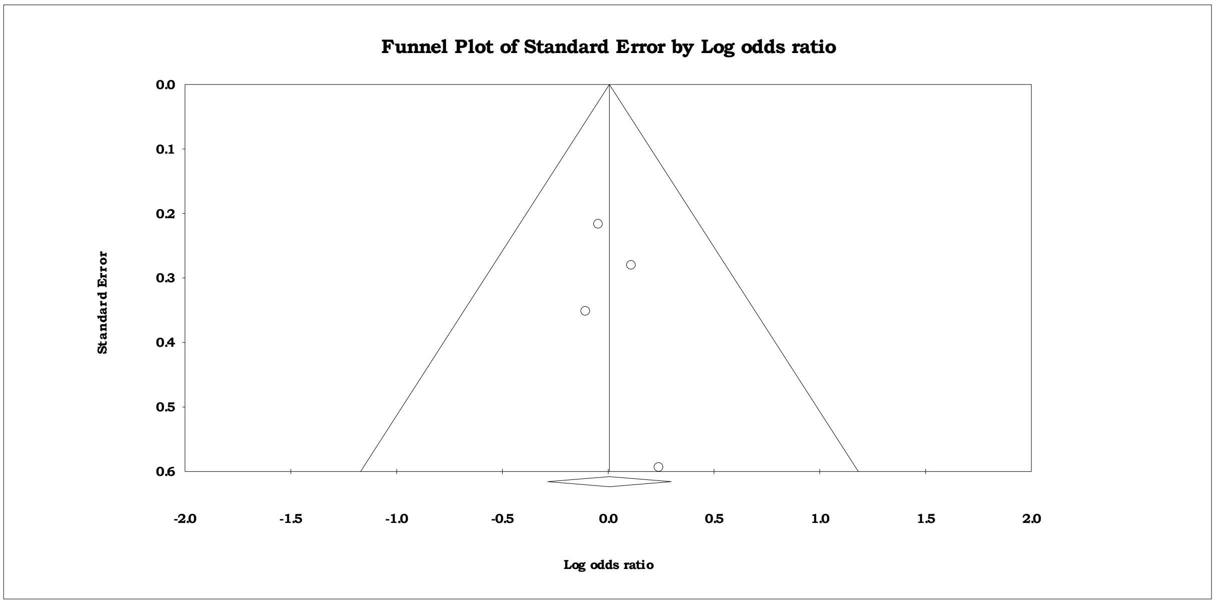

Funnel plot of the heterozygous model in the Turkish population group in absence of heterogeneity

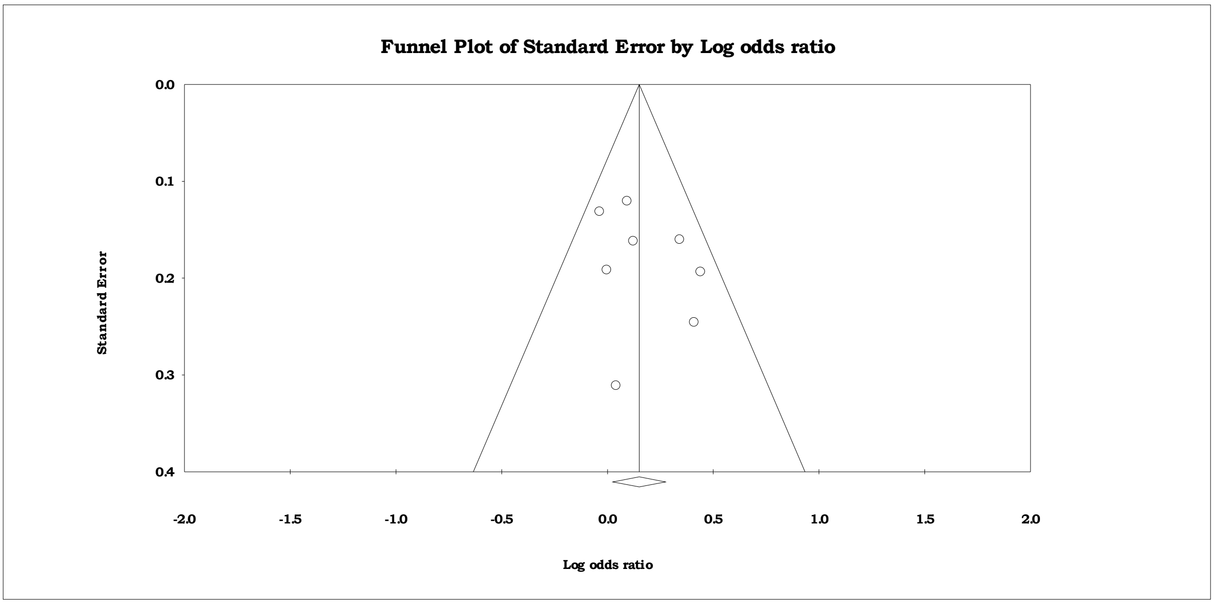

Funnel plot of the heterozygous model in the UK population group in absence of heterogeneity

EXCLI Journal 2019;18:331-355 – ISSN 1611-2156
Received: March 06, 2019, accepted: April 09, 2019, published: June 07, 2019

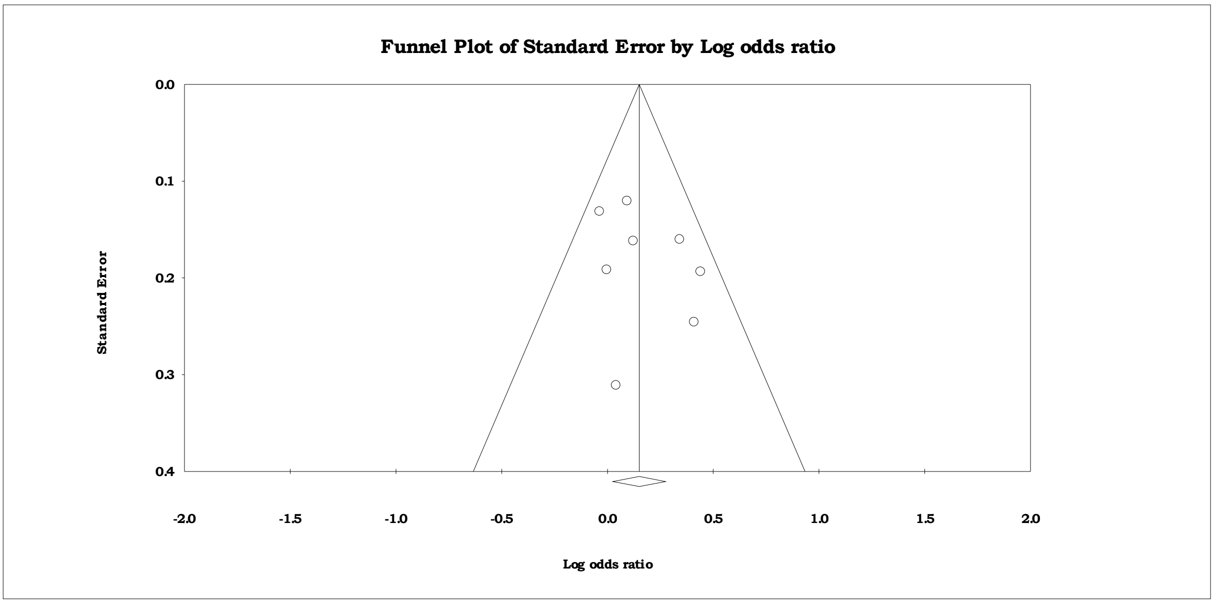

Funnel plot of the heterozygous model in the African population group in absence of heterogeneity

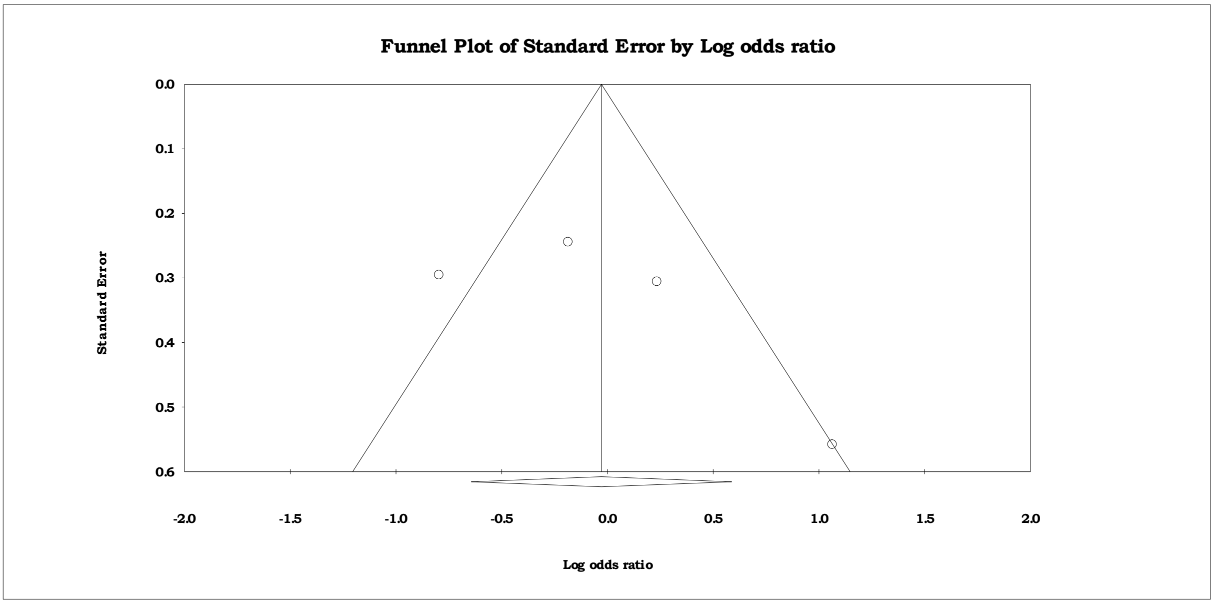

Funnel plot of the heterozygous model in the PAOD population group with high heterogeneity

EXCLI Journal 2019;18:331-355 – ISSN 1611-2156
Received: March 06, 2019, accepted: April 09, 2019, published: June 07, 2019

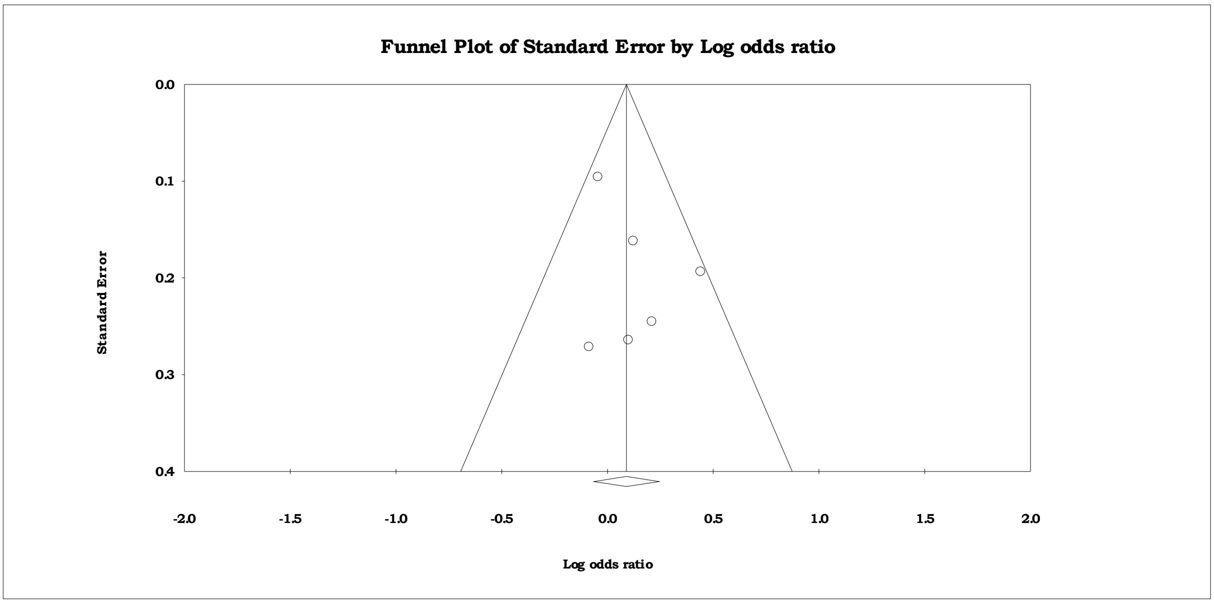

Funnel plot of the heterozygous model in the European population with CAD in absence of heterogeneity

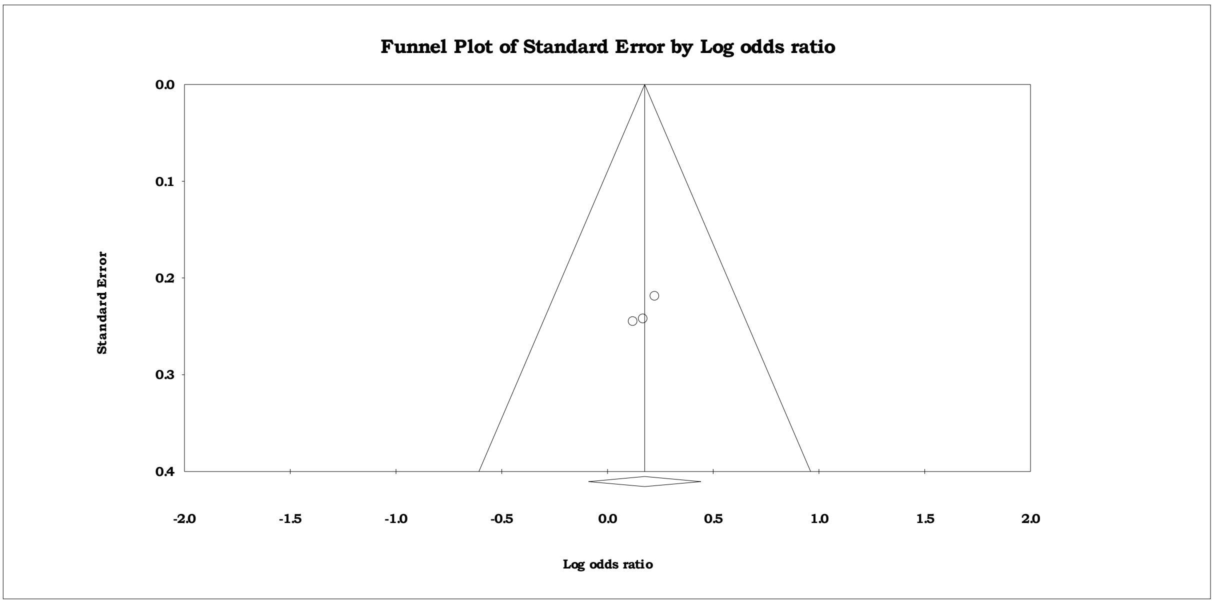

Funnel plot of the heterozygous model in the European population with IS in absence of heterogeneity

EXCLI Journal 2019;18:331-355 – ISSN 1611-2156
Received: March 06, 2019, accepted: April 09, 2019, published: June 07, 2019

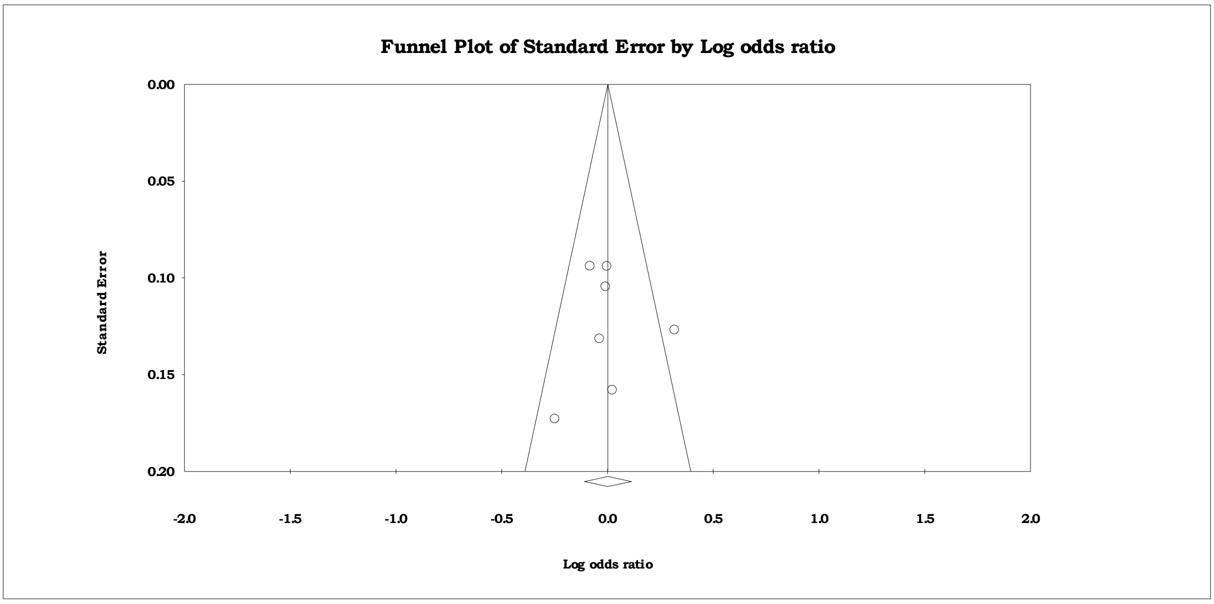

Funnel plot of the heterozygous model in the European population with MI in absence of heterogeneity

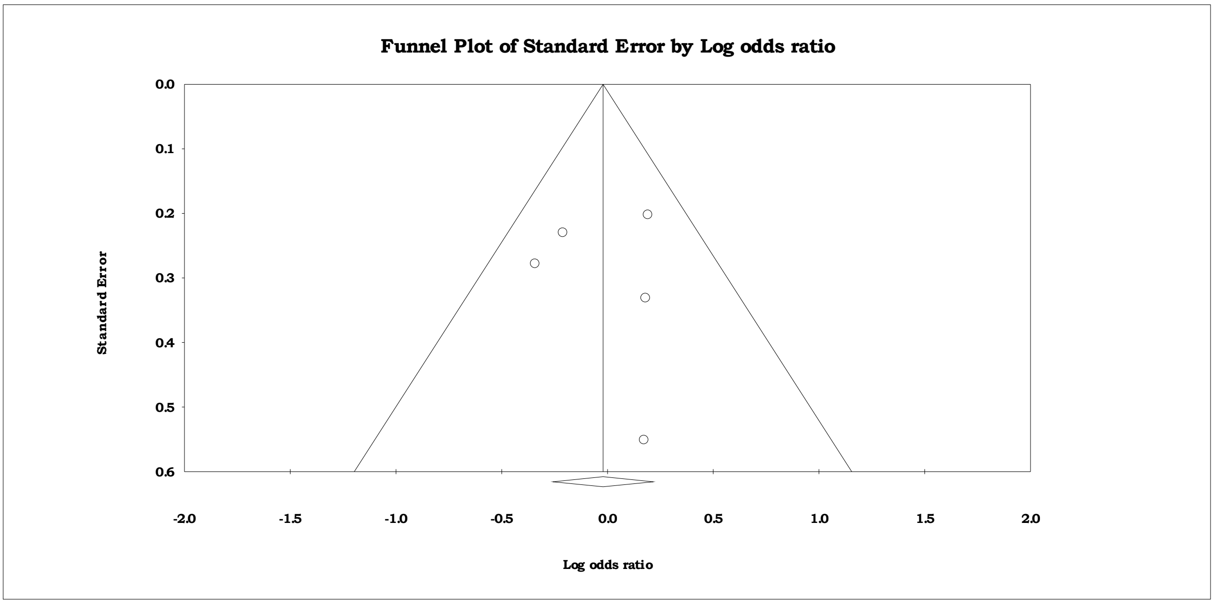

Funnel plot of the heterozygous model in the Indian population with CAD in absence of heterogeneity

EXCLI Journal 2019;18:331-355 – ISSN 1611-2156
Received: March 06, 2019, accepted: April 09, 2019, published: June 07, 2019

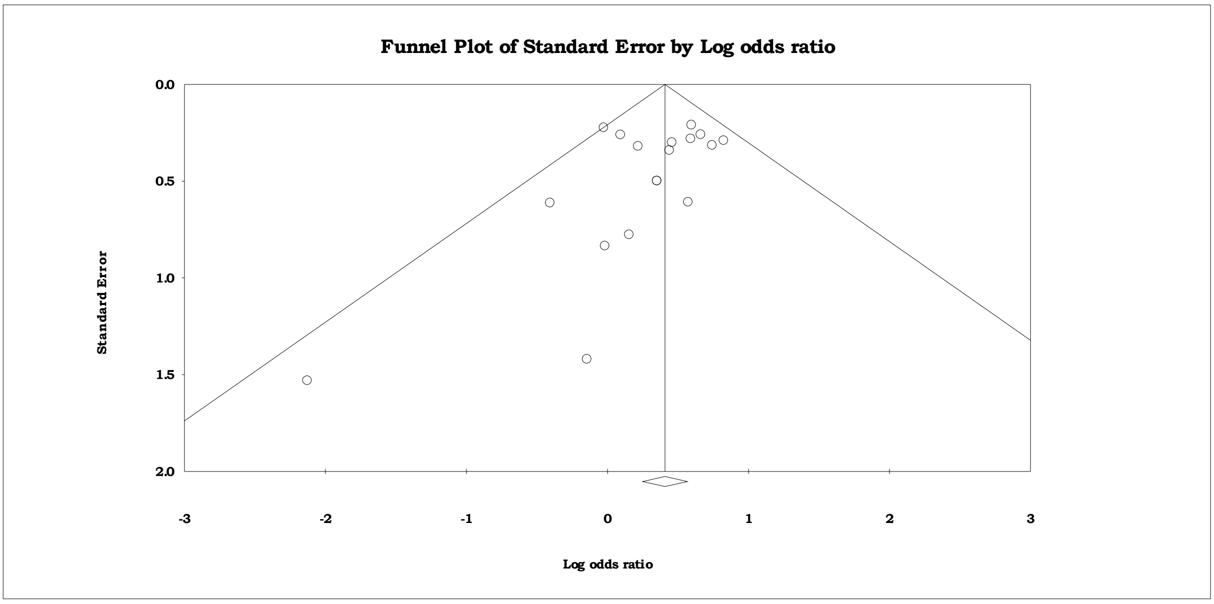

Funnel plot of the homozygous model in the CAD population group in absence of heterogeneity

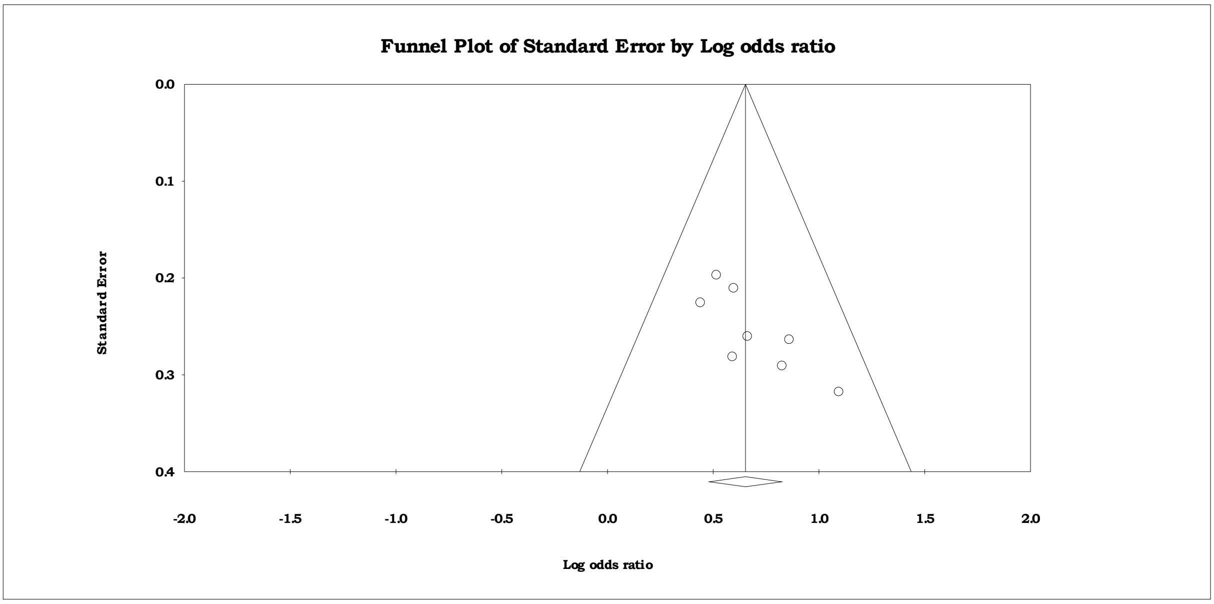

Funnel plot of the homozygous model in the Chinese population group in absence of heterogeneity

EXCLI Journal 2019;18:331-355 – ISSN 1611-2156
Received: March 06, 2019, accepted: April 09, 2019, published: June 07, 2019

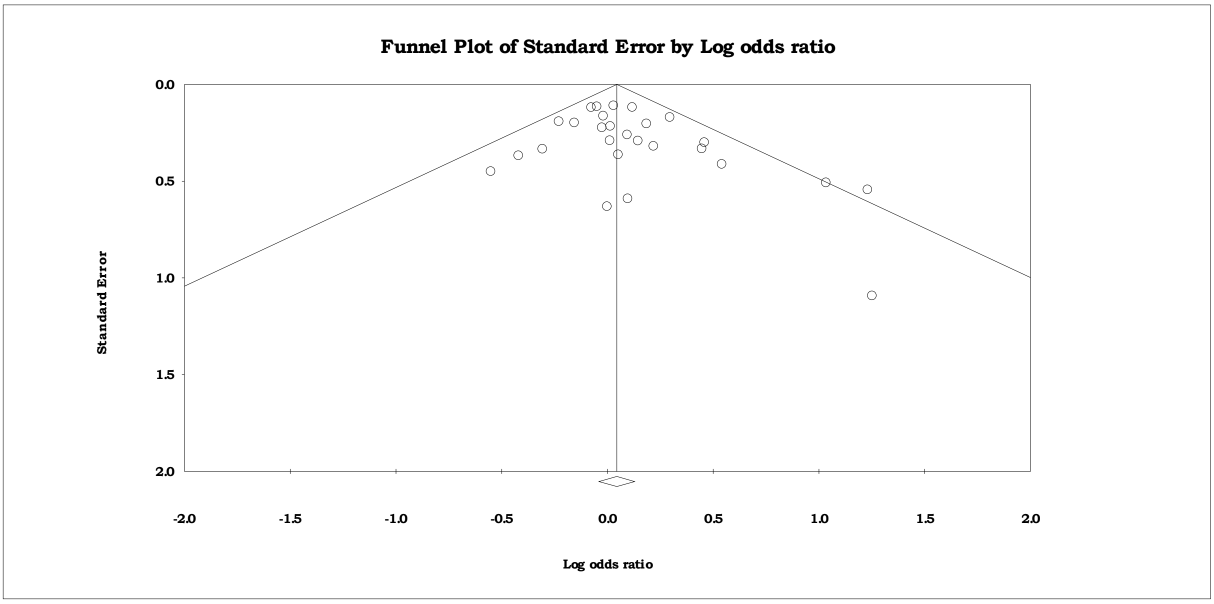

Funnel plot of the homozygous model in the European population group in absence of heterogeneity

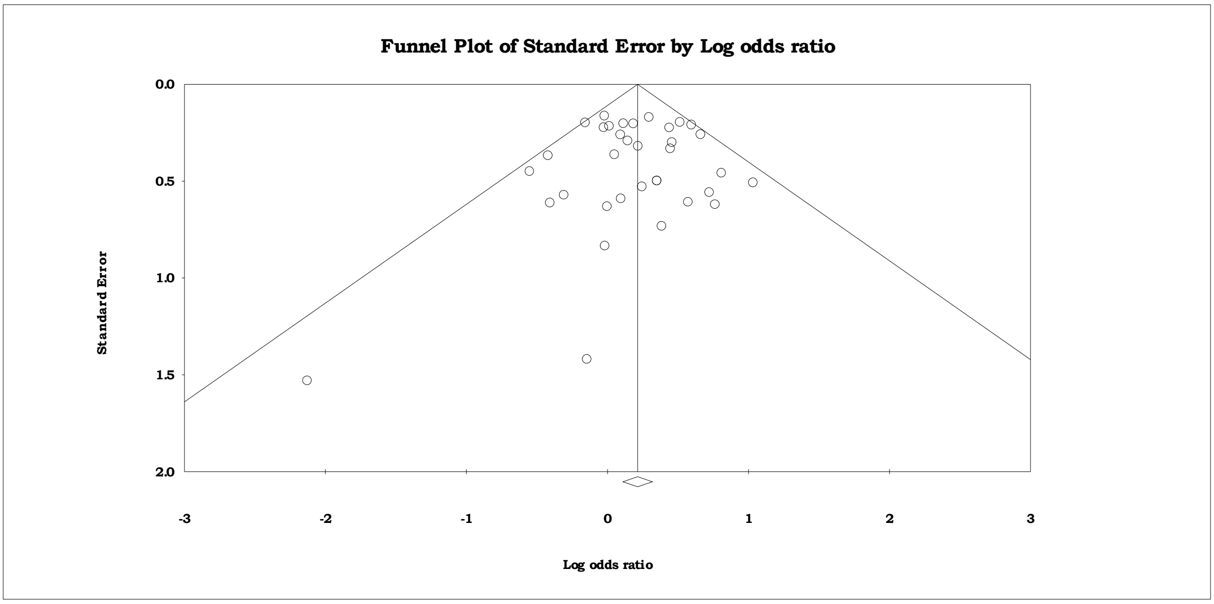

Funnel plot of the homozygous model with healthy subjects as control group in absence of heterogeneity

EXCLI Journal 2019;18:331-355 – ISSN 1611-2156
Received: March 06, 2019, accepted: April 09, 2019, published: June 07, 2019

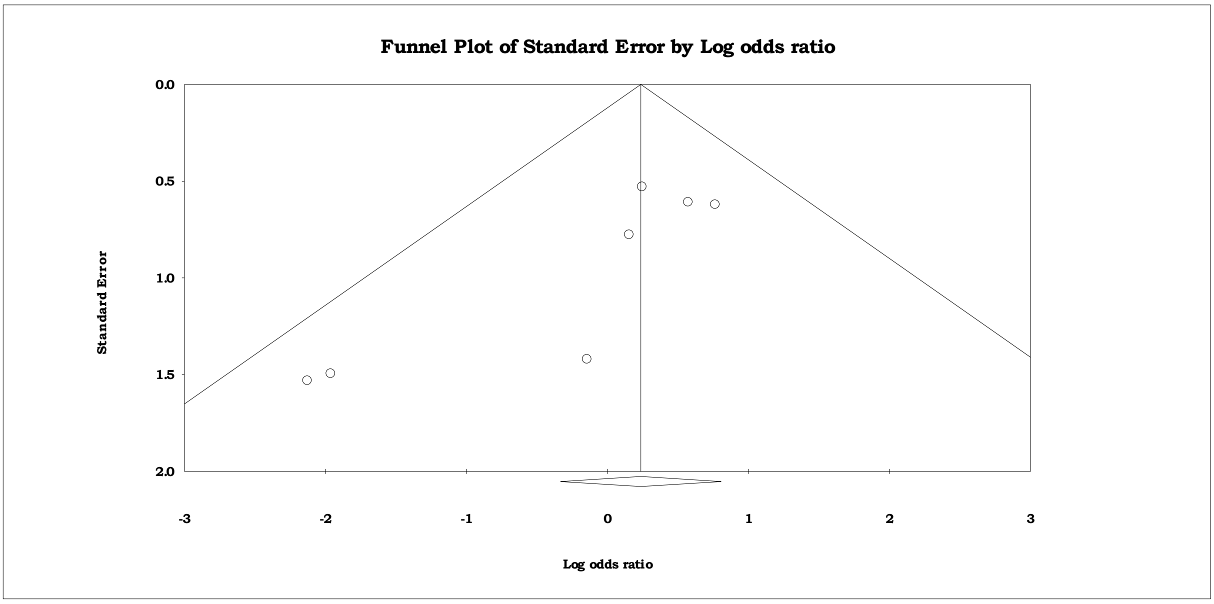

Funnel plot of the homozygous model in the Indian population group in absence of heterogeneity

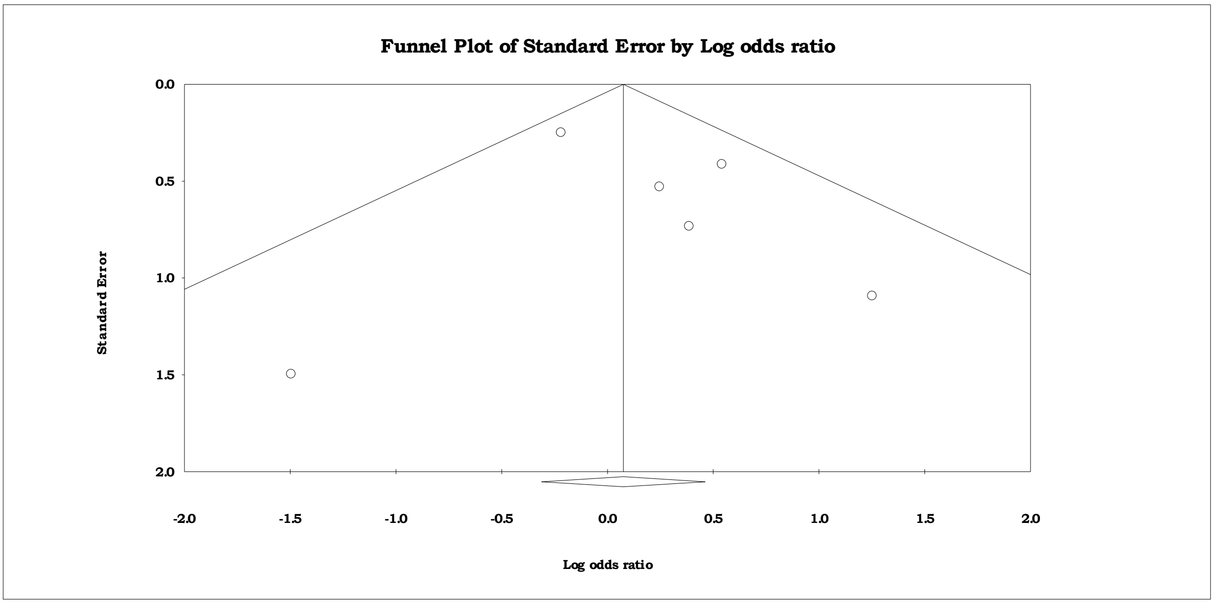

Funnel plot of the homozygous model in the IS population group in absence of heterogeneity

EXCLI Journal 2019;18:331-355 – ISSN 1611-2156
Received: March 06, 2019, accepted: April 09, 2019, published: June 07, 2019

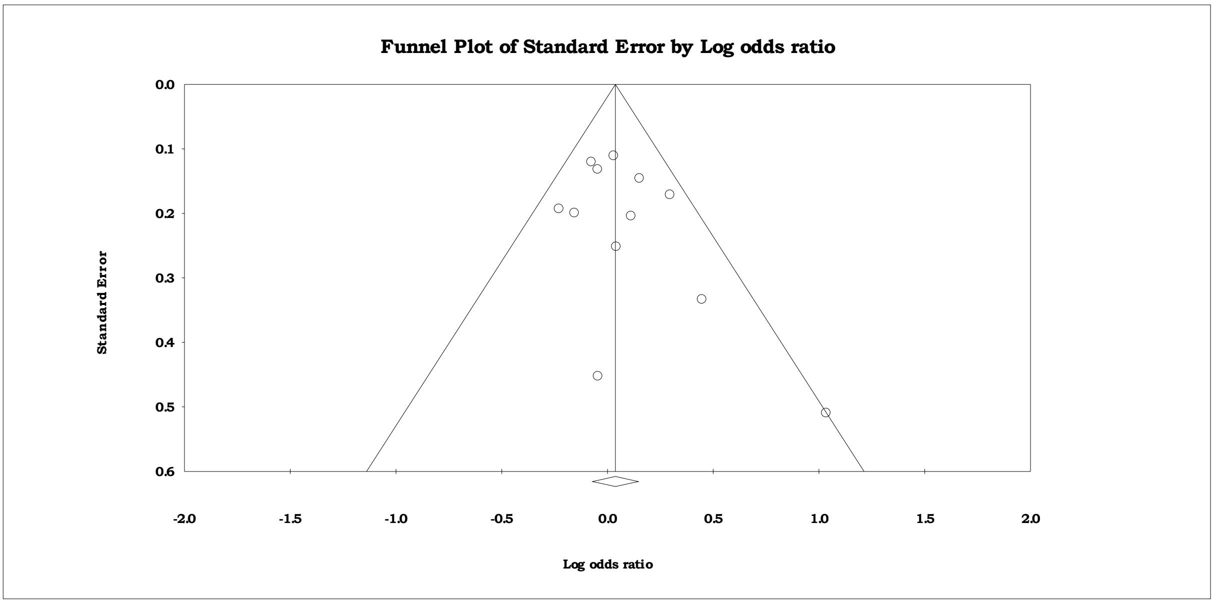

Funnel plot of the homozygous model in the MI population group in absence of heterogeneity

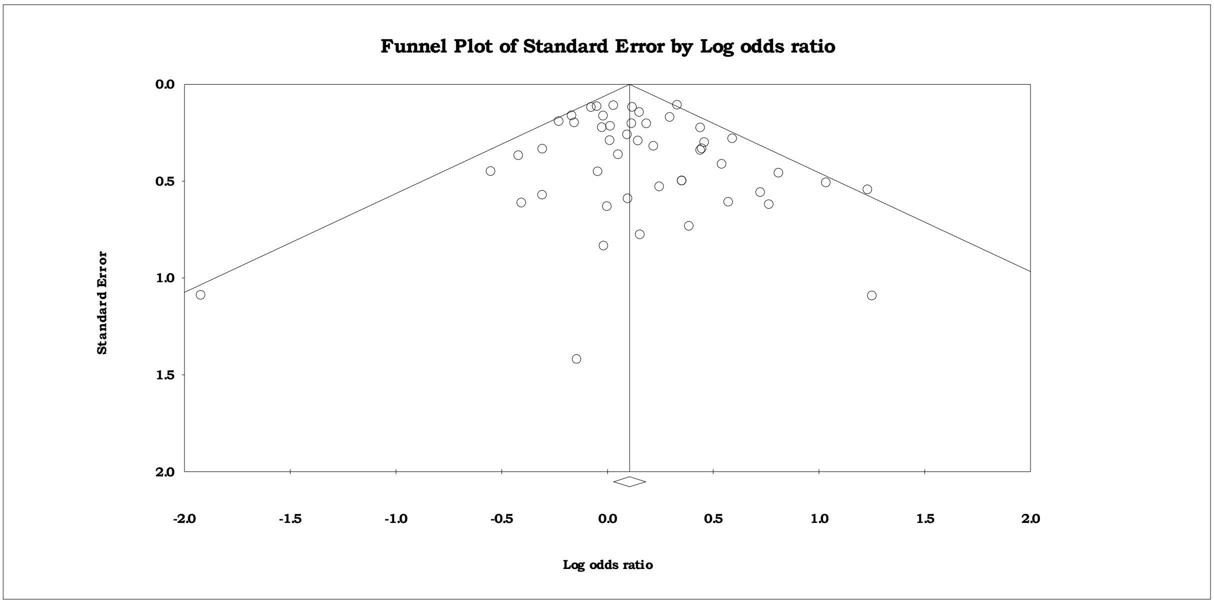

Funnel plot of the homozygous model in the Overall population group in absence of heterogeneity

EXCLI Journal 2019;18:331-355 – ISSN 1611-2156
Received: March 06, 2019, accepted: April 09, 2019, published: June 07, 2019

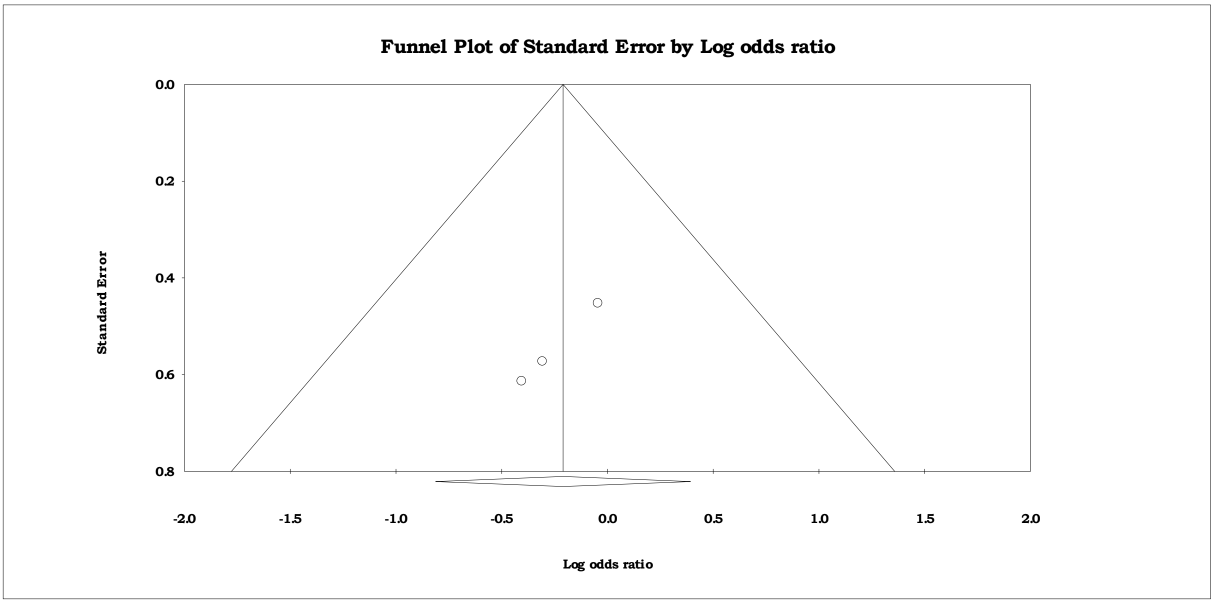

Funnel plot of the homozygous model in the Turkish population group in absence of heterogeneity

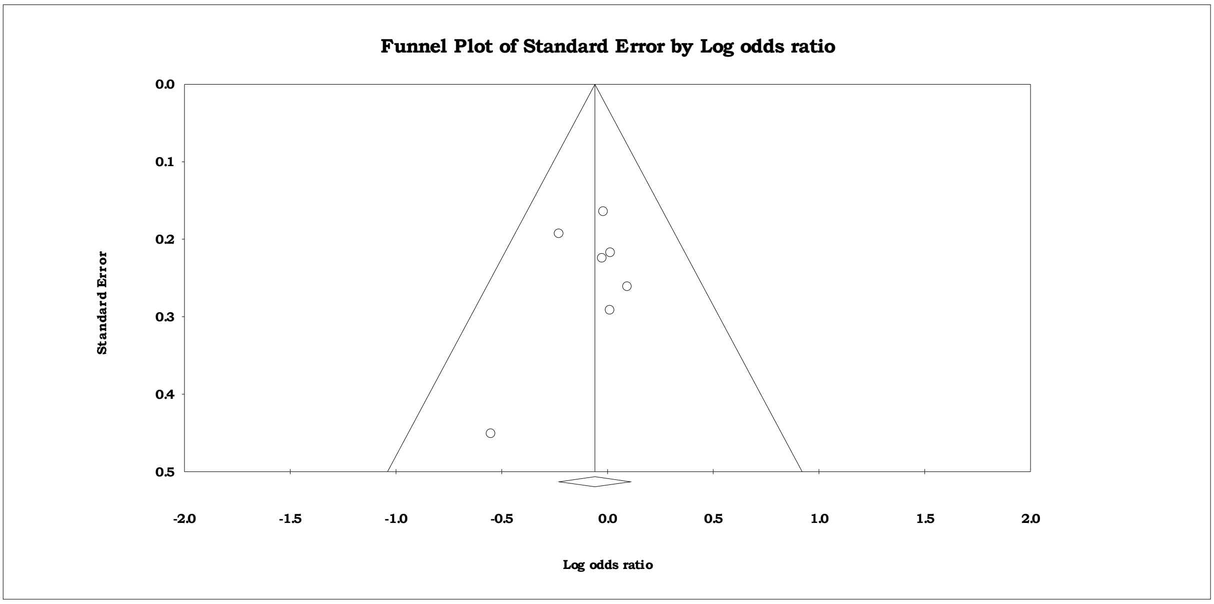

Funnel plot of the homozygous model in the UK population group in absence of heterogeneity

EXCLI Journal 2019;18:331-355 – ISSN 1611-2156
Received: March 06, 2019, accepted: April 09, 2019, published: June 07, 2019

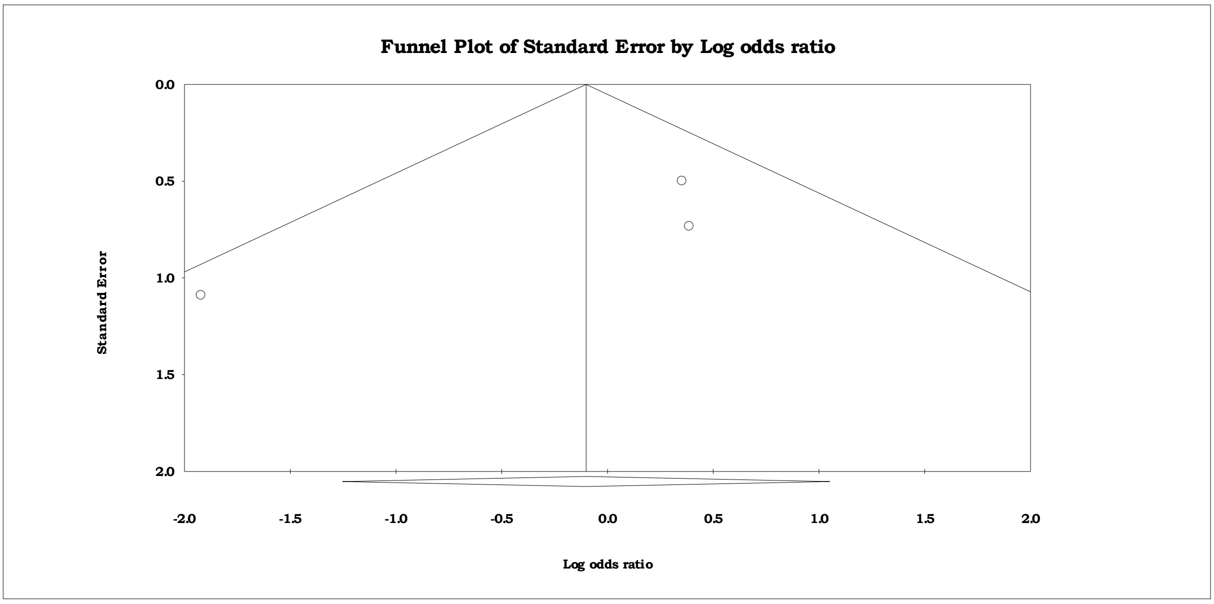

Funnel plot of the homozygous model in the African population group with low heterogeneity

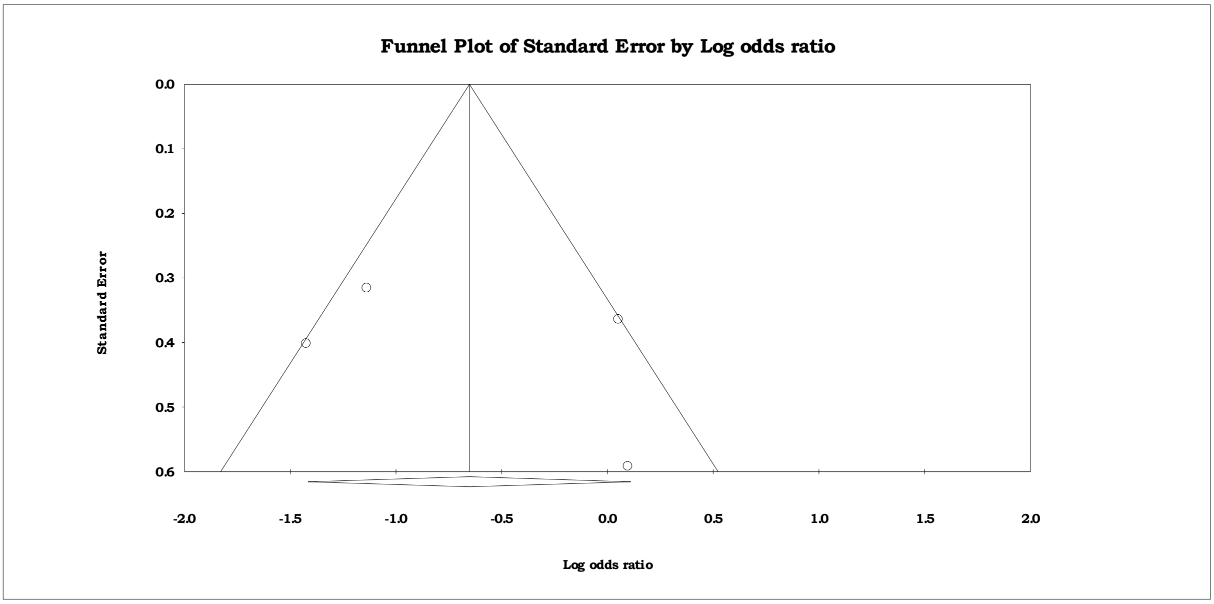

Funnel plot of the homozygous model in the PAOD population group with high heterogeneity

EXCLI Journal 2019;18:331-355 – ISSN 1611-2156
Received: March 06, 2019, accepted: April 09, 2019, published: June 07, 2019

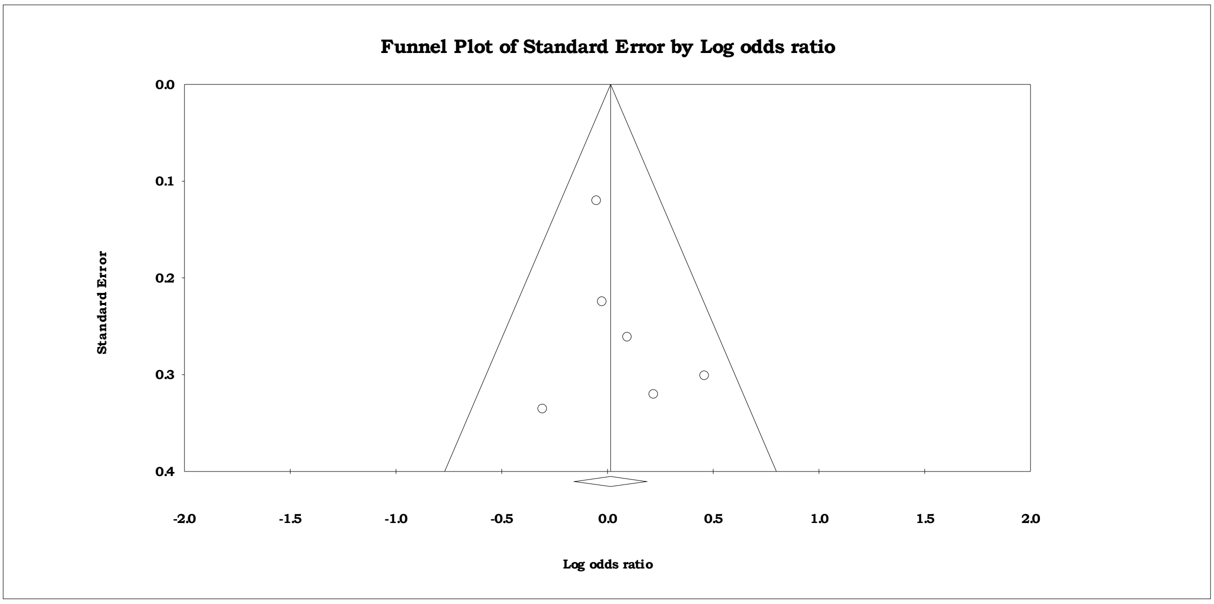

Funnel plot of the homozygous model in the European population with CAD in absence of heterogeneity

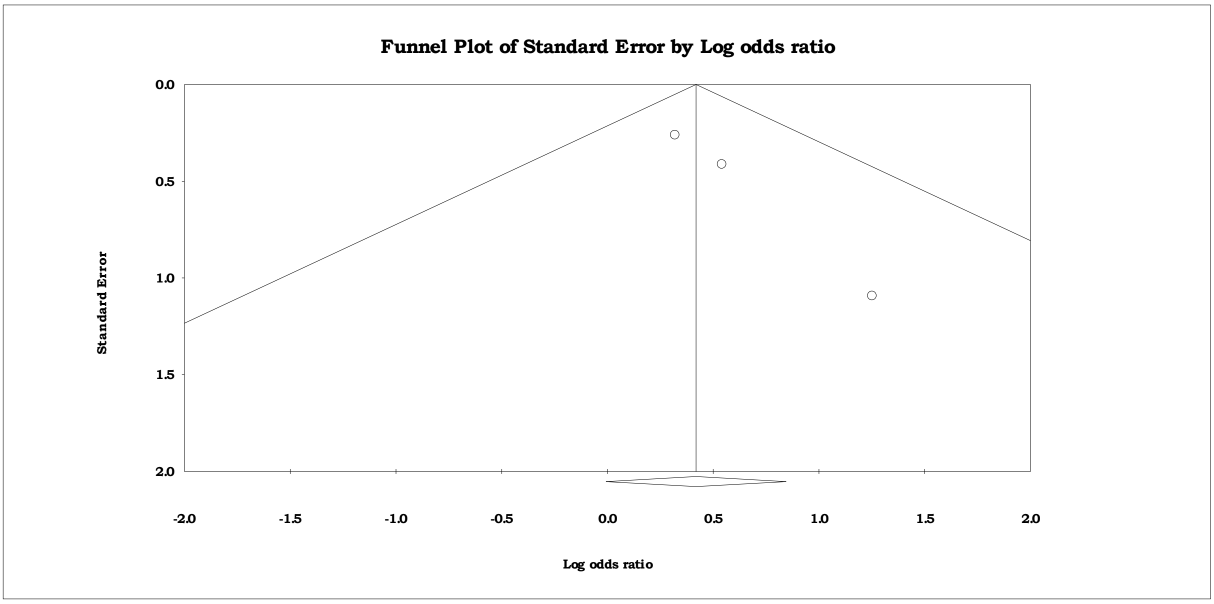

Funnel plot of the homozygous model in the European population with IS in absence of heterogeneity

EXCLI Journal 2019;18:331-355 – ISSN 1611-2156
Received: March 06, 2019, accepted: April 09, 2019, published: June 07, 2019

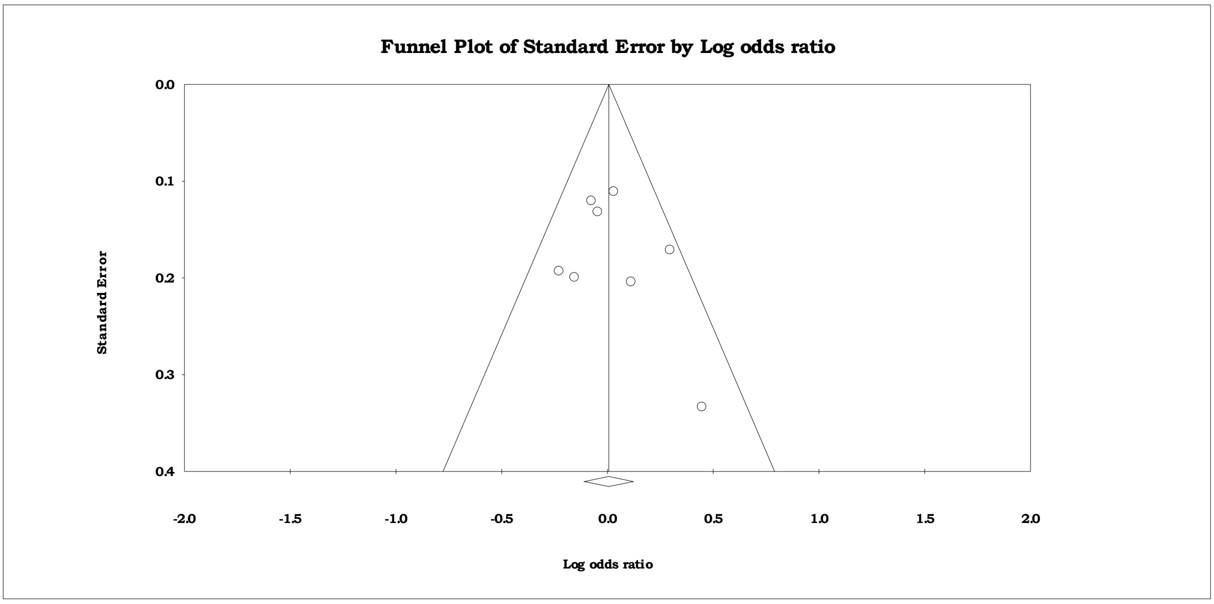

Funnel plot of the homozygous model in the European population with MI in absence of heterogeneity

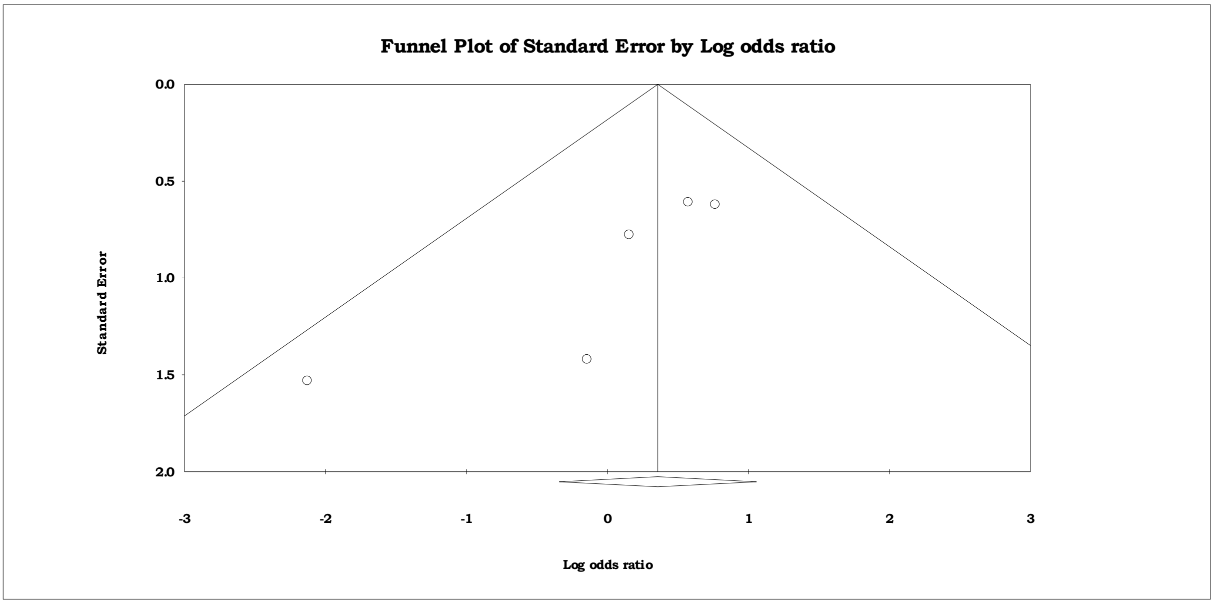

Funnel plot of the homozygous model in the Indian population with CAD in absence of heterogeneity

EXCLI Journal 2019;18:331-355 – ISSN 1611-2156
Received: March 06, 2019, accepted: April 09, 2019, published: June 07, 2019

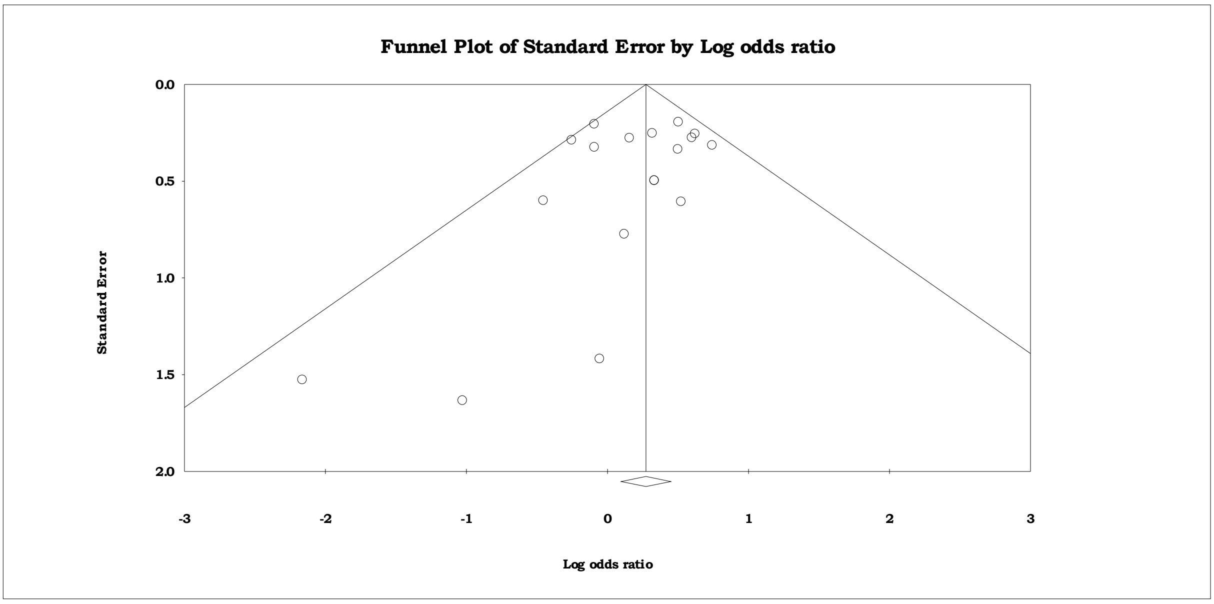

Funnel plot of the recessive model in the CAD population group in absence of heterogeneity

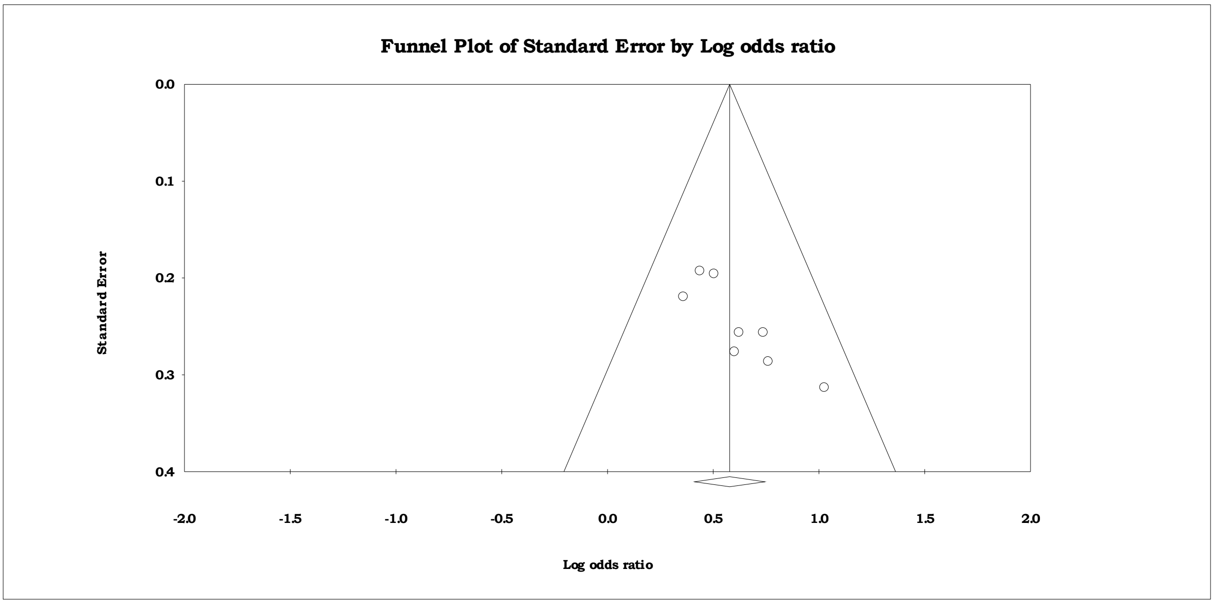

Funnel plot of the recessive model in the Chinese population group in absence of heterogeneity

EXCLI Journal 2019;18:331-355 – ISSN 1611-2156
Received: March 06, 2019, accepted: April 09, 2019, published: June 07, 2019

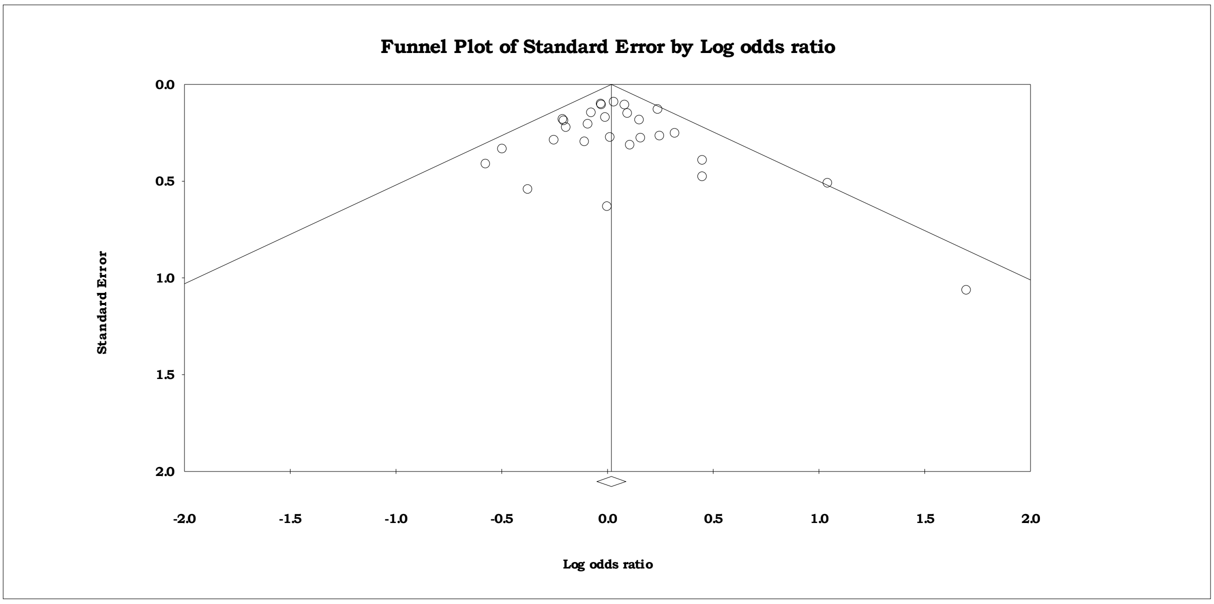

Funnel plot of the recessive model in the European population group in absence of heterogeneity

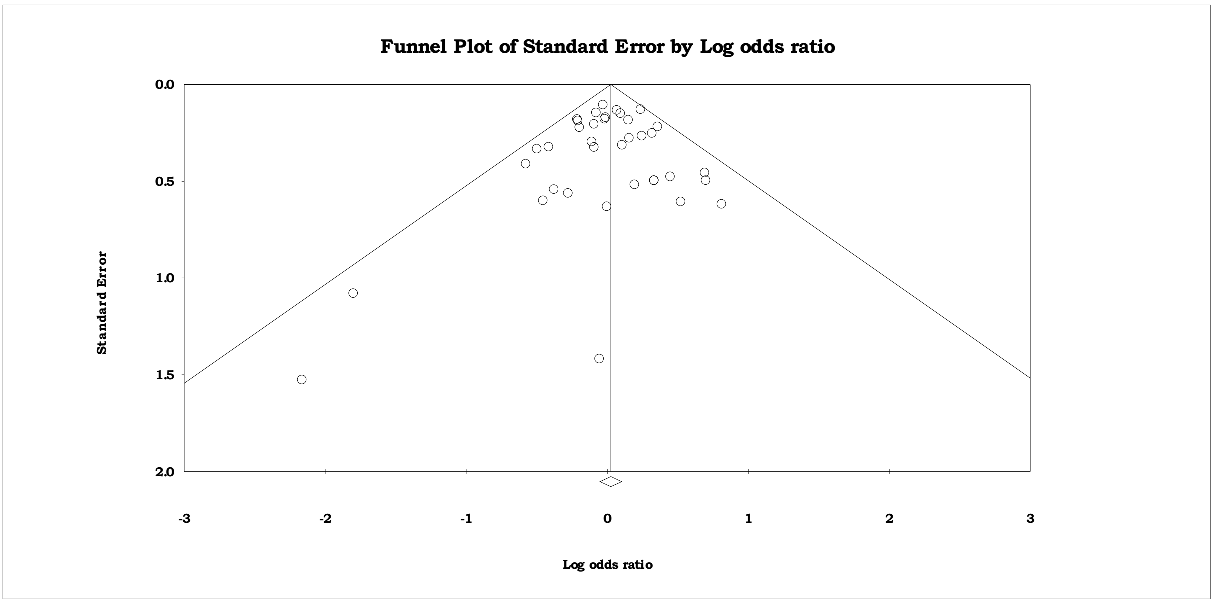

Funnel plot of the recessive model with healthy subjects as control group in absence of heterogeneity

EXCLI Journal 2019;18:331-355 – ISSN 1611-2156
Received: March 06, 2019, accepted: April 09, 2019, published: June 07, 2019

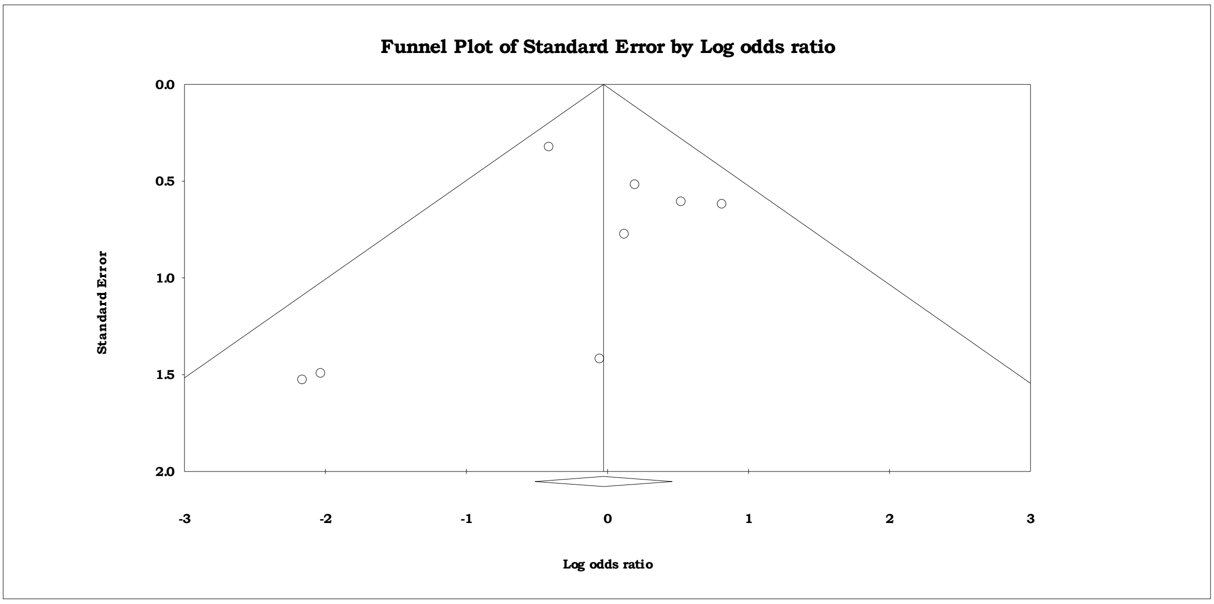

Funnel plot of the recessive model in the Indian population group in absence of heterogeneity

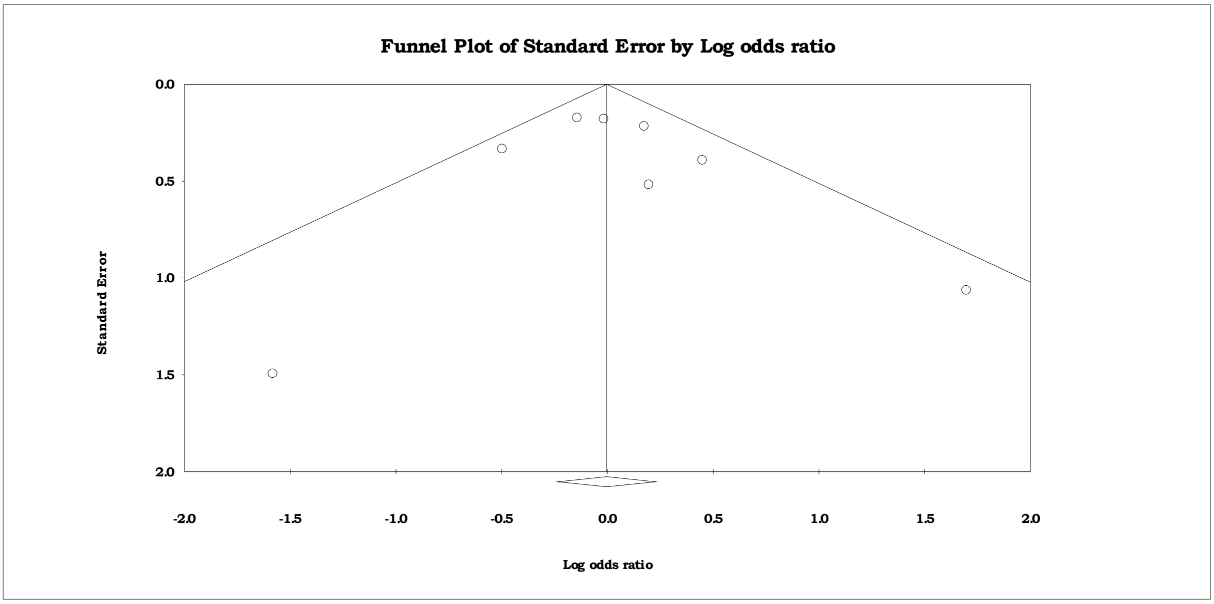

Funnel plot of the recessive model in the IS population group in absence of heterogeneity

EXCLI Journal 2019;18:331-355 – ISSN 1611-2156
Received: March 06, 2019, accepted: April 09, 2019, published: June 07, 2019

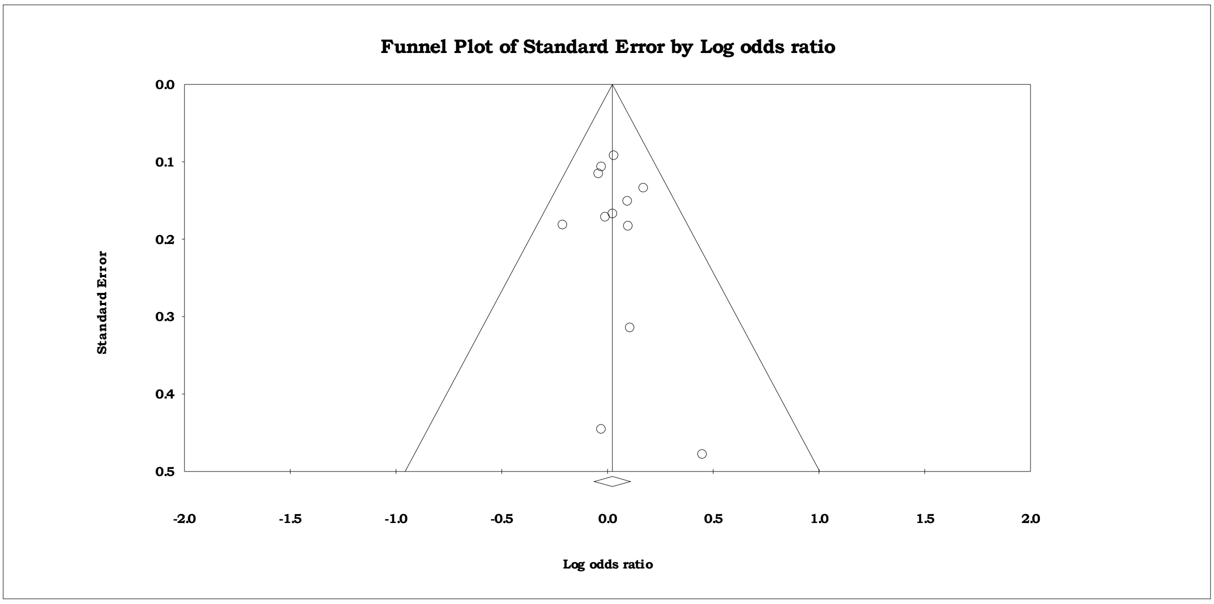

Funnel plot of the recessive model in the MI population group in absence of heterogeneity

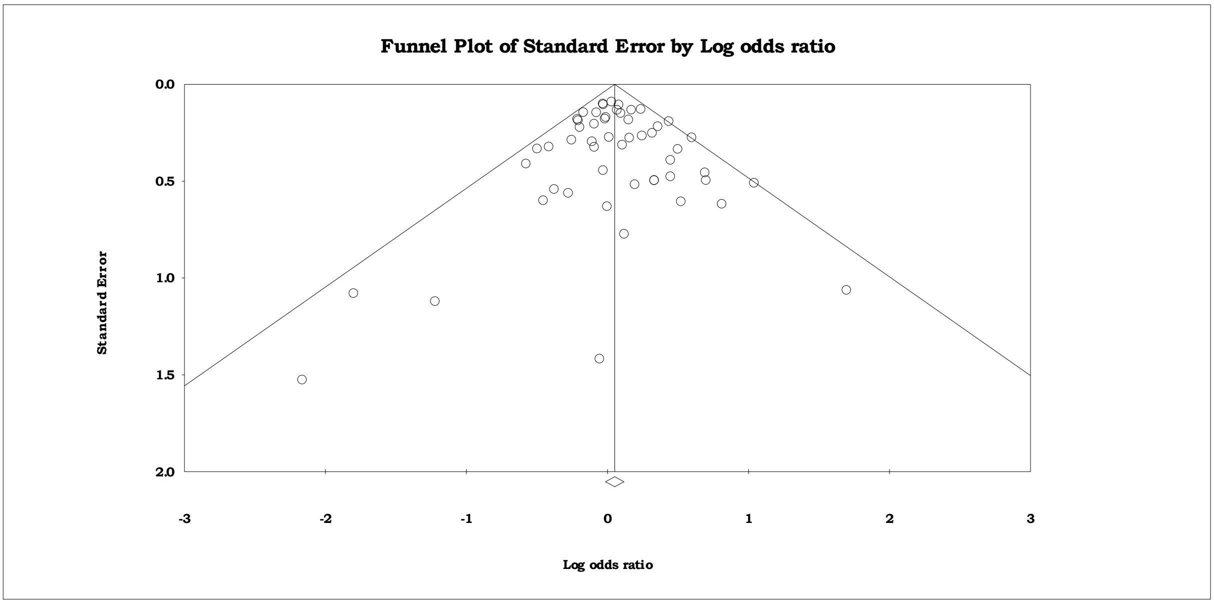

Funnel plot of the recessive model in the Overall population group in absence of heterogeneity

EXCLI Journal 2019;18:331-355 – ISSN 1611-2156
Received: March 06, 2019, accepted: April 09, 2019, published: June 07, 2019

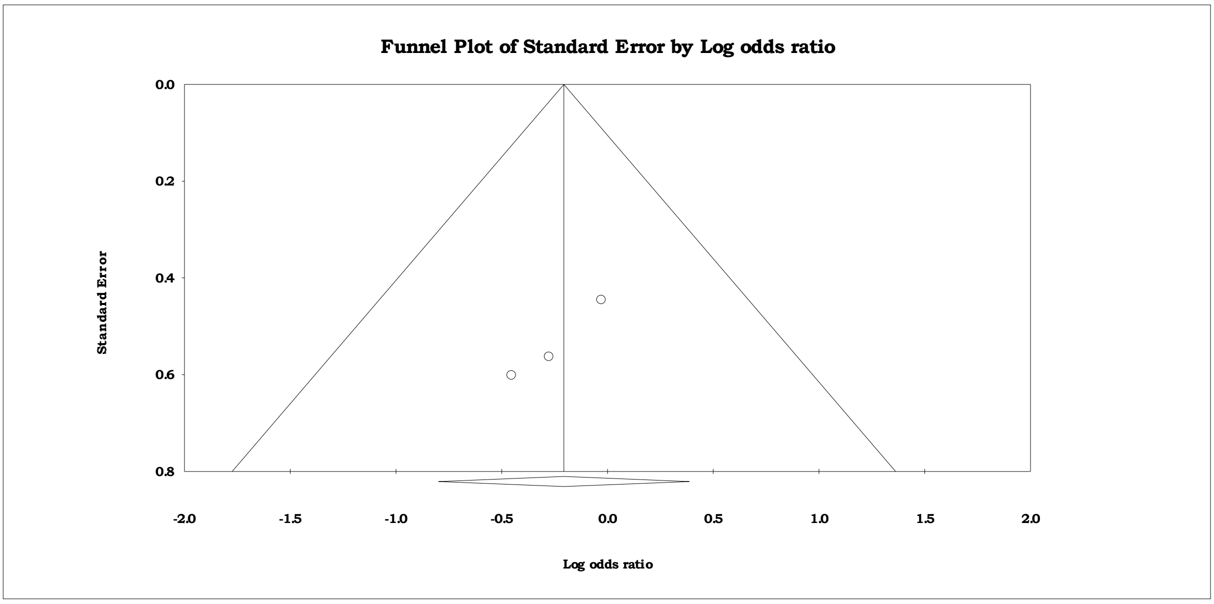

Funnel plot of the recessive model in the Turkish population group in absence of heterogeneity

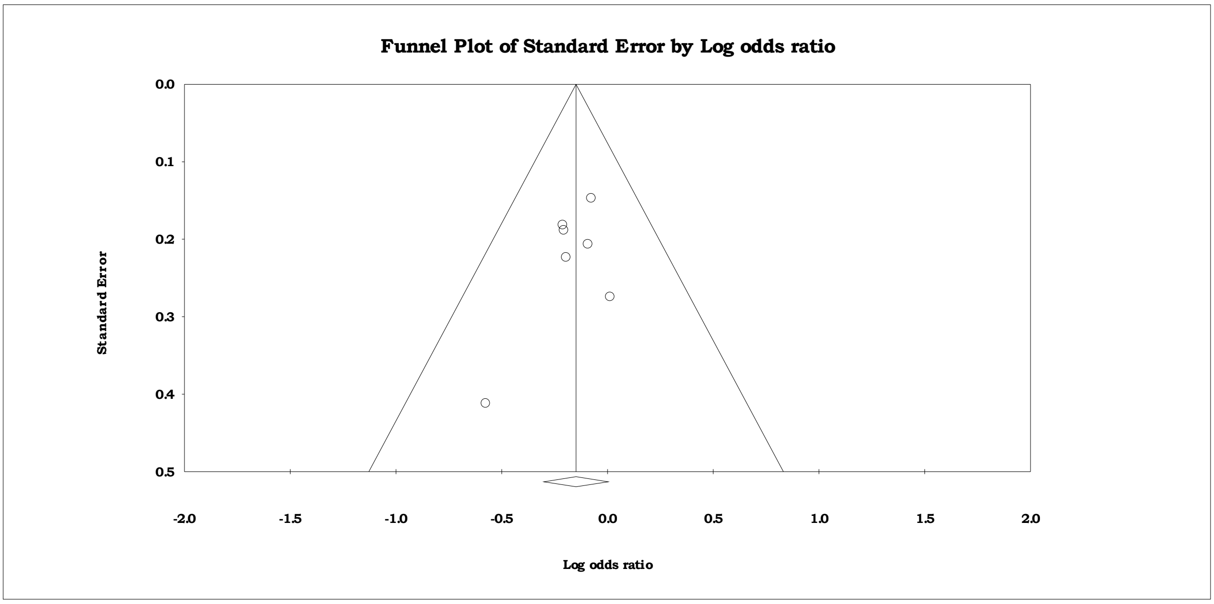

Funnel plot of the recessive model in the UK population group in absence of heterogeneity

EXCLI Journal 2019;18:331-355 – ISSN 1611-2156
Received: March 06, 2019, accepted: April 09, 2019, published: June 07, 2019

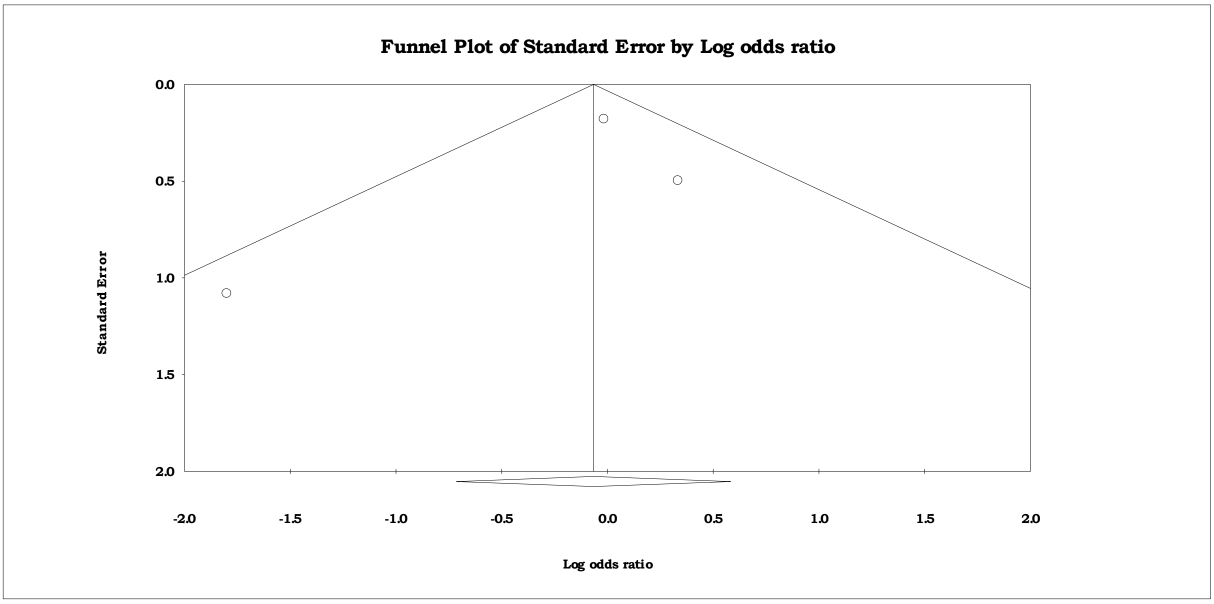

Funnel plot of the recessive model in the African population group with low heterogeneity

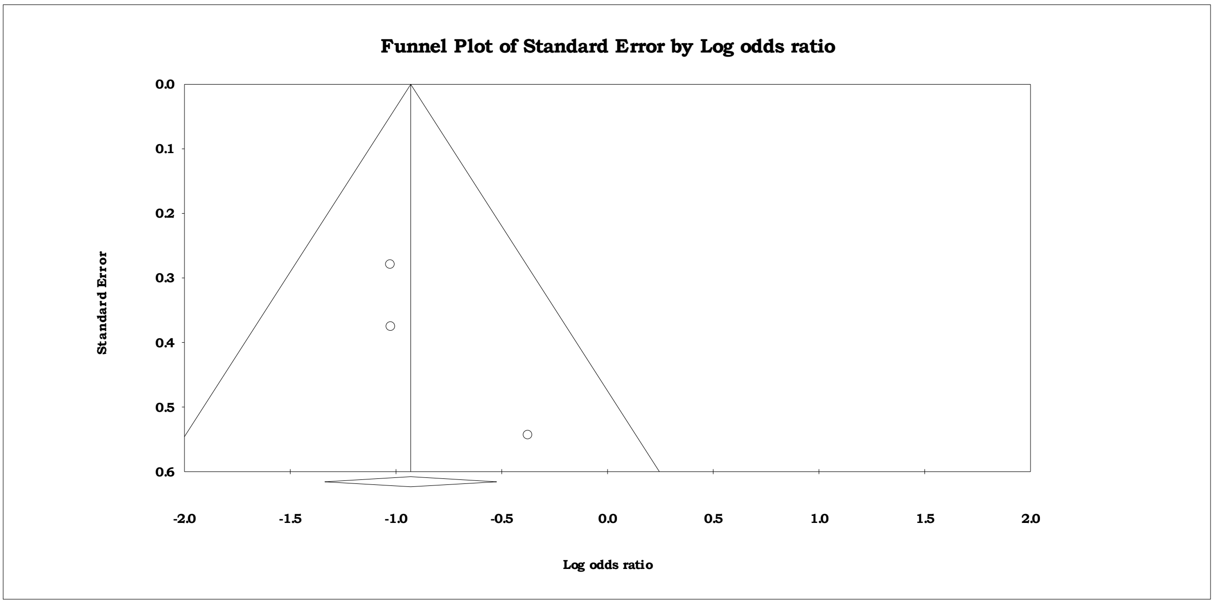

Funnel plot of the recessive model in the PAOD population group in absence of heterogeneity

EXCLI Journal 2019;18:331-355 – ISSN 1611-2156
Received: March 06, 2019, accepted: April 09, 2019, published: June 07, 2019

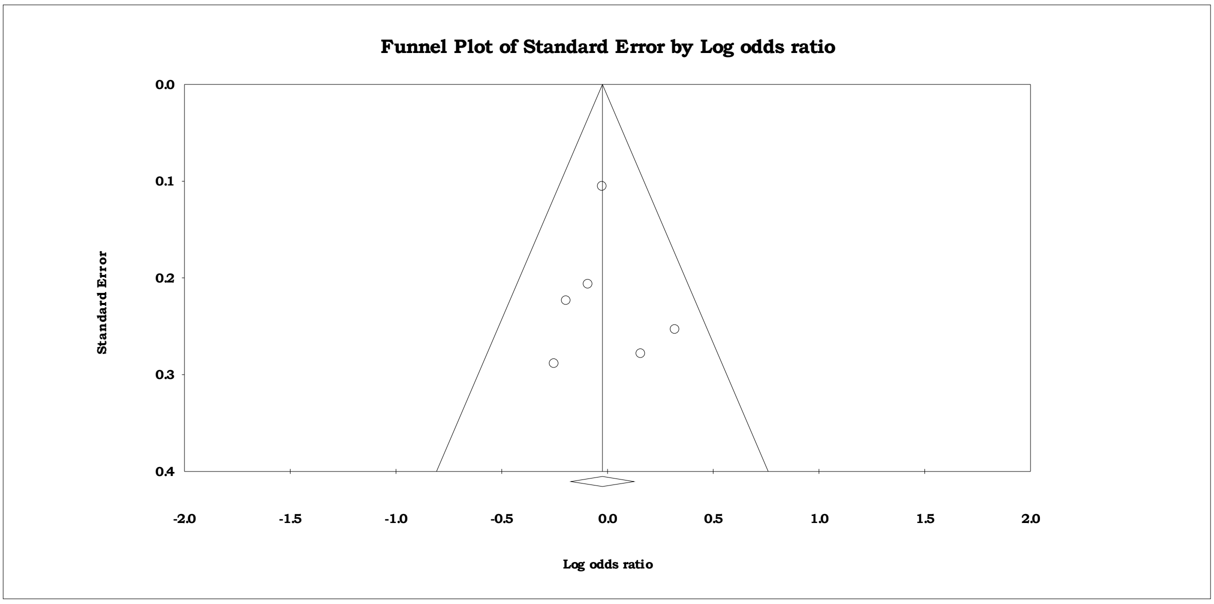

Funnel plot of the recessive model in the European population with CAD in absence of heterogeneity

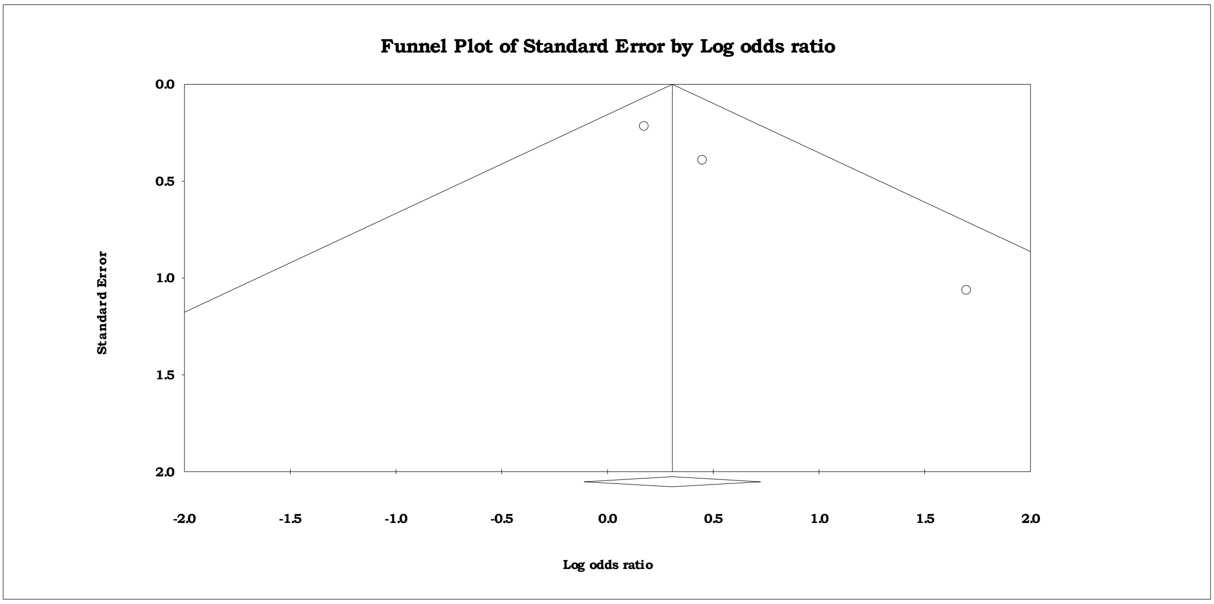

Funnel plot of the recessive model in the European population with IS in absence of heterogeneity

EXCLI Journal 2019;18:331-355 – ISSN 1611-2156
Received: March 06, 2019, accepted: April 09, 2019, published: June 07, 2019

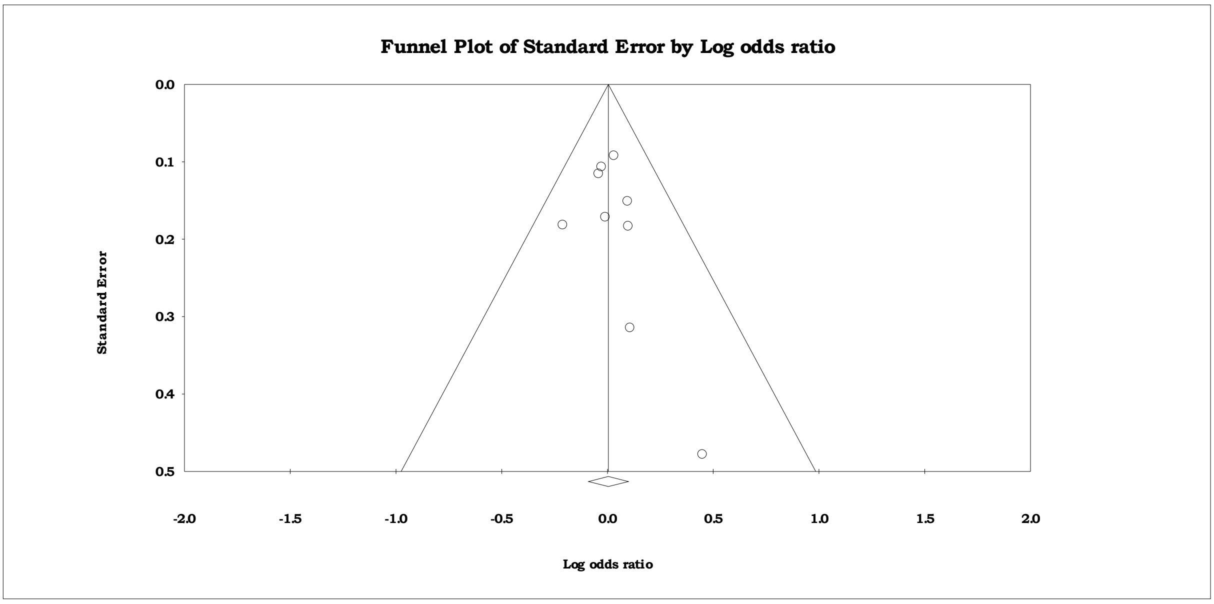

Funnel plot of the recessive model in the European population with MI in absence of heterogeneity

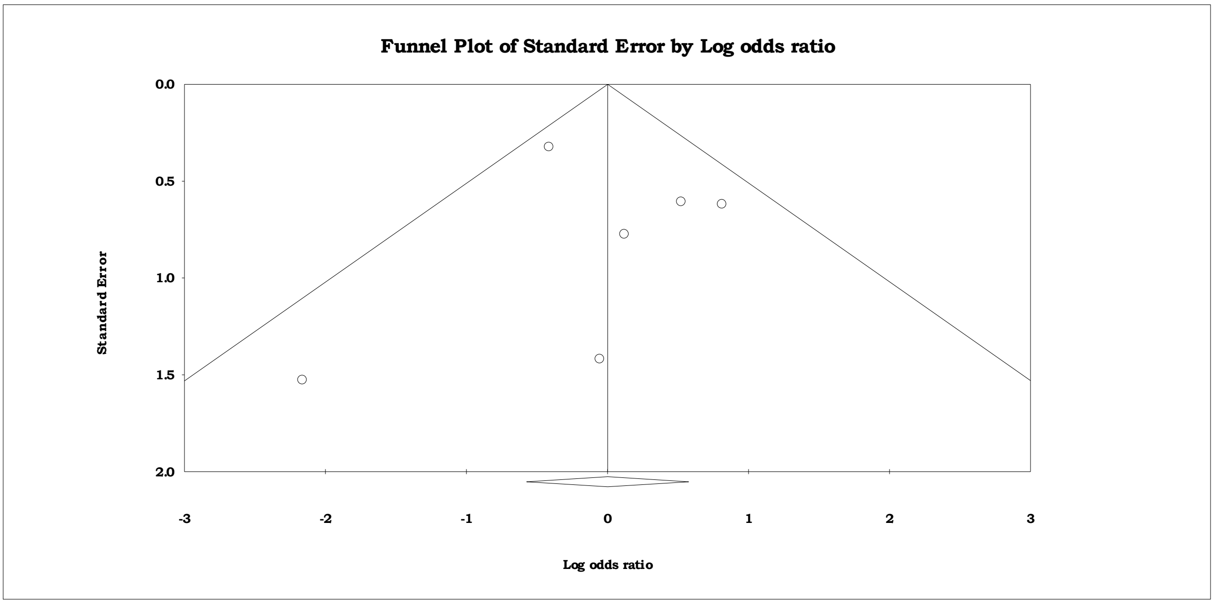

Funnel plot of the recessive model in the Indian population with CAD in absence of heterogeneity
